# The telomere-to-telomere (T2T) genome provides insights into the evolution of specialized centromere sequences in sandalwood

**DOI:** 10.1093/gigascience/giae096

**Published:** 2024-12-11

**Authors:** Dan Peng, Zhou Hong, Shenglong Kan, Zhiqiang Wu, Xuezhu Liao

**Affiliations:** Shenzhen Branch, Guangdong Laboratory of Lingnan Modern Agriculture, Key Laboratory of Synthetic Biology, Ministry of Agriculture and Rural Affairs, Agricultural Genomics Institute at Shenzhen, Chinese Academy of Agricultural Sciences, 518120 Shenzhen, China; Center for Genomics and Biotechnology, Haixia Institute of Science and Technology, Fujian Agriculture and Forestry University, 350002 Fuzhou, China; Research Institute of Tropical Forestry, Chinese Academy of Forestry, 510520 Guangzhou, China; Marine College, Shandong University, 264209 Weihai, China; Shenzhen Branch, Guangdong Laboratory of Lingnan Modern Agriculture, Key Laboratory of Synthetic Biology, Ministry of Agriculture and Rural Affairs, Agricultural Genomics Institute at Shenzhen, Chinese Academy of Agricultural Sciences, 518120 Shenzhen, China; Shenzhen Branch, Guangdong Laboratory of Lingnan Modern Agriculture, Key Laboratory of Synthetic Biology, Ministry of Agriculture and Rural Affairs, Agricultural Genomics Institute at Shenzhen, Chinese Academy of Agricultural Sciences, 518120 Shenzhen, China

**Keywords:** T2T genome, centromere, hemiparasitic species, cyto-nuclear transfer

## Abstract

**Background:**

Sandalwood, a prized hemiparasitic plant, is highly sought in the commercial market because of its aromatic core materia. The structure and stability of the genome are instrumental in the rapid adaptation of parasitic plants to their surroundings. However, there is a conspicuous lack of research on the genomic-level adaptive evolution of sandalwood.

**Results:**

In this study, we assembled a gap-free telomere-to-telomere (T2T) reference genome for *Santalum album* using PacBio HiFi, Hi-C, and ultra-long ONT data. The T2T reference genome (Sal_t2t) encompassed annotations of 24,171 genes and 25.34% repetitive sequences, in addition to all 10 centromeres and 20 telomeres across the 10 chromosomes. The results revealed that the 3 distinct parasitic species of Santalales had diverse centromeric compositions. The *Copia*-type long terminal repeat transposon emerged as the most significant in the *S. album* genome, constituting the primary sequence of the centromere and influencing gene expression. Third, in sandalwood, the presence of *Copia* affected the size of the centromeres and, consequently, the genome size. Identification of the sandalwood T2T genome in this study also enabled the identification of more precise organelle transfer fragments.

**Conclusions:**

Our research provides a sandalwood T2T genome, laying the groundwork for future investigations on the evolution of energy organs in parasitic plants. Moreover, it offers novel insights into the function and evolution of centromeres, as well as the mechanisms of adaptation and parasitism.

## Introduction

With the advent of long-read-length sequencing technologies and improved algorithms, genome assembly has entered a new era of telomere-to-telomere (T2T) assembly [1–3]. Compared to genomes with gaps, T2T genomes consist of minimal or no unassembled regions. They contain comprehensive information about telomeres, centromeres, ribosomal RNA (rDNA), complex chromosomal regions, and intracellular gene transfer (IGT) [4]. These areas are often challenging to handle, and complete assemblies may offer a deeper understanding of the structure and function of these unassembled regions. The T2T genome of *Arabidopsis thaliana* reported in 2021 facilitated the exploration of its centromeric genetic and epigenetic characteristics, revealed the mechanism of centromere evolution driven by the homologation of satellite sequences and retrotransposons, and marked the first application of T2T assembly technology in plants [5]. Since then, the sequence composition and evolution of centromeres have been elucidated by T2T genomes in several species, including rice, kiwifruit, watermelon, grape, carnation, and *Peucedanum praeruptorum*, among others [6–11]. However, the number of T2T genomes published to date is limited.

The centromere is a vital structure in eukaryotic chromosomes and plays an indispensable role in cell division [12]. Centromere dysfunction often leads to incorrect chromosome segregation during cell division, which can affect growth and development [13]. For instance, in plants, abnormal centromere function can result in stunted growth and development [14]. The structural and functional elucidation of the centromere is not only a fundamental scientific issue in the field of chromosome biology but also a cornerstone for the advancement of synthetic biology. Studies on the composition, structure, and evolution of centromeric sequences are key to elucidating their functions. However, the high number of repetitive sequences in the centromere presents a challenge for precise assembly and functional resolution. Generally, plant centromeric DNA sequences comprise 3 types: tandem repeats (TRs), centromeric retrotransposons (CRs), and a few functional genes with transcriptional activity [9, 15, 16]. CRs are typically interspersed with TRs and are abundant in plant centromeric regions, which are depicted as blank regions in the Hi-C contact heatmaps. Therefore, the T2T genome provides a more accurate sequence foundation for the identification of these signature sequences. However, most studies have been confined to crops or widely recognized horticultural plants, with few reports on the sequence characteristics of the centromeres of certain plant taxa such as parasitic plants. In contrast, maintaining chromosomal stability in parasitic plants is essential for their survival and reproduction, as they may need to adapt rapidly to changes in the host plant or environment. For instance, in the parasitic genus *Cuscuta*, the form of the centromeres is believed to be associated with its genome size and chromosome base, with monocentric *Cuscuta* species having a 102-fold variation in genome size and holocentric species having moderately sized genomes [17]. Moreover, to accommodate their parasitic lifestyle, the organelles of the parasitic plant may have undergone specific adaptive modifications, particularly cytonuclear interactions or transfers [18]. Therefore, the complete genomes of parasitic plants are required for relevant studies.

*Santalum album*, also known as sandalwood, is a valuable hemiparasitic plant belonging to Santalaceae. Sandalwood, an evergreen tree found across Southeast Asia, Australia, and the Pacific islands, is known for its medicinal properties, such as antimicrobial, antioxidant, and anti-inflammatory effects, and is commercially prized for its aromatic core materials [19, 20]. Its essential oil, termed “liquid gold,” is used in perfumes, cosmetics, and incense. Its hard texture and beautiful grains also make sandalwood ideal for carving and furniture [21]. Interestingly, Santalales not only encompasses hemiparasitic plants, such as sandalwood, but also includes nonparasitic (*Malania oleifera*), holoparasitic (*Balanophora subcupularis*), and other hemiparasitic species (*Taxillus chinensis*) [22–24]. Parasitic plants typically display greater genomic sequences and structural differences than nonparasitic plants, as an adaptation to their environment [25]. Previous studies have reported the assemblies of 2 chromosome/contig-level genomes with dozens to hundreds of gaps for sandalwood [26, 27]. Thus, there remains a research gap regarding the sequence differences between parasitic and nonparasitic plants in Santalales. Therefore, it is imperative to obtain a high-quality T2T genome for further investigation of unknown information in sandalwood.

In this study, we successfully obtained the T2T genome of *S. album*, with a size of 218.90 megabase pairs (Mb), comprising 10 chromosomes and no gaps. We identified all the centromeric and telomere sequences across the chromosomes. Our findings revealed that 3 species in Santalales with different parasitic forms exhibited differences in centromere composition and that *Copia*-type long terminal repeats (LTRs) significantly influenced *S. album* gene expression. In summary, this study represents the first T2T assembly of the *S. album* genome and provides an opportunity to investigate the genome structure and function of Santalales species.

## Results

### The gap-free genome assembly, completeness evaluation, and annotation for *S. album*

**Figure 1:**
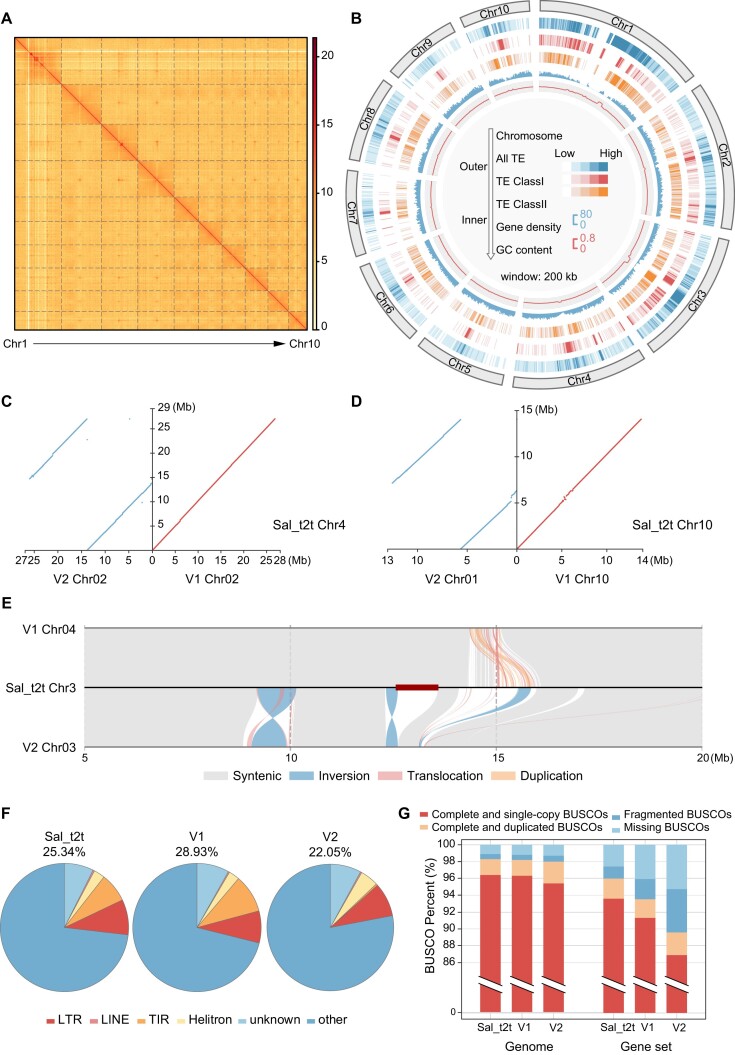
Assembly and annotation of Sal_t2t. (A) Heatmap of genomic interactions of Sal_t2t genome. (B) Characterization of Sal_t2t genome. The density of genes, TEs, and GC content was calculated per 200 kb. (C) Translocations in Chr4 (Sal_t2t) versus Chr02 (V2). Dots and lines represent chromosomes alignments between Sal_t2t, V1 (red), and V2 (blue). (D) Translocations in Chr10 (Sal_t2t) versus Chr01 (V2). The other contents were the same as panel A. (E) Alignments between Chr3 (Sal_t2t), Chr04 (V1), and Chr03 (V2) from 5 to 20 Mb. Deep red thick lines represent the centromere region. (F) Proportion of TE elements in 3 sandalwood assemblies. (G) BUSCO assessment of genomes and gene sets in 3 sandalwood assemblies.

By integrating 19.91 gigabase pairs (Gb, ∼90× coverage) of HiFi reads, 98.13 Gb (∼430× coverage) of Hi-C data, and 35.02 Gb (∼160× coverage) of ultra-long ONT reads, we obtained a gap-free sandalwood genome (named Sal_t2t) with a size of 218.90 Mb, comprising 10 chromosomes. The longest chromosome measures 34.97 Mb, and the shortest is 14.10 Mb (Fig. 1A, Table 1, and Supplementary Table S1). Compared with the 2 previously published versions of the sandalwood genome (V1: 23 gaps; V2: 108 gaps), all gaps in Sal_t2t were filled. Additionally, all structural variations (SVs) and presence/absence variations (PAVs) that differed from the previous two assemblies in complex regions or diversity between haplotypes were supported by more than 3 long reads (HiFi or ultra-long ONT reads) in the Sal_t2t genome (Fig. 1C–E, Supplementary Fig. S1). The contig N50 length of the Sal_t2t genome (18.40 Mb) is 1.44–3.31 times greater than that of V1 (12.75 Mb) and V2 (5.56 Mb) genomes, demonstrating a significant improvement in continuity and completeness in the newly assembled Sal_t2t (Table 1).

**Table 1:** Comparison of 3 sandalwood assemblies

	Sal_t2t	V1 (Hong)	V2 (Zhang)
Total length (Mb)	218.90	229.60	207.45
Gaps	0	23	108
Contig N50 (Mb)	18.40	12.75	5.56
BUSCO—genome	C:98.3% [S:96.4%, D:1.9%]	C:98.2% [S:96.3%, D:1.9%]	C:98.0% [S:95.4%, D:2.6%]
RNA reads mapping rate—Leaf	96.12%	96.05%	92.54%
NGS reads mapping rate	99.65%	98.81%	96.93%
HiFi reads mapping rate	99.97%	99.98%	99.48%
TE content	25.34%	28.93%	22.05%
TE content—*Copia*	6.63%	6.10%	5.73%
TE content—*Gypsy*	1.50%	1.09%	1.81%
Gene counts	24,171	21,673	23,282
BUSCO—gene set	C:96.0% [S:93.6%, D:2.4%]	C:93.5% [S:91.3%, D:2.2%]	C:89.6% [S:86.9%, D:2.7%]
rRNAs	3,910	7,720	610
tRNAs	567	614	506
Transfer count—mitochondria	2,416	2,400	2,246
Transfer count—chloroplast	2,620	2,515	2,299

Assembly accuracy was further assessed using several methods. First, the BUSCO assessment based on embryophyta_odb10 revealed that 98.3% of the core conserved plant genes (1,586/1,614 genes) were fully characterized in this genome. Second, the comparison showed that 99.65% of short reads, 99.97% of HiFi reads, and 96.12% of RNA sequencing data from leaf samples could be mapped onto the Sal_t2t genome. Furthermore, integrity checks of the LTRs revealed an assembled LTR assembly index (LAI) of 26.41. This genome has a consensus quality value (QV) of 67.30. Collectively, these data demonstrated the high accuracy of the Sal_t2t assembly (Table 1).

The Sal_t2t genome provides an unprecedented opportunity to identify all the repeat sequences and genes. Consistent with previous predictions, 25.34% (55.48 Mb) of the sequences were identified as transposable elements (TEs), with 19.54 Mb (8.93%) of retrotransposons and 23.04 Mb (10.51%) of DNA transposons in Sal_t2t (Fig. 1F and Supplementary Table S2). Retrotransposons and DNA transposons were predominantly distributed in the central regions of the chromosomes (Fig. 1B). We further predicted 24,171 protein-coding genes, 12 of which were either pseudogenes or incomplete (Table 1 and Supplementary Table S3). Moreover, the completeness of the annotations was assessed using BUSCO, revealing that approximately 98.3% and 96.0% of the core genes in the assembly and gene set were complete, which was much higher than those of the V1 (98.2% and 93.5%) and V2 (98.0% and 89.6%) versions (Fig. 1G and Table 1). A total of 567 transfer RNA (tRNA), 332 small nuclear RNA (snRNA), and 3,910 rRNA sequences were annotated and predicted in the sandalwood genome using the Rfam database (Table 1 and Supplementary Table S4). Among these, 3,324 were annotated as 5S rRNA, accounting for 85.01% of the total rRNA count, primarily distributed in the genomic region of 21.72–23.38 Mb on Chr1.

### Identification of new genes

To test whether different assembly methods selectively enrich certain types of genes, we analyzed the differences between the annotation results of the 2 versions, Sal_t2t (Sal_ano) and V1 (V1_ano). Using Liftoff software with Sal_ano and V1_ano as references, 21,583 and 24,007 genes were annotated to the genomes of V1 and Sal_t2t, respectively. Sal_ano contained 1,282 previously unannotated genes, while V1 contained 2,583 genes, which may be due to differences in annotation software and parameters. In addition, the *de novo* Sal_t2t genome assembled 164 genes, whereas V1 had 92 genes, which were identified as new genes based on Liftoff software (Supplementary Table S5).

In addition, the 164 new genes identified in Sal_t2t were mainly enriched in the Gene Ontology (GO) terms oxidoreductase activity, biosynthetic processes, and metabolic processes (Supplementary Fig. S2). Of these 164 new genes, 29 were located in regions located in the filled gaps of V1, and 28 of these new genes were located in the filled regions enriched with rRNAs and TRs in Sal_t2t. These genes showed high similarity and were supported by ultra-long ONT reads, suggesting that they may be duplicated. Classification of all new genes by DupGen_finder software revealed 152 (92.68%) new genes were categorized (dispersed/proximal/tandem/transposed/wgd: 26/60/36/12/18), and the proportion of duplicated genes in the genome-wide background was 70.84%. Moreover, 56 duplicate genes (dispersed/proximal/tandem/transposed/wgd: 4/18/18/5/11) were expressed (Supplementary Table S6).

### Organelle gene transfer

**Figure 2:**
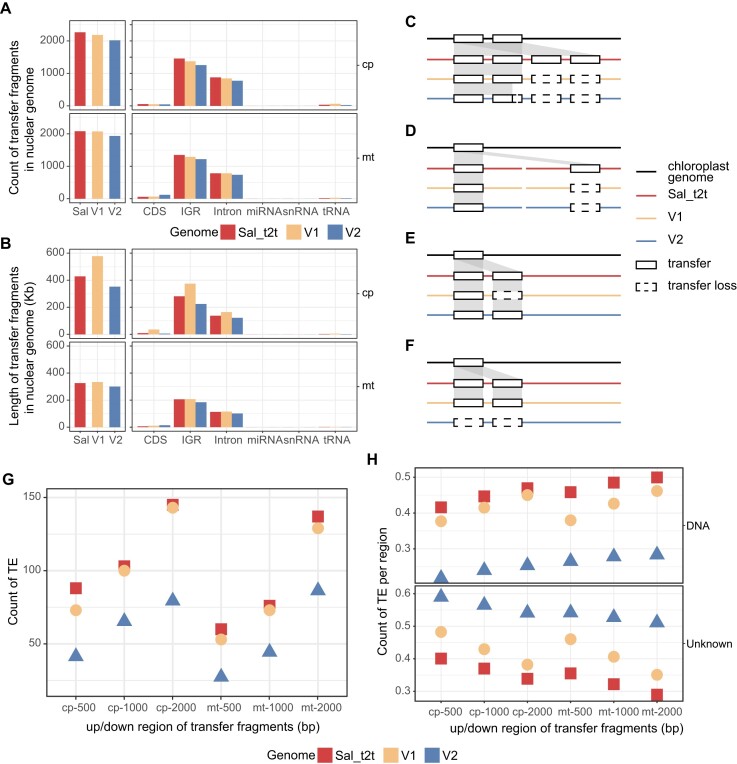
Organelle gene transfer. (A) Counts of NUPTs and NUMTs in 3 sandalwood assemblies. (B) Length of NUPTs and NUMTs in 3 sandalwood assemblies. (C–F) Simplified comparison of the chloroplast genome and 3 nuclear genome assemblies: (C) overall deletion of a neighboring chloroplast fragment transferred multiple times; (D) partial copy number deletion of a chloroplast fragment transferred to a different chromosome; (E) partial copy number deletion of a chloroplast fragment transferred to the same chromosome; (F) complete deletion of a chloroplast fragment transferred to a chromosome. (G) Counts of intact TEs in flanking regions of transfer fragments. (H) Counts of DNA and unknown type of TEs in flanking regions of transfer fragments.

The integrity of organelle transfer fragments, such as nuclear integrants of mitochondrial DNA (NUMTs) and nuclear integrants of plastid DNA (NUPTs), also serves as an index for evaluating assembly quality [28]. By assembling the nuclear, mitochondrial, and chloroplast genomes of *S. album*, we evaluated the frequency and patterns of NUPTs and NUMTs among the Sal_t2t, V1, and V2 genomes and found similar quantity and length distribution patterns. However, more accurate organelle transfer fragments were identified in Sal_t2t than in the other 2 versions, especially in the intergenic region (IGR) (Fig. 2A, B and Supplementary Table S7).

In comparison with the ultra-long ONT sequences, we also identified a misassembly of the nuclear genome involving 5 NUPTs larger than 10 kilobase pairs (kb), which was corrected for Sal_t2t (Supplementary Fig. S3). Additionally, we observed that regions where the same chloroplast fragment was transferred multiple times to the nuclear genome might also have been misassembled, including redundancy in low-copy chloroplast transfer fragments and partial or complete loss of multicopy chloroplast transfer fragments (Supplementary Tables S8, S9). Sequence alignment verification results also demonstrated the integrity and accuracy of the transfer fragment assembly in the T2T genome (Fig. 2C–F).

We also examined the quantity of transposons upstream and downstream of the organelle transfer fragments and found that the V2 and V1 genomes had fewer transposable elements surrounding the organelle transfer fragments, whereas Sal_t2t had the highest number of intact TEs (Fig. 2G and Supplementary Fig. S4). Further categorization revealed that the quantities of LTR, MITE, TIR, LINE, and Helitron transposons were similar among the 3 versions, but there were significant differences between DNA transposons and unknown transposons (Supplementary Fig. S5). In DNA transposons, the average content was the highest in Sal_t2t and lowest in V2, whereas the trend was reversed in unknown transposons (Fig. 2H). This indicates that the Sal_t2t genome provides a clearer and more accurate prediction of the distribution, types, and quantities of TEs surrounding the transfer fragments.

### Architecture and context of telomeres and centromeres

The completion and accuracy of genome sequencing have enabled the identification of telomeres and centromeres. First, the results of telomeric regions revealed that both ends of the 10 sandalwood chromosomes possessed telomere repeat units (AAACCCT/AGGGTTT), aligning with the telomere structure of most plants. The longest telomere, located on chromosome 1, measured 16.62 kb and contained 2,374 repeats, while the shortest telomere, found on chromosome 9, measured 1,869 base pairs (bp) with only 267 repeats (Supplementary Table S10).

**Figure 3:**
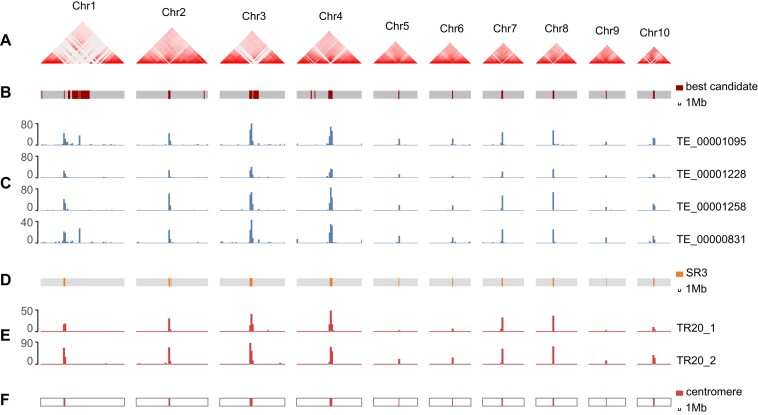
Characteristics and distribution of repeats in centromeres. (A) Heatmap of genomic interactions of each chromosome in Sal_t2t genome. (B) Best candidate regions predicted by quarTeT. (C) Distribution of CRs. (D) HOR regions predicted using SRF. (E) Distribution of TRs in HORs. (F) The final centromere regions.

In addition, we identified centromeric regions using quarTeT, combined with EDTA annotations and the blank regions in the Hi-C contact matrices as candidate centromeric regions (Fig. 3A, B). We quantified and visualized TEs in each chromosome’s candidate regions and observed that 3 *Copia* repeats (TE_00001095, TE_00001228, and TE_00001258) and 1 unknown LTR repeat (TE_00000831) were highly enriched in 8 candidate regions and 2 secondary candidate regions across chromosomes, whereas they were scarce on chromosome arms (Fig. 3C). We speculated that these 4 LTR sequences were CRs.

Second, 470 distinct TR units were identified in the Sal_t2t genome. A 312-bp repeat was the most abundant unit in the genome, with a total of 10,335 copies of ≥2 repeats, accounting for 1.47% of the entire genome sequence. This was followed by 500 bp (0.72%), 32 bp (0.42%), and 63 bp (0.30%). However, the top 20 units in the genome, in terms of total length and total copy number, lacked typical centromeric TR sequence distribution characteristics (Supplementary Fig. S6). Subsequently, 21 types of high-order repeat (HOR) regions were identified, among which SR3 (prefix#circ3-7198) was primarily composed of 2 tandem repeats of 20 bp in length (AGCCCAAGCACACTTGGAGG and TCCAAGTGTCATTGGGCTCC), which highly overlapped with the candidate regions and CRs (Fig. 2D, E and Supplementary Table S11). Therefore, we defined the distribution ranges of the CR and TR (from the Satellite Repeat Finder (SRF) results) as the centromeric regions of all chromosomes (Supplementary Table S12).

### Comparative analysis of centromeric sequences in Santalales

To study the differences and evolution of centromere sequences among various species of Santalales, we identified and analyzed the centromere sequences of available genomes of Santalales species. We focused on the CRs and TRs of *S. album, M. oleifera*, and *T. chinensis*. The genomes of *Balanophora* and *Scurrula* were excluded from the analysis because of the low quality of their assembly.

**Figure 4:**
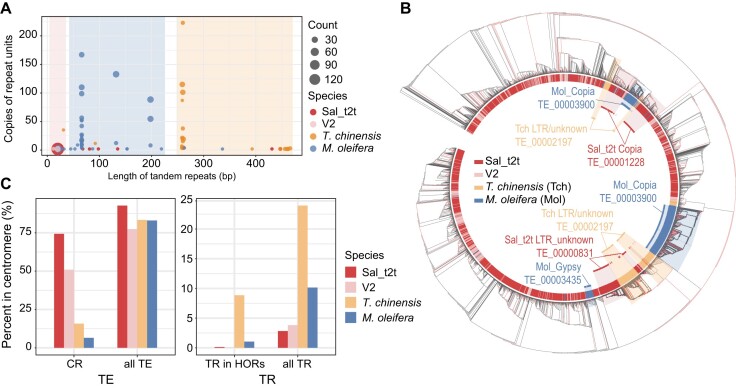
Comparison of centromeric repeats in Santalales. (A) Scatterplot of TRs in HORs of 4 Santalales genomes/assembles. Counts of each TR unit associated with circle sizes. Shadows in the background represent the roughly distributed areas of TRs in each species. (B) Phylogeny of CRs of 4 Santalales genomes/assembles without branch length. Different colors in the inner ring represent TE sequences in each species. (C) Proportions of TE and TR in centromere regions of 4 Santalales genomes/assembles.

In the Sal_t2t genome, the TRs consisted of two 20-bp sequences, a pattern similar to that observed in the V2 genome. These 20-bp sequences were found in low numbers in the centromeric region, with only 2 copies per repeat unit. The number of repeat units per chromosome ranged from 2 (Chr9) to 46 (Chr4), averaging 19.5 units per chromosome. We identified TRs in the same pipeline for the other 2 species and found that the length (259 and 260 bp) and copy number (101–223) of the TR repeat units in *T. chinensis* were significantly higher than those in the sandalwood genome. In *M. oleifera*, centromeric tandem repeats primarily consisted of 66-bp repeat sequences, with copy numbers ranging from 10 to 110 (Fig. 4A).

In terms of centromeric retrotransposon sequences, we identified 3 *Copia* sequences and 1 unknown LTR sequence in Sal_t2t, with one-fourth of the TE_00001095 sequence being intact LTRs. However, in *T. chinensis*, we identified only 1 unknown LTR sequence, whereas in *M. oleifera*, we identified 1 *Copia* sequence and 1 *Gypsy* sequence. We utilised these CRs to construct phylogenetic trees and observed that the LTR sequence TE_00001228 of sandalwood clustered with several LTR sequences of *T. chinensis* and *Copia* sequences of *M. oleifera* in the same clade, whereas the LTR sequence TE_00000831 of sandalwood belonged to the same clade as the majority of the LTR sequences of *T. chinensis* (Fig. 4B). Additionally, the complete LTR sequence TE_00001095 of sandalwood did not cluster with *T. chinensis* or *M. oleifera*, whereas the majority of *Copia* and *Gypsy* sequences of *M. oleifera* clustered separately into distinct clades. Furthermore, differentiation occurred in the same LTR classification in the same species, as evidenced by the differences in CR and TR content in these 4 species (Fig. 4C). These findings suggest that while centromeric TR and CR sequences are conserved in species, they differ significantly between species. However, owing to limitations in the quantity and quality of published genome assemblies, further analysis and comparison of telomeric regions may require additional T2T genome sequences from the same family or genus. This could be particularly relevant for assessing the conservation of the CR and TR sequences at different taxonomic levels.

### Relationship between centromere and chromosome characteristics

**Figure 5:**
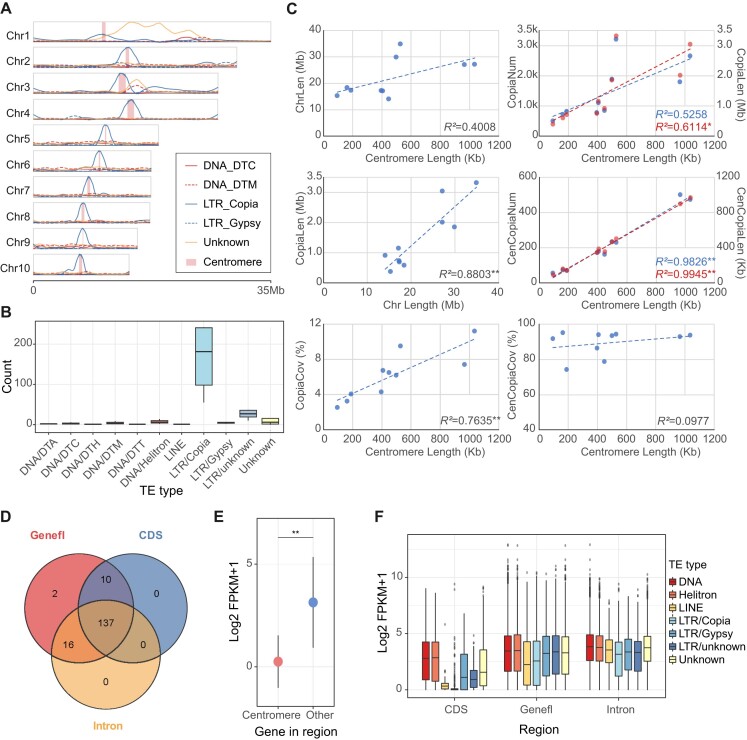
Relationship between centromere and chromosome characteristics. (A) Genome-wide fitting curve of TE coverage (windows: 500 kb). (B) Counts of each TE type in centromere regions. (C) Point plot and linear correlation analysis. *P < 0.05, **P < 0.01. Chr: chromosome. (D) TE insertion statistics in centromeric genes. (E) Comparison of expression among centromere genes and genes in other regions (*Wilcox* test, **P < 0.01). The circle represents the mean expression and the vertical line represents the standard deviation. (F) Relationship between TE insertion and expression at the genome-wide genes. Genefl: flanking region of genes.

To explore the correlation between the centromere and the evolution of chromosome length, we compared the composition of sequences on chromosomes. The distribution of transposons across the entire chromosome indicated a high proportion of *Copia* transposons significantly enriched in the centromeric region (>30% of the total length; chi-square test, *P* < 0.01) throughout the genome. Several complex regions exhibited high DNA transposon enrichment (Fig. 5A, B and Supplementary Fig. S7).

We further analyzed the quantity and length of *Copia* and the correlation between centromere length and chromosome length and revealed a correlation between centromere length and chromosome length (*R*^2^ = 0.4008), although this was not statistically significant (*P* = 0.1665). Subsequently, we found that chromosomes with longer centromeres had more and longer *Copia* elements (*R*^2^ = 0.6114, *P* < 0.05) and centromeres *Copia* (*R*^2^ = 0.9945, *P* < 0.01) (Fig. 5C). Therefore, we speculated that the enrichment of *Copia* might lead to a positive association between centromere and chromosome lengths in Sal_t2t, and this pattern might be diluted by the low density of *Copia* in the chromosome arms (Fig. 5C). Statistics and analysis of centromere sequences in lettuce and rice showed that the correlations among chromosome length, number and length of CRs, and centromere length were similar to those in sandalwood. However, in contrast to sandalwood, the coverage of CRs was negatively correlated with centromere length, indicating that the centromere composition of sandalwood was species specific and differed from that of the other species (Supplementary Fig. S8).

Given that TE insertion can influence gene expression, we also investigated TE insertion and expression levels in the centromeric and other regions. The results revealed that TE insertions occurred in all 165 centromeric genes in their 2-kb flanking regions, with 92.73% (153/165) of centromeric genes containing TEs in introns, and 89.09% (147/165) of genes overlapping between TEs and coding sequences (CDS) (Fig. 5D). These proportions were significantly higher than those of genes in other genomic regions (74.05%, 34.18%, and 5.65% of genes containing TEs in 2-kb flanking regions, introns, and CDS, respectively), and centromeric genes exhibited significantly lower expression compared to other genes in the genome (*Wilcox* test, *P* < 0.01) (Fig. 5E and Supplementary Fig. S9). Additionally, most of these genes could not be annotated using the NR and Swiss-Prot databases. Among the few genes identified, only partial fragments were functionally annotated (Supplementary Table S13). This indicates that these genes underwent rapid mutations, which may be related to the high density of *Copia* insertions.

Furthermore, we explored the effect of TE insertions on gene expression across all genes. We found that genes containing *Copia* insertions exhibited significantly lower expression levels than other types of TE insertions at the genome-wide level, with *Copia* insertions in the CDS regions resulting in the greatest reduction in gene expression (Fig. 5F). Importantly, we found that *Copia* insertion into the intronic regions significantly reduced gene expression in the sucker, a specialized organ of sandalwood, compared to expression in roots, whereas insertion into the gene flanking region was more strongly inhibited in the root, and there was no significant difference when inserted into the CDS region, suggesting a potential effect on organ differentiation (Supplementary Fig. S10).

## Discussion

Centromeres play a crucial role in maintaining genome stability in eukaryotes, shaping genome structure, and driving karyotype evolution [29, 30]. However, in plants, the evolutionary relationship between centromere structure and function remains unclear. This is because the centromeric sequences themselves do not encode proteins and lack evolutionary dependence despite their high diversity among closely related species and the highly conserved function of the centromere [31, 32]. In this study, we identified the sandalwood-specific centromere sequence composition and its impact on genome length and gene expression by assembling a sandalwood T2T genome. We compared the sequence compositions of different species in Santalales and found that although the centromere TR sequences were conserved, there were significant differences among the species, even in the case of conspecific hemiparasitism. Moreover, differentiation occurred even within the same LTR classification for the same species, as evidenced by differences in the CR and TR contents of these 3 species.

Three types of centromere sequence compositions have been reported in many species [30, 31]. For instance, in grapes, a 107-bp repeat sequence serves as a centromere signature sequence and is highly conserved among chromosomes. In addition, most TEs in plant centromeres are composed of LTR-type *Gypsy*-like retrotransposons [33, 34]. For example, in rice, *Gypsy* plays a pivotal role in the formation and evolution of the centromere, particularly in young *Gypsy* LTRs [35]. In cotton (*Gossypium hirsutum*), a similar situation was observed that unclassified LTRs and *Gypsy*-type LTRs were the primary components of centromeric regions, and *Gypsy* contributed to the centromere evolution compared to *Copia* [33]. In our study, we found that the type of centromere-specific LTR was mainly *Copia*, with a higher frequency distribution in the centromeres, similar to that in *Brassica oleracea* [36]. Moreover, the presence of *Copia* significantly reduced gene expression, and the insertion of *Copia* into CDS, introns, and flanking regions of genes significantly reduced gene expression in roots and suckers. For genes with *Copia* insertions in the intron, genes with lower expression in the sucker relative to the root were enriched for pathways such as biosynthesis and metabolism, suggesting that the expression of genes related to metabolic pathways was suppressed in the sucker, whereas basic nutrient uptake functions might be retained (Supplementary Tables S14 and S15). This might be an adaptive and survival strategy for sandalwood that reduces the production of unwanted secondary metabolites in the sucker, thus optimizing resource utilization for better uptake of nutrients from the host. Therefore, this particular *Copia* composition may be related to sandalwood parasitism.

In addition, some researchers have proposed that parasitism might lead to an increase in genome size [17, 37] because the parasitized plant is liberated from the limitations of the growth rate of the root meristematic tissue, or the resources obtained from the host. However, this was subsequently refuted in studies of the genus *Cuscuta*, which nonetheless found a correlation between genome size changes and the centromeric form, whereas no association was found between parasitic forms and genome size. For instance, species with monocentric chromosomes exhibit a 102-fold variation in genome size and a higher basic chromosome number, whereas species with holocentric chromosomes have modest genome sizes [17]. We also found that parasitism did not lead to genome expansion in sandalwood. The genome size of the nonparasitized *M. oleifera* was 1.5 Gb, whereas that of the hemiparasitic species *T. chinensis* was 521.90 Mb, and the genome size of hemiparasitic species *S. album* was 218.90 Mb. However, we found that *Copia* was positively correlated with the genome size. We found that chromosomes with longer centromeres exhibited more *Copia* and centromere-specific *Copia*. This suggests that *Copia* content promoted the length expansion of both centromeres and chromosomes.

In conclusion, we constructed the first T2T genome of sandalwood by combining HiFi, Hi-C, and ultra-long ONT data. We resolved the sequence composition and function of telomeres and centromeres and provided new insights into the genome evolution of parasitic plants.

## Methods

### Plant materials and genome sequencing

Genomic DNA was extracted from the leaves collected at the Experimental Station of the Research Institute of Tropical Forestry, Chinese Academy of Forestry, Guangzhou, China. The extracted DNA was assessed for concentration and quality using NanoDrop 2000 and used to construct linked read libraries using a PacBio SMRTbell library from an SMRTbell Prep Kit 3.0 (PN: 102–182-700), following the manufacturer’s protocols, and then sequenced on the PacBio Revio platform (PacBio Sequel II System, RRID:SCR_017990) for generating HiFi reads. For ONT ultra-long sequencing, a standard library was prepared using the SQK-LSK109 kit, following the standard protocol. The purified library was sequenced using a PromethION sequencer (Oxford Nanopore Technologies; RRID:SCR_017987). For RNA sequencing, phenol/chloroform was used to isolate RNA from root, sucker, stem, and leaf samples (3 bioreplicates), which were checked for purity and integrity before construction. Libraries for all 4 tissues were prepared using mRNA sequencing preparation kits and sequenced in PE150 mode on the MGISEQ-2000 platform.

### Genome assembly and quality evaluation

*De novo* assembly was performed using Hifiasm (v0.19.6-r595) (RRID:SCR_021069) [38]. For Hi-C sequence data [26], Juicer (v1.6) (RRID:SCR_017226) [39] and bowtie2 (v2.3.2) (RRID:SCR_016368) [40] were used to filter out low quality, and 3D-DNA (v180922) (3D *de novo* assembly, RRID:SCR_017227) [41] was used to unvalidated paired-end reads and construct interaction matrices to obtain chromosome-scale genomes. The redundant contigs were removed using Purge_Haplotigs (v1.1.2) (RRID:SCR_017616) [42]. The draft genome was then subjected to a final round of gap filling using ONT ultra-long reads corrected by NextDenovo (v2.17-r941) (RRID:SCR_025033) [43] with LR_Gapcloser (v1.9.4) (RRID:SCR_016194) [44] and Minimap2 (v2.24-r1122) (RRID:SCR_018550) [45] to obtain a T2T genome.

The completeness of the assembled Sal_t2t genome sequences was analyzed using BUSCO (v5.3.2) (RRID:SCR_015008) [46] with the embryophyta_odb10 databases (issued 2020-08-05, including 1,614 proteins), and the LAI statistic in LTR_retriever (v2.9.0) (RRID:SCR_017623) [47] with default parameters based on intermediate results from the EDTA pipeline (v1.9.4) (RRID:SCR_022063) [48]. To measure genome coverage based on read-mapping rates, NGS short reads [26], HiFi reads, and RNA sequencing reads were mapped against the assembled genome sequences using BWA-MEM (v0.7.9a, [49]) (RRID:SCR_022192), minimap2, and HISAT2 (v2.2.1) (RRID:SCR_015530) [50]. The GC content distribution was used to detect sample contamination.

### Genome annotation

To identify repeat sequences in Sal_t2t and the other 3 Santalales species, several programs in EDTA, including LTR_FINDER (v1.07) (RRID:SCR_015247), LTRharvest (genometools, v1.6.1) (RRID:SCR_018970), LTR_retriever, Generic Repeat Finder (v1.0), HelitronScanner (v1.1), TIR-Learner (v2.5), RepeatMasker (v4.1.1) (RRID:SCR_012954), and RepeatModeler (v2.0.1) (RRID:SCR_015027), as well as a series of integration scripts, were used to annotate and identify LTR, LINE, SINE, Helitron, MITE, and other retrotransposon and transposon sequences, with options “–anno 1 –force 1 –debug 1 -sensitive 1.” In addition, we utilized Tandem Repeat Finder (TRF, v4.09) (RRID:SCR_022193) [51] with the parameters “2 7 7 80 10 50 500 -f -d -m” to independently predict tandem repeats in the genome.

To annotate the gene structure, we used the GETA pipeline (v2.5.1, [52]) with 3 methods: homology, *de novo*, and transcript-based annotation. Published protein information for *Vitis vinifera, Arabidopsis thaliana, M. oleifera, S. yasi*, and V1 genomes was used as a homology reference. The parameters “[hisat2] –min-intronlen 20 –max-intronlen 20000 –dta –score-min L,0.0,-0.4, [sam2transfrag] –fraction 0.05 –min_expressed_base_depth 2 –max_expressed_base_depth 50 –min_junction_depth 2 –max_junction_depth 50 –min_fragment_count_per_transfrags 10 –min_intron_length 20, [TransDecoder.LongOrfs] -m 100 -G universal, [homolog_genewise] –coverage_ratio 0.4 –evalue 1e-9, [homolog_genewiseGFF2GFF3] –min_score 15 –gene_prefix genewise –filterMiddleStopCodon, [geneModels2AugusutsTrainingInput] –min_evalue 1e-9 –min_identity 0.8 –min_coverage_ratio 0.8 –min_cds_num 2 –min_cds_length 450 –min_cds_exon_ratio 0.60, [prepareAugusutusHints] –margin 20, [paraAugusutusWithHints] –gene_prefix augustus –min_intron_len 20, [paraCombineGeneModels] –overlap 30 –min_augustus_transcriptSupport_percentage 10.0 –min_augustus_intronSupport_number 1 –min_augustus_intronSupport_ratio 0.01, [pickout_better_geneModels_from_evidence] –overlap_ratio 0.2 –ratio1 2 –ratio2 1.5 –ratio3 0.85 –ratio4 0.85, [PfamValidateABinitio] –CDS_length 750 –CDS_num 2 –evalue 1e-5 –coverage 0.25, [remove_genes_in_repeats] –ratio 0.8” were used in GETA pipline.

Finally, we used CMScan (v1.1.4, [53]) to mine noncoding RNA (ncRNA) information using the Rfam nonredundant database, which is based on the homology annotation of ncRNAs, including tRNA, rRNA, microRNA, and snRNA. These results were visualized using the Integrative Genomics Viewer (IGV, v.2.12.3) (RRID:SCR_011793) (Supplementary Fig. S11).

### Genome comparison

V1 assembled genome and the *M. oleifera* genome were downloaded from the CNCB under accession number PRJCA009490 and PRJNA472200. V2 assembled genome and annotation were downloaded from the Figshare database [54]. *T. chinensis* genome was downloaded from NCBI under PRJNA855314.

The Synteny and Rearrangement Identifier (SyRI v1.5.4) (RRID:SCR_023008) [55] was utilized to detect collinearity, SVs, and PAVs among the 3 versions of the sandalwood genomes. ONT ultra-long and HiFi reads were used to validate the accuracy of the assembly of Sal_t2t with read mapping.

### Identification of new genes

We used Liftoff (v1.6.3) [56] with default options to identify new genes. The homologous annotation results were compared with annotation files using the BEDtools (v2.30.0) (RRID:SCR_006646) [57] and the AWK command. Genes that were newly annotated in the homologous annotations but not in the annotation files were considered new results because of the differences in software use. The genes in the unmapped files were new genes.

### Organelle genome assembly

GSAT (v1.11) [58] was used to assemble the mitochondrial genome with 4 Gb Illumina reads and all HiFi reads. SPAdes (v3.15.5) (RRID:SCR_000131) [59] was used to assemble the chloroplast genome with 5 Gb Illumina reads. The complete CP and MT assemblies were visualized with Bandage (v0.9.0) (RRID:SCR_022772) [60] to remove contigs with abnormal coverage and simplify the genome using the organelle genomes downloaded from NCBI (NC_081498.1 and NC_048953.1) as a reference.

### Organelle gene transfer

Based on the assembly and annotation files of the *S. album* nuclear genome, Blastn (v0.8.1) (RRID:SCR_001598) software was used to identify transfer events from organelle to nuclear genomes with default parameters. We filtered the transfer fragments less than 30 bp in length and the identify score less than 80%. We extracted the 500 bp, 1,000 bp, and 2,000 bp upstream and downstream of the transferred fragments for TE statistics combined with the EDTA annotation results. These results were visualized using the ggplot2 package (RRID:SCR_014601) in R [61].

### Identification of telomeres and centromeres

Referring to the research methods used in the grape (PN40024) genome [9], we used TIDK (v.0.2.0) [62], TRF for the identification of centromeres and telomeres, and, combined with the result from quarTeT (v1.1.4) (RRID:SCR_025258) [63], the EDTA pipeline and srf [64] as a complement. The telomere repeat units were explored by TIDK with options “tidk explore -f genome.fa -minimum 5 -maximum 12 -o tidk_explore -t 2 -log -dir telomere_find -extension TSV.” Then, the whole genome was searched using the following parameters: “tidk search -f genome.fa -s AAACCCT -o tidk_search -dir telomere_find.”

For centromere annotation, we used the candidate regions identified by quarTeT and the blank regions in Hi-C contact matrices referring to the method for the faba genome [65], extracted all TE sequences presented in the candidate centromeric regions, and calculated the length and count of these TEs. We selected the top 10 TEs and found that 3 *Copia* repeats (TE_00001095, TE_00001228, and TE_00001258) and 1 unknown LTR repeat (TE_00000831) were mainly enriched in the candidate regions. TRF was used to scan tandem repeats ranging from 30 to 500 bp in the genome, and then we merged the results of annotation using trf2gff in TRF. We visualized the top 30 repetitive sequences in terms of total count and length using the ggplot2 package in R but could not find the enrichment of tandem repeats in most centromeres. We then used SRF to identify HORs and found only 1 HOR located on all chromosomes. Combined with the TRF results, this HOR was found to be composed of two 20-bp tandem repeat sequences and was considered a characteristic of centromeric satellites. To complete the data statistics and visualisation, we used information from the TRF, SRF, and EDTA results extracted by the AWK command in the Linux system and analyzed the results using the IGV. Fitting and visualization were implemented using the ggplot2 package in R. We considered the intersection region of the TRs (from SRF) and TEs (from the EDTA pipeline and TIDK) to be the centromere region.

Candidate centromeres in *M. oleifera, T. chinensis*, V2, rice, and lettuce [66] were identified using the method described above (Supplementary Tables S16–S18).

### Evolution analysis of CRs in Santalales

We used BEDTools to extract the CRs and aligned using MAFFT (v7.480) (RRID:SCR_011811) [67]. Then we used BMGE (v1.12) [68] to remove ambiguously aligned regions with options “-g 0.85 -h 1 -b 1 -w 1.” We constructed an maximum likelihood (ML) tree using FastTree (v2.1.10) (RRID:SCR_015501) [69] with a GTR model. Finally, the tree was adjusted, customized, and displayed using iTOL (RRID:SCR_018174) [70].

## Additional Files

**Supplementary Fig. S1**. Structural variation between 3 Santalales assemblies.

**Supplementary Fig. S2**. GO enrichments of new genes and disappeared genes in the Sal_t2t genome.

**Supplementary Fig. S3**. Density distribution of transfer fragment length of 3 sandalwood assemblies. Cp: chloroplast genome; Mt: mitochondrial genome.

**Supplementary Fig. S4**. Counts of TE insertions in different flanking regions (bp) of transfer fragments.

**Supplementary Fig. S5**. Counts of different types of TE insertions in different flanking regions (bp) of transfer fragments.

**Supplementary Fig. S6**. Distribution of top 20 TRs in each chromosome.

**Supplementary Fig. S7**. Count distribution of different type of TEs in each chromosome.

**Supplementary Fig. S8**. Point plot and linear correlation analysis of rice and lettuce. *P < 0.05, **P < 0.01.

**Supplementary Fig. S9**. TE insertion statistics in all genes. CDS: coding sequence; Genefl: flanking region of genes; Intron: intronic region.

**Supplementary Fig. S10**. Relationship between TE insertion and expression in root and sucker. The vertical line represents the standard error. CDS: coding sequence; Genefl: flanking region of genes; Intron: intronic region.

**Supplementary Fig. S11**. Annotations of complex regions were visualized by IGV, and the distribution of genes, TEs (excluding Chr1), all TEs, TRs, 5S rRNAs, and rRNAs other than 5S are shown from top to bottom.

## Abbreviations

BUSCO: Benchmarking Universal Single-Copy Orthologs; bp: base pairs; CDS: coding sequences; CRs: centromeric retrotransposons; Gb: gigabase pairs; Hi-C: high-throughput chromosome conformation capture; HORs: high-order repeats; IGR: intergenic region; IGT: intracellular gene transfer; Kb: kilobase pairs; LAI: LTR assembly index; LTRs: long terminal repeats; Mb: megabase pairs; miRNA: microRNA; ML: maximum likelihood; NCBI: National Center for Biotechnology Information; ncRNA: noncoding RNA; NUMT: nuclear integrant of mitochondrial DNA; NUPT: nuclear integrant of plastid DNA; ONT: Oxford Nanopore Technologies; PacBio: Pacific Biosciences; PAV: presence/absence variation; QV: quality value; rRNA: ribosomal RNA; SMRT: single-molecule real-time sequencing; snRNA: small nuclear RNA; SV: structural variation; T2T: telomere-to-telomere; TE: transposable element; TR: tandem repeat; tRNA: transfer RNA.

## Supplementary Material

giae096_GIGA-D-24-00225_Original_Submission

giae096_GIGA-D-24-00225_Revision_1

giae096_Response_to_Reviewer_Comments_Original_Submission

giae096_Reviewer_1_Report_Original_Submission Xupo Ding -- 7/16/2024 Reviewed

giae096_Reviewer_1_Report_Revision_1 Xupo Ding -- 9/29/2024 Reviewed

giae096_Reviewer_2_Report_Original_Submission Chao Bian, ph.D -- 7/24/2024 Reviewed

giae096_Reviewer_2_Report_Revision_1 Chao Bian, ph.D -- 9/23/2024 Reviewed

giae096_Supplemental_Files

## Data Availability

The nuclear genome assembly and all sequencing data, including HiFi, ONT, and RNA-seq, have been submitted to the NCBI under BioProject number PRJNA1127301. Gene annotations, chloroplast genome, and mitochondrial genome have been deposited to Figshare [71]. All supporting data and materials are available in the *GigaScience* database, GigaDB [72].
