# Supplementary material for: The telomere-to-telomere (T2T) genome provides insights into the evolution of specialized centromere sequences in sandalwood
Source: Gigascience. 2024 Dec 11;13:giae096. doi: 10.1093/gigascience/giae096 (PMC11633456; doi:10.1093/gigascience/giae096)
Supplement: giae096_GIGA-D-24-00225_Original_Submission [file giae096_giga-d-24-00225_original_submission.pdf]

GigaScience

# The telomere-to-telomere (T2T) genome provides insights into the evolution of specialized centromere sequences in sandalwood

--Manuscript Draft--

| | |
|---|---|
| Manuscript Number: | GIGA-D-24-00225 |
| Full Title: | The telomere-to-telomere (T2T) genome provides insights into the evolution of specialized centromere sequences in sandalwood |
| Article Type: | Research |
| Funding Information: | Guangdong Pearl River Talent Program (2021QN02N792) — Dr Zhiqiang Wu<br>Chinese Academy of Agricultural Sciences Elite Youth Program (110243160001007) — Dr Zhiqiang Wu |
| Abstract: | Background<br><br>Sandalwood, a prized hemiparasitic plant, is highly sought after for its aromatic core materials in the commercial market. The structure and stability of its genome are instrumental in the rapid adaptation of parasitic plants to their surroundings. Nevertheless, there is a conspicuous lack of research dedicated to the genomic-level adaptive evolution of Sandalwood.<br><br>Results<br><br>In this study, we assembled a gap-free telomere-to-telomere (T2T) reference genome for Santalum album, utilizing PacBio HiFi, Hi-C, and ultra-long ONT data. This T2T reference genome (Sal_t2t) encompassed annotations for 24,171 genes and 25.34% repetitive sequences, in addition to all ten centromeres and twenty telomeres across the ten chromosomes. Our research revealed that three distinct parasitic species of Santalales showed diverse centromere compositions. The Copia-type LTR transposon emerged as the most significant in the S. album genome, constituting the primary sequence of the centromere and exerting influence on gene expression. In sandalwood, the presence of Copia impacted the size of the centromeres, and consequently, the genome size. The unveiling of the sandalwood T2T genome in this study has also enabled the identification of more precise organelle transfer fragments.<br><br>Conclusions<br><br>Our research provided a sandalwood T2T genome, laying the groundwork for future investigations into the evolution of energy organs in parasitic plants. Moreover, it offered novel insights into studying the function and evolution of centromeres, as well as mechanisms of adaptation and parasitism. |
| Corresponding Author: | Zhiqiang Wu<br>Chinese Academy of Agricultural Sciences Agricultural Genomes Institute at Shenzhen<br>Shenzhen, Guangdong CHINA |
| Corresponding Author Secondary Information: | |
| Corresponding Author's Institution: | Chinese Academy of Agricultural Sciences Agricultural Genomes Institute at Shenzhen |
| Corresponding Author's Secondary Institution: | |
| First Author: | Dan Peng |
| First Author Secondary Information: | |
| Order of Authors: | Dan Peng |
| | Zhou Hong |
| | Shenglong Kan |

Powered by Editorial Manager® and ProduXion Manager® from Aries Systems Corporation

| | |
|---|---|
| | Zhiqiang Wu |
| | Xuezhu Liao |
| **Order of Authors Secondary Information:** | |
| **Additional Information:** | |
| **Question** | **Response** |
| Are you submitting this manuscript to a special series or article collection? | No |
| **Experimental design and statistics**<br><br>Full details of the experimental design and statistical methods used should be given in the Methods section, as detailed in our Minimum Standards Reporting Checklist. Information essential to interpreting the data presented should be made available in the figure legends.<br><br>Have you included all the information requested in your manuscript? | Yes |
| **Resources**<br><br>A description of all resources used, including antibodies, cell lines, animals and software tools, with enough information to allow them to be uniquely identified, should be included in the Methods section. Authors are strongly encouraged to cite Research Resource Identifiers (RRIDs) for antibodies, model organisms and tools, where possible.<br><br>Have you included the information requested as detailed in our Minimum Standards Reporting Checklist? | Yes |
| **Availability of data and materials**<br><br>All datasets and code on which the conclusions of the paper rely must be either included in your submission or deposited in publicly available repositories (where available and ethically appropriate), referencing such data using | Yes |

| | |
|---|---|
| a unique identifier in the references and in the “Availability of Data and Materials” section of your manuscript.<br><br>Have you have met the above requirement as detailed in our Minimum Standards Reporting Checklist? | |

Powered by Editorial Manager® and ProduXion Manager® from Aries Systems Corporation

Manuscript Click here to access/download;Manuscript;manuscript.docx

# The telomere-to-telomere (T2T) genome provides insights into the evolution of specialized centromere sequences in sandalwood

Dan Peng[1,2], Zhou Hong[3], Shenglong Kan[4], Zhiqiang Wu[1], Xuezhu Liao[1]

1. Shenzhen Branch, Guangdong Laboratory for Lingnan Modern Agriculture, Genome Analysis Laboratory of the Ministry of Agriculture, Agricultural Genomics Institute at Shenzhen, 518120, Shenzhen, China.
2. Center for Genomics and Biotechnology, Haixia Institute of Science and Technology, Fujian Agriculture and Forestry University, 350002, Fuzhou, China.
3. Research Institute of Tropical Forestry, Chinese Academy of Forestry, 510520, Guangzhou, China.
4. Marine College, Shandong University, 264209, Weihai, China

These authors contributed equally: Dan Peng, Zhou Hong.

Corresponding author: Xuezhu Liao (liaoxuezhu@caas.cn), Zhiqiang Wu (wuzhiqiang@caas.cn).

## Abstract

**Background**

Sandalwood, a prized hemiparasitic plant, is highly sought after for its aromatic core materials in the commercial market. The structure and stability of its genome are instrumental in the rapid adaptation of parasitic plants to their surroundings. Nevertheless, there is a conspicuous lack of research dedicated to the genomic-level adaptive evolution of Sandalwood.

**Results**

In this study, we assembled a gap-free telomere-to-telomere (T2T) reference genome for *Santalum album*, utilizing PacBio HiFi, Hi-C, and ultra-long ONT data. This T2T reference genome (Sal_t2t) encompassed annotations for 24,171 genes and 25.34% repetitive sequences, in addition to all ten centromeres and twenty telomeres across the ten chromosomes. Our research revealed that three distinct parasitic species of Santalales showed diverse centromere compositions. The *Copia*-type LTR transposon emerged as the most significant in the *S. album* genome, constituting the primary sequence of the centromere and exerting influence on gene expression. In sandalwood, the presence of *Copia* impacted the size of the centromeres, and consequently, the genome size. The unveiling of the sandalwood T2T genome in this study has also enabled the identification of more precise organelle transfer fragments.

**Conclusions**

Our research provided a sandalwood T2T genome, laying the groundwork for future investigations into the evolution of energy organs in parasitic plants. Moreover, it

offered novel insights into studying the function and evolution of centromeres, as well as mechanisms of adaptation and parasitism.

## Keywords

T2T genome; Centromere; Hemiparasitic species; Cyto-nuclear transfer

## Introduction

With the advent of long read-length sequencing technologies and improved algorithms, genome assembly has entered a new era: telomere-to-telomere (T2T) assembly [1-3]. Compared to the genome with gaps, T2T genomes consist of minimal or no unassembled regions. They contain more comprehensive and complete information on telomeres, centromeres, rDNA, complex chromosomal regions, and intracellular gene transfer (IGT) [4]. These areas are often challenging to handle, and complete assemblies may offer possibilities for a deeper understanding of its structure and function. The T2T genome of *Arabidopsis thaliana* reported in 2021 facilitated the exploration of its centromeric genetic and epigenetic characteristics, and unveiled the mechanism of centromere evolution driven by the homologation of satellite sequences and retrotransposons, and is considered to have marked the first application of the T2T assembly technology in plants [5]. Since then, the sequence composition and evolution of centromeres have been elucidated by T2T genomes in several species, including rice, kiwifruit, watermelon, grape, carnation, and *Peucedanum praeruptorum*, among others [6-11]. However, the number of T2T genomes published to date remains limited.

The centromere, a vital structure of eukaryotic chromosomes, plays an indispensable role in cell division [12]. Dysfunctions in the centromere often lead to incorrect chromosome segregation during cell division, which can impact the growth and development of organisms [13]. For instance, in plants, abnormal centromere function could result in stunted growth and development [14]. The structural and

functional elucidation of the centromere is not only a fundamental scientific issue in the field of chromosome biology but also a cornerstone for the advancement of synthetic biology. Studies on the composition, structure, and evolution of centromere sequences are key to unraveling their function. However, the high degree of repetitive sequences in the centromere presents a challenge to their precise assembly and functional resolution. Generally, plant centromeric DNA sequences comprise three types: tandem repeats (TR), centromeric retrotransposons (CR), and a few functional genes with transcriptional activity [9, 15, 16]. CRs are typically interspersed with TRs and are abundant in plant centromeric regions, which are depicted as blank regions in Hi-C contact heatmaps. Therefore, the T2T genome can provide a more accurate sequence foundation for identifying these signature sequences. However, most studies have been confined to crops or widely recognized horticultural plants, with few reports on the sequence characteristics of the centromeres of certain plant taxa, such as parasitic plants. In contrast, for parasitic plants, maintaining chromosome stability is essential for their survival and reproduction, as they may need to rapidly adapt to changes in the host plant or environment. For instance, in the parasitic genus *Cuscuta*, the form of the centromeres is believed to be associated with its genome size and chromosome base, with monocentric *Cuscuta* species having 102-fold variation in genome sizes and holocentric species having moderately sized genomes [17]. Moreover, to accommodate its parasitic lifestyle, the parasitic plant's organelles may have undergone specific adaptive modifications, particularly in cytonuclear interactions or transfers [18]. Therefore, we need the complete genomes of parasitic

plants for relevant studies.

*Santalum album*, or sandalwood, is a precious hemiparasitic plant in the Santalaceae. Sandalwood is an evergreen tree found across Southeast Asia, Australia, and Pacific islands, known for its medicinal properties like antimicrobial, antioxidant, and anti-inflammatory effects, and it's commercially prized for its aromatic core materials [19, 20]. Its essential oil, termed "liquid gold", is used in perfumes, cosmetics, and incense. The hard texture and beautiful grain also make sandalwood ideal for carving and furniture[21]. Interestingly, the Santalales not only encompasses hemiparasitic plants like sandalwood but also includes non-parasitic (*Malania oleifera*), holoparasitic (*Balanophora subcupularis*), and other hemiparasitic species (*Taxillus chinensis*) [22-24]. Parasitic plants typically display greater genomic sequence and structural differences compared to non-parasitic plants as an adaptation to their environment [25]. Previous studies have reported the assembly of two chromosome/contig-level genomes with dozens to hundreds of gaps for sandalwood [26, 27]. Thus, there remains a certain research gap in the sequence differences between parasitic plants and non-parasitic plants in Santalales. Therefore, it is imperative to obtain a high-quality T2T genome to further investigate additional unknown information in sandalwood.

In this study, we successfully obtained the T2T genome of *S. album*, with a size of 218.90 Mb, comprising 10 chromosomes and no gaps. We identified all centromere and telomere sequences across chromosomes. Our findings revealed that three species in Santalales with different parasitic forms exhibited differences in centromere

composition and that *Copia*-type LTRs significantly influenced *S. album* gene expression. In summary, this study represents the first T2T assembly of the *S. album* genome, and provides an opportunity to delve into the genome structure and function of Santalales species.

## Result

### The gap-free genome assembly, completeness evaluation and annotation for *S. album*

By integrating 19.91 Gb (~90 x coverage) of Hifi reads, 98.13 Gb (~430 x coverage) of Hi-C data, and 35.02 Gb (~160 x coverage) of ultra-long ONT reads, we obtained a gap-free sandalwood genome (named Sal_t2t) with a size of 218.90 Mb, comprising 10 chromosomes. The longest chromosome measures 34.97 Mb, and the shortest is 14.10 Mb (Fig. 1A, Table 1 and Supplementary Table S1). Compared to two previously published versions of the sandalwood genome (V1: 23 gaps; V2: 108 gaps), all gaps in Sal_t2t were filled. Additionally, all SVs and PAVs, which differ from the previous two assemblies in complex regions or diversities between haplotypes, were supported by more than 3 long reads (Hifi or ultra-long ONT reads) in the Sal_t2t genome (Fig. 1 C-E, Supplementary Fig. S1). The contig N50 length of the Sal_t2t genome (18.40 Mb) is 1.44-3.31 times greater than that of V1 (12.75 Mb) and V2 (5.56 Mb) genomes, demonstrating a significant improvement in continuity and completeness in the newly assembled Sal_t2t (Table 1).

We further assessed the accuracy of the assembly through several means. Firstly, the BUSCO assessment based on embryophyta_odb10 revealed that 98.3% of the core

conserved plant genes (1,586/1,614 genes) were fully characterized in this genome. Secondly, the comparison showed that 99.65% of short reads, 99.97% of Hifi reads, and 96.12% of RNA-Seq data from leaf samples could be mapped to the Sal_t2t genome. Furthermore, integrity checks of the long terminal repeats (LTRs) showed an assembled LTR assembly index (LAI) of 26.41. Finally, this genome showed a consensus quality value (QV) of 67.30. Collectively, these data demonstrate the high accuracy of Sal_t2t assembly (Table 1). After that, a total of 567 tRNA, 332 snRNA, and 3,910 rRNA sequences were annotated and predicted in the sandalwood genome based on the Rfam database (Table 1 and Supplementary Table S4). Among these, 3,324 were annotated as 5S rRNA, accounting for 85.01% of the total rRNA count, primarily distributed in the genomic region of 21.72-23.38 Mb on Chr1.

The Sal_t2t genome provided an unprecedented opportunity to identify all repeat sequences and genes. Consistent with previous predictions, 25.34% (55.48 Mb) of the sequences were identified as transposable elements (TEs), with 19.54 Mb (8.93%) of retrotransposons and 23.04 Mb (10.51%) of DNA transposons in Sal_t2t (Fig. 1F and Supplementary Table S2). Both retrotransposons and DNA transposons were predominantly distributed in the central regions of chromosomes (Fig. 1B). We further predicted 24,171 protein-coding genes, and 12 of which were pseudogenes or incomplete (Tables 1 and Supplementary Table S3). Moreover, the completeness of the annotations was assessed by BUSCO, revealing that about 96.0% of the core genes in the gene set were complete, a result much higher than those of the V1 (93.5 %) and V2 (89.6 %) versions (Fig. 1G and Tables 1).

**Organelle gene transfer**

The integrity of identification in organelle transfer fragments, such as nuclear integrants of mitochondrial DNA (NUMTs) and nuclear integrants of plastid DNA (NUPTs), also serves as an index to evaluate assembly quality [28]. By assembling the nuclear, mitochondrial, and chloroplast genomes of *S. album*, we conducted an evaluation of the frequency and patterns of NUPTs and NUMTs among Sal_t2t, V1, and V2 genomes and found similar quantity and length distribution patterns. However, more accurate organelle transfer fragments can be identified in Sal_t2t than in the other two versions, especially in the intergenic region (IGR) (Fig. 2A, B and Table S5).

In comparison with the ultra-long ONT sequence, we also identified a mis-assembly of the nuclear genome involving five NUPTs larger than 10 Kb, which was corrected in Sal_t2t. Additionally, we observed that regions where the same chloroplast fragment transferred multiple times to the nuclear genome might also be mis-assembled, including redundancy in low-copy chloroplast transfer fragments, partial or complete loss in assembling multi-copy chloroplast transfer fragments (Supplementary Table S6, S7). Sequence alignment verification results also demonstrated the integrity and accuracy of transfer fragment assembly in the T2T genome (Fig. 2C-F).

We also examined the quantity of transposons in upstream and downstream of organelle transfer fragments and found that the V2 and V1 genomes had the fewer transposable elements (TEs) surrounding organelle transfer fragments, while Sal_t2t

had the higher intact TEs (Fig. 2G and Supplementary Fig. S3). Further categorization revealed that the quantities of LTR, MITE, TIR, LINE, and Helitron transposons were similar among the three versions, but with significant differences in DNA transposons and unknown transposons (Supplementary Fig. S4). In DNA transposons, the average content was highest in Sal_t2t and lowest in V2, while the trend was reversed in unknown transposons (Fig. 2H). It indicated that the Sal_t2t genome provided more clearly and accurately prediction of the distribution, types and quantities of TEs surrounding transfer fragments.

**Architecture and context of telomeres and centromeres**

The completion and accuracy of the genome have enabled us to identify telomeres and centromeres. Firstly, the results of telomeric regions revealed that both ends of 10 chromosomes possessed telomere repeat units (AAACCCT/AGGGTTT) in the sandalwood, aligning with the telomere structure of most plants. The longest telomere, located on chromosome 1, measured 16.62 Kb and contained 2,374 repeats, while the shortest telomere, found on chromosome 9, measured 1,869 bp with only 267 repeats (Supplementary Tables S8).

In addition, we identified centromeric regions by quarTeT, combined with EDTA annotations and the blank regions in Hi-C contact matrices as candidate centromeric regions (Fig. 3A and B). We quantified and visualized TEs in each chromosome's candidate regions and observed that three *Copia* repeats (TE_00001095, TE_00001228, TE_00001258) and one unknown LTR repeat (TE_00000831) were highly enriched in eight candidate regions and two secondary candidate regions across

chromosomes, while they were scarce on chromosome arms (Fig. 3C). We speculated that these four LTR sequences were CRs.

Secondly, we identified 470 distinct TR units in the Sal_t2t genome. A 312 bp repeat was the most abundant unit in the genome, with a total of 10,335 copies of ⩾2 repeats, accounting for 1.47% of the entire genome sequence. This was followed by 500 bp (0.72%), 32 bp (0.42%), and 63 bp (0.30%). However, the top 20 units in the genome in terms of total length and total copy number lacked the typical centromere TR sequence distribution characteristics (Supplementary Fig. S5). Subsequently, 21 types of high-order repeats (HORs) regions were identified, among which the SR3 (prefix#circ3-7198) was primarily composed of two tandem repeats of 20 base pairs in length (AGCCCAAGCACACTTGGAGG and TCCAAGTGTCATTGGGCTCC), which was highly overlapped with the candidate regions and CRs (Fig. 2D-E and Supplementary Table S9). Therefore, we defined the distribution range of CR and TR (from SRF results) as the centromeric regions of all the chromosomes (Supplementary Tables S10).

**Comparative analysis of centromeric sequences in Santalales**

In order to study the differences and evolution of centromere sequences among various species of Santalales, we identified and analyzed the centromere sequences of available genomes of Santalales species. We focused particularly on the CRs and TRs of *S. album*, *M. oleifera*, and *T. chinensis*. The genomes of *Balanophora* and *Scurrula* were excluded from the analysis due to the low quality of their assemblies.

In the Sal_t2t genome, TRs consisted of two sequences of 20 base pairs (bp), a

pattern similarly observed in the V2 genome. These 20 bp sequences were found in low numbers in the centromeric region, with only 2 copies per repeat unit. The number of repeat units per chromosome ranged from 2 (Chr9) to 46 (Chr4), averaging 19.5 units per chromosome. We identified TRs in the same pipeline for the other two species and found the length (259 bp and 260 bp) and copy number (101 - 223) of TR repeat units in *T. chinensis* were significantly higher compared to the sandalwood genome. In *M. oleifera*, centromeric tandem repeats primarily consisted of 66 bp repeat sequences, with copy numbers ranging from 10 to 110 (Fig. 4A).

In terms of centromeric retrotransposons (CRs) sequences, we identified three *Copia* sequences and one unknown LTR sequence in Sal_t2t, with a quarter of the TE_00001095 sequence being intact LTRs. However, in *T. chinensis*, we only identified one unknown LTR sequence, while in *M. oleifera*, we identified one *Copia* sequence and one *Gypsy* sequence respectively. We utilized these CRs to construct phylogenetic trees and observed the LTR sequence TE_00001228 of sandalwood clustered with several LTR sequences of *T. chinensis* and *Copia* sequences of *M. oleifera* in the same clade, whereas the LTR sequence TE_00000831 of sandalwood belonged to the same clade as the majority of LTR sequences of *T. chinensis* (Fig. 4B). Additionally, the complete LTR sequence TE_00001095 of sandalwood did not cluster with *T. chinensis* or *M. oleifera*, while the majority of *Copia* sequences and *Gypsy* sequences of *M. oleifera* clustered separately into distinct clades. Furthermore, differentiation has occurred in the same LTR classification in the same species, as evidenced by the difference in content of CR and TR in these four species (Fig. 4C).

These findings suggested that while centromere TR and CR sequences were conserved in species, they differed significantly between species. However, due to the limitations in the quantity and quality of published genome assemblies, further analysis and comparison of telomeric regions might require additional T2T genome sequences from the same family or genus. This could be particularly relevant for assessing the conservation of CR and TR sequences at different taxonomic levels.

**Relationship between centromere and chromosome characteristics**

In an effort to explore the correlation between centromere and the evolution of chromosome length, we compared the composition of sequences on chromosomes. The distribution of transposons across the entire chromosome indicated a high proportion of *Copia* transposons significantly enriched in the centromeric region (>30% of the total length, *chi*-test, $p < 0.01$) throughout the genome. While several complex regions exhibited a high proportion of DNA transposon enrichment (Fig. 5A, B and Supplementary Fig. S6).

We further analyzed the quantity and length of *Copia* and the correlation between centromere length and chromosome length and revealed a correlation between centromere length and chromosome length ($R^2$ = 0.4008), although it is not statistically significant ($p$ = 0.1665). Subsequently, we found that chromosomes with longer centromeres have more and longer *Copia* elements ($R^2 = 0.6114$, $p < 0.05$) and centromere *Copia* ($R^2 = 0.9945$, $p < 0.01$) (Fig. 5C). Therefore, we speculated that the enrichment of *Copia* may lead to a positive association between centromere length and chromosome length in Sal_t2t and this pattern might be diluted by the low-

density of *Copia* in chromosome arms (Fig. 5C).

Given that transposable element (TE) insertion can influence gene expression, we also investigated the TE insertion and expression level between centromeric regions and other regions. The results revealed that TE insertions occurred in all 165 centromeric genes in their 2 Kb flanking regions, with 92.73% (153/165) of centromeric genes containing TEs in introns, and 89.09% (147/165) of genes overlapping between TEs and coding sequences (CDS) (Fig. 5D). These proportions were significantly higher than those of genes in other genomic regions (74.05%, 34.18% and 5.65% of genes containing TEs in 2 Kb flanking regions, introns and CDS, respectively), and centromeric genes exhibited significantly lower expression compared to other genes in the genome (*wilcox* test, $p < 0.01$) (Fig. 5E and Supplementary Fig. S7). Additionally, most of these genes could not be annotated in NR and Swiss-Prot databases. In few identified genes, only partial fragments have received functional annotation (Supplementary Table S11). This indicated that these genes have undergone rapid mutations, which may be related to the high density of *Copia* insertions.

Furthermore, we explored the impact of TE insertions on gene expression across all genes. We found genes containing *Copia* insertions exhibited significantly lower expression levels compared to other types of TE insertions at the genome-wide level, with *Copia* insertions in CDS regions resulting in the greatest reduction in gene expression (Fig. 5F). Importantly, we found that *Copia* insertion into the intronic regions significantly reduced gene expression in sucker, a specialized organ of

sandalwood, compared to expression in roots, while the insertion into the gene flanking region were more strongly inhibited in the root, and there was no significant difference when inserted into the CDS region, which suggested a potential effect on organ differentiation (Supplementary Fig. S8).

## Discussion

Centromeres play a crucial role in maintaining genome stability in eukaryotes, where they shape the structure of the genome and drive the evolution of the karyotype [29, 30]. However, in plants, the evolutionary relationship between centromere structure and function remains elusive. This is because the centromere sequences themselves do not encode proteins and lack evolutionary dependence, despite their high diversity among closely related species, and the highly conserved function of centromere [31, 32]. In this study, we identified the sandalwood-specific centromere sequence composition and its impact on genome length and gene expression by assembling the sandalwood T2T genome. We compared the sequence compositions of different species in Santalales, and found although the centromere TR sequences were conserved in the species, there were significant differences among the species, even in the case of conspecific hemiparasitism. Moreover, differentiation occurred even in the same LTR classification of the same species, as evidenced by the differences in CR and TR contents of these three species.

Three type of centromere sequence compositions have been reported in many species [30, 31]. For instance, in grape, a 107 bp repeat sequence served as a centromere signature sequence and was highly conserved between chromosomes [33].

Additionally, most of the TEs of plant centromeres were composed of LTR-type *Gypsy*-like retrotransposons [34, 35]. For example, in rice, *Gypsy* played a pivotal role in the formation and evolution of the centromere, especially the young *Gypsy* LTRs [36]. In cotton (*Gossypium hirsutum*), a similar situation was observed that unclassified LTRs and *Gypsy*-type LTRs were the primary components of centromeric regions, and *Gypsy* contributed to the centromere evolution compared to *Copia* [34]. In our study, we found that the type of centromere-specific LTR is mainly *Copia*, with a higher frequency distribution in the centromeres, which was similar to that in *Brassica oleracea* [37]. Moreover, the presence of *Copia* significantly reduced gene expression, and the insertion of *Copia* into CDS, intron and flanking regions of genes significantly reduced gene expression in root and sucker. For genes with *Copia* insertions in the intron, genes with lower expression in the sucker relative to the root were enriched for pathways such as biosynthesis and metabolism, suggesting that expression of genes related to metabolic pathways was suppressed in the sucker, while basic nutrient uptake functions might be retained (Supplementary Table S12 and S13). This might be an adaptive and survival strategy of sandalwood, which reduced the production of unwanted secondary metabolites in the sucker, thus optimizing resource utilization for better uptake of nutrients from the host. Therefore, this particular *Copia* composition might be related to the parasitism of sandalwood.

In addition, a few researchers have proposed the parasitism might lead to an increase in genome size [17, 38], due to the parasitized plant was liberated from its limitations of the growth rate of the root meristematic tissue, or the resources obtained

from the host. However, this was subsequently refuted in studies of the genus *Cuscuta*, which nonetheless found a correlation between genome size changes and centromere form while no association was found between parasitic forms and genome size. For instance, species with monocentric chromosomes exhibited a 102-fold variation in genome size and a higher basic chromosome number, whereas species with holocentric chromosomes had modest genome sizes [17]. In Sandalwood, we also discovered that its parasitism did not lead to genome expansion. As such, the genome size of the non-parasitized *M. oleifera* was 1.5 Gb, whereas the genome size of hemiparasitic species *T. chinensis* was 521.90 Mb, and the genome size of hemiparasitic species *S. album* was 218.90 Mb. However, we found that the *Copia* was positively correlated with the genome size. We discovered that chromosomes with longer centromeres had more *Copia* and centromere-specific *Copia*. This suggested that the *Copia* contents promoted the length expansion of both the centromeres and the chromosomes.

In conclusion, we constructed the first T2T genome of sandalwood by combining HiFi, Hi-C, and ultra-long ONT data. We resolved its sequence composition and function of telomeres and centromeres, and provided new insights into genome evolution in parasitic plants.

# Methods

## Plant materials and genome sequencing

Genomic DNA was extracted from leaves collected at the Experimental Station of the Research Institute of Tropical Forestry, Chinese Academy of Forestry, Guangzhou,

China. Then, the extracted DNA was assessed for concertation and quality using NanoDrop 2000 and used to construct linked read libraries using a PacBio SMRTbell library from a SMRTbell Prep Kit 3.0 (PN: 102-182-700), following the manufacturer's protocols and then sequenced on the PacBio Revio platform for generating HiFi reads. For ONT ultra-long sequencing, the standard library was prepared using the SQK-LSK109 kit, following the standard protocol. The purified library was sequenced using a PromethION sequencer (Oxford Nanopore Technologies, Oxford, UK). For RNA-seq, phenol/chloroform was used to isolated RNA from root, sucker, stem, and leaf samples (three bioreplicates) and checked for purity and integrity before constructin. The libraries for all four tissues were prepared using mRNA-seq preparation kits, and sequenced using PE150 mode on MGISEQ-2000 platform.

**Genome assembly and quality evaluation**

The de novo assembly were performed using Hifiasm (v0.19.6-r595) [39]. For Hi-C sequence data [26], we used Juicer (v1.6) [40] and bowtie2 (v2.3.2) [41] to filter out low-quality and unvalidated paired-end reads and construct interaction matrices to obtain chromosome-scale genomes. Redundant contigs were removed by Purge_Haplotigs (v1.1.2) [42]. Then, the draft genome was subjected to a final round of gap filling using ONT ultra-long reads corrected by NextDenovo (v2.17-r941) [43] with LR_Gapcloser (v1.9.4) [44] and Minimap2 (v2.24-r1122) [45] to obtain a T2T genome.

The completeness of the assembled Sal_t2t genome sequences was analyzed

using BUSCO (v5.3.2) [46] with the embryophyta_odb10 databases (issued 2020-08-05, including 1614 proteins). To measure genome coverage based on read mapping rates, NGS short reads [26], HiFi reads and RNA-seq reads were mapped against the assembled genome sequences by BWA-MEM (v0.7.9a, https://github.com/lh3/bwa), minimap2 and HISAT2 (v2.2.1) [47]. The distribution of GC content was used to detect sample contamination.

**Genome annotation**

To identify repeat sequences Sal_t2t and other three Santalales species, several programs in EDTA (v1.9.4) [48], including LTR_FINDER, LTRharvest, LTR_retriever, Generic Repeat Finder, HelitronScanner, TIR-Learner, RepeatMasker, and RepeatModeler, as well as a series of integration scripts, were used to annotate and identify LTR, LINE, SINE, Helitron, MITE, and other retrotransposon and transposon sequences. In addition, we utilized Tandem Repeat Finder (TRF v4.09, http://tandem.bu.edu/trf/trf.html) [49] to independently predict tandem repeats in the genome.

To annotate the gene structure, we used GETA pipline (v2.5.1, https://github.com/chenlianfu/geta) with three methods: homology, de novo, and transcript-based annotation. The published protein information of *Vitis vinifera*, *Arabidopsis thaliana*, *M. oleifera*, V1 and V2 genomes were used as homology references.

Finally, we used CMSScan (v1.1.4, https://github.com/ajinabraham/CMSScan) to mine ncRNA information with the Rfam nonredundant database, which is based on

the homology annotation of ncRNAs, including tRNA, rRNA, miRNA, and snRNA.

**Genome comparison**

V1 assembled genome, and *M. oleifera* genome were downloaded from the CNCB (https://www.cncb.ac.cn/) under accession number PRJCA009490 and PRJNA472200. V2 assembled genome and annotation were downloaded from the Figshare database (10.6084/m9.figshare.23694729.v1). *T. chinensis* genome was downlowd from NCBI under PRJNA855314.

The Synteny and Rearrangement Identifier (SyRI v1.5.4) [50] was utilized for detecting collinearity, SVs, and PAVs among three versions of sandalwood genomes. Then, ONT ultra-long reads and HiFi reads were used to validate the accuracy of the assembly of Sal_t2t with reads mapping.

**Organelle genome assembly**

GSAT (v1.11, https://github.com/hwc2021/GSAT) [51] was used to assemble mitochondria genome with 4 Gb of Illumina reads and all HiFi reads. SPAdes (v3.15.5) [52] was used to assemble chloroplast genome with 5 Gb of Illumina reads. The complete CP and MT assemblies were visualized with Bandage (v0.9.0) [53] to remove contigs with abnormal coverage and simplify genome with the organelle genomes downloaded from NCBI (NC_081498.1 and NC_048953.1) used as reference.

**Organelle gene transfer**

Based on the assembly and annotation files of the *S. album* nuclear genome, Blastn (v0.8.1) software was used to identify transfer events from organelle to nuclear

genomes with default parameters. We filtered the transfer fragments less than 30bp in length and the identify score less than 80%. We extracted the 500bp,1000bp and 2000bp upstream and downstream of the transferred fragments for TE statistics combined with EDTA annotation results. Visualization of these results was implemented with ggplot2 (https://github.com/tidyverse/ggplot2) package in R.

**Identification of telomeres and centromeres**

Referring to the research methods used in the grape (PN40024) genome [9], we used TIDK (v.0.2.0, https://github.com/tolkit/telomeric-identifier), TRF for the identification of centromeres and telomeres, and combined with the result from quarTeT (v1.1.4) [54], EDTA pipeline and srf (https://github.com/lh3/srf) [55] as a complement. The telomere repeat units were explored by TIDK with options "tidk explore -f genome.fa -minimum 5 -maximum 12 -o tidk_explore -t 2 -log -dir telomere_find -extension TSV". Then the whole genome was searched using the following parameters "tidk search -f genome.fa -s AAACCCT -o tidk_search -dir telomere_find".

For centromere annotation, we used the candidate regions identified by quarTeT and the blank regions in Hi-C contact matrices referring to the method in faba genome [56], and extracted all TE sequences presented in the candidate centromeric regions and calculated the length and count of these TEs. We selected the top 10 TEs and found three *Copia* repeats (TE_00001095, TE_00001228, TE_00001258) and one unknown LTR repeat (TE_00000831) were mainly enriched in candidate regions. TRF was used to scan tandem repeats ranging from 30 to 500 base pairs in the

genome with the parameters "2 7 7 80 10 50 500 -f -d -m", and then, we merged the results of annotation by using trf2gff in TRF. We visualized the top 30 repetitive sequences in terms of total count and length with ggplot2 package in R but could not find the enrichment of tandem repeats in most centromeres. Then we used srf to identify HORs and find only one HOR located in all chromosomes. Combined with TRF results, this HOR were composed of two 20 bp tandem repeat sequences and considered to be the characteristic of centromeric satellites. To complete the data statistics and visualization, we used information from TRF, SRF and EDTA results extracted by the AWK command in the Linux system and analyzed the results in IGV (v.2.12.3). Fitting and visualization were implemented with ggplot2 package in R. We considered the intersection region of TRs (from srf) and TEs (from EDTA pipeline and TIDK) as the centromere region.

The candidate centromeres in *M. oleifera*, *T. chinensis* and V2 were identified with the same method as mentioned above.

**Evolution analysis of CRs in Santalales**

We used BEDTools (v2.30.0) [57] to extracted the CRs and aligned using MAFFT (v7.480) [58] Then we used BMGE (v1.12) [59] to remove ambiguously aligned regions with options "-g 0.85 -h 1 -b 1 -w 1". We constructed the ML tree by using FastTree (v2.1.10) [60] with GTR model. Finally, the tree was adjusted, customized and displayed by iTOL (version, https://itol.embl.de/itol.cgi) [61].

## Data Availability

All sequencing data including Hifi, ONT, and RNA-seq have been submitted to the CNCB (https://www.cncb.ac.cn/) under accession number PRJCA026960. The nuclear genome assembly and annotations, chloroplast genome, and mitochondrial genome have been deposited to figshare (10.6084/m9.figshare.26019481).

## Authors' contributions

Xuezhu Liao, Zhiqiang Wu and Zhou Hong provided the ideas and frame of this paper. Dan Peng performed the bioinformatic analysis and drafted the manuscript with Xuezhu Liao. Dan Peng and Xuezhu Liao revised the manuscript with the help of Zhiqiang Wu and Zhou Hong. Shenglong Kan and Zhou Hong prepared the sequencing samples. All authors read and approved the final manuscript.

## Funding

This work was funded by the Guangdong Pearl River Talent Program (grants 2021QN02N792) and the Chinese Academy of Agricultural Sciences Elite Youth Program (110243160001007).

## Competing Interests

The authors declare that they have no competing interests.

**Table 1.** Comparision of three sandalwood assemblies.

| | Sal_t2t | V1 (Hong) | V2 (Zhang) |
|---|---|---|---|
| Total length (Mb) | 218.90 | 229.60 | 207.45 |
| Gaps | 0 | 23 | 108 |
| Contig N50 (Mb) | 18.40 | 12.75 | 5.56 |
| BUSCO - genome | C:98.3%[S:96.4%, D:1.9%] | C:98.2%[S:96.3%, D:1.9%] | C:98.0%[S:95.4%, D:2.6%] |
| RNA reads mapping rate - Leaf | 96.12% | 96.05% | 92.54% |
| NGS reads mapping rate | 99.65% | 98.81% | 96.93% |
| HiFi reads mapping rate | 99.97% | 99.98% | 99.48% |
| TE content | 25.34% | 28.93% | 22.05% |
| TE content - Copia | 6.63% | 6.10% | 5.73% |
| Gene counts | 24,171 | 21,673 | 23,282 |
| BUSCO - gene set | C:96.0%[S:93.6%, D:2.4%] | C:93.5%[S:91.3%, D:2.2%] | C:89.6%[S:86.9%, D:2.7%] |
| rRNAs | 3,910 | 7,720 | 610 |
| tRNAs | 567 | 614 | 506 |
| Transfer count - mitochondria | 2,416 | 2,400 | 2,246 |
| Transfer count - chloroplast | 2,620 | 2,515 | 2,299 |

## Figures

**Figure 1: Assembly and annotation of Sal_t2t.** (A) Heatmap of genomic interactions of Sal_t2t genome. (B) Characterization of Sal_t2t genome. The density of genes, TEs and GC content were calculated per 200 Kb. (C) Translocations in Chr4 (Sal_t2t) vs Chr02 (V2). Dots and lines represented chromosomes alignments between Sal_t2t, V1 (red) and V2 (blue). (D) Translocations in Chr10 (Sal_t2t) vs Chr01 (V2). The other contents were the same as panel a. (E) Alignments between Chr3 (Sal_t2t), Chr04 (V1) and Chr03 (V2) from 5Mb to 20 Mb. Deep red thick lines represented the centromere region. (F) Proportion of TE elements in three sandalwood assemblies. (G) BUSCO assessment of genomes and gene sets in three sandalwood assemblies.

**Figure 2: Organelle gene transfer.** (A) Counts of NUPTs and NUMTs in three sandalwood assemblies. (B) Length of NUPTs and NUMTs in three sandalwood assemblies. (C - F) Simplified comparison of the chloroplast genome and three nuclear genome assemblies: (C) Overall deletion of a neighboring chloroplast fragment transferred multiple times; (D) Partial copy number deletion of a chloroplast fragment transferred to a different chromosome; (E) Partial copy number deletion of a chloroplast fragment transferred to the same chromosome; (F) Complete deletion of a chloroplast fragment transferred to a chromosome. (G) Counts of intact TEs in flanking regions of transfer fragments. (H) Counts of DNA and Unknown type of TEs in flanking regions of transfer fragments.

**Figure 3: Characteristics and distribution of repeats in centromeres.** (A) Heatmap

of genomic interactions of each chromosome in Sal_t2t genome. (B) Best candidate regions predicted by quarTeT. (C) Distribution of CRs. (D) HORs regions predicted by SRF. (E) Distribution of TRs in HORs. (F) The final centromere regions.

**Figure 4: Comparison of centromeric repeats in Santalales.** (A) Scatter plot of TRs in HORs of four Santalales genomes/assembles. Counts of each TR unit associated with circle sizes. Shadows in the background represented the roughly distributed areas of TRs in each species. (B) Phylogeny of CRs of four Santalales genomes/assembles without branch length. Different colors in the inner ring represented TE sequences in each species. (C) Proportions of TE and TR in centromere regions of four Santalales genomes/assembles.

**Figure 5: Relationship between centromere and chromosome characteristics.** (A) Genome-wide fitting curve of TE coverage (windows: 500 Kb). (B) Counts of each TE types in centromere regions. (C) Point plot and linear correlation analysis. * represented $p < 0.05$, ** represented $p < 0.01$. Chr: chromosome. (D) TE insertion statistics in centromic genes. (E) Comparison of expression among centromere genes and genes in other regions (*wilcox* test, ** represents $p < 0.01$). The circle represented the mean expression and the vertical line represented the standard deviation. (F) Relationship between TE insertion and expression at the genome-wide genes. Genefl, flanking region of genes.

## Supplementary Material

**Supplementary Fig. S1.** Structural variation between three Santalales assemblies.

**Supplementary Fig. S2.** Density distribution of transfer fragment length of three

sandalwood assemblies. Cp: chloroplast genome, Mt: mitochondrial genome.

**Supplementary Fig. S3.** Counts of TE insertions in different flanking region (bp) of transfer fragments.

**Supplementary Fig. S4.** Counts of different types of TE insertions in different flanking regions (bp) of transfer fragments.

**Supplementary Fig. S5.** Distribution of top 20 TRs in each chromosome.

**Supplementary Fig. S6.** Count distribution of different type of TEs in each chromosome.

**Supplementary Fig. S7.** TE insertion statistics in all genes. Genefl, flanking region of genes; CDS: Coding Sequence; Intron: intronic regions.

**Supplementary Fig. S8.** Relationship between TE insertion and expression in root and sucker. Genefl, flanking region of gene; CDS: Coding Sequence; Intron: intronic region.

## Reference


1. Li H, Durbin R. Genome assembly in the telomere-to-telomere era. *Nat Rev Genet* 2024. https://doi.org/10.1038/s41576-024-00718-w.
2. Sohn JI, Nam JW. The present and future of de novo whole-genome assembly. *Brief Bioinform* 2018, 19(1):23-40. https://doi.org/10.1093/bib/bbw096.
3. Kong W, Wang Y, Zhang S, et al. Recent advances in assembly of complex plant genomes. *Genom Proteom Bioinf* 2023, 21(3):427-439. https://doi.org/10.1016/j.gpb.2023.04.004.
4. Chen J, Wang ZJ, Tan KW, et al. A complete telomere-to-telomere assembly of the maize genome. *Nat Methods* 2023, 55(7):1221-1231. https://doi.org/10.1038/s41588-023-01419-6.
5. Wang B, Yang X, Jia Y, et al. High-quality *Arabidopsis thaliana* genome assembly with Nanopore and HiFi long reads. *Genom Proteom Bioinf* 2022, 20(1):4-13. https://doi.org/10.1016/j.gpb.2021.08.003.
6. Shang L, He W, Wang T, et al. A complete assembly of the rice Nipponbare reference genome. *Mol Plant* 2023, 16(8):1232-1236. https://doi.org/10.1016/j.molp.2023.08.003.
7. Han X, Zhang YL, Zhang Q, et al. Two haplotype-resolved, gap-free genome assemblies for

*Actinidia latifolia* and *Actinidia chinensis* shed light on the regulatory mechanisms of vitamin C and sucrose metabolism in kiwifruit. *Mol Plant* 2023, 16(2):452-470. https://doi.org/10.1016/j.molp.2022.12.022.

8. Deng Y, Liu SC, Zhang YL, et al. A telomere-to-telomere gap-free reference genome of watermelon and its mutation library provide important resources for gene discovery and breeding. *Mol Plant* 2022, 15(8):1268-1284. https://doi.org/10.1016/j.molp.2022.06.010.

9. Shi XY, Cao S, Wang X, et al. The complete reference genome for grapevine (*Vitis vinifera* L.) genetics and breeding. *Hortic Res* 2023, 10(05):uhad061. https://doi.org/10.1093/hr/uhad061.

10. Lan L, Leng L, Liu W, et al. The haplotype-resolved telomere-to-telomere carnation (*Dianthus caryophyllus*) genome reveals the correlation between genome architecture and gene expression. *Hortic Res* 2024, 11(1):uhad244. https://doi.org/10.1093/hr/uhad244.

11. Bai M, Jiang S, Chu S, et al. The telomere-to-telomere (T2T) genome of *Peucedanum praeruptorum* Dunn provides insights into the genome evolution and coumarin biosynthesis. *GigaScience* 2024, 13:giae025. https://doi.org/10.1093/gigascience/giae025.

12. Zhou J, Liu Y, Guo X, et al. Centromeres: From chromosome biology to biotechnology applications and synthetic genomes in plants. *Plant Biotechnol J* 2022, 20(11):2051-2063. https://doi.org/10.1111/pbi.13875.

13. Oliveira LC, Torres GA. Plant centromeres: genetics, epigenetics and evolution. *Mol Biol Rep* 2018, 45(5):1491-1497. https://doi.org/10.1007/s11033-018-4284-7.

14. Liu Y, Yi C, Fan C, et al. Pan-centromere reveals widespread centromere repositioning of soybean genomes. *Proc Natl Acad Sci U S A* 2023, 120(42):e2310177120. https://doi.org/10.1073/pnas.2310177120.

15. Naish M, Henderson IR. The structure, function, and evolution of plant centromeres. *Genome Res* 2024, 34(2):161-178. https://doi.org/10.1101/gr.278409.123.

16. Neumann P, Navratilova A, Koblizkova A, et al. Plant centromeric retrotransposons: a structural and cytogenetic perspective. *Mob DNA* 2011, 2(1):4. https://doi.org/10.1186/1759-8753-2-4.

17. Neumann P, Oliveira L, Cizkova J, et al. Impact of parasitic lifestyle and different types of centromere organization on chromosome and genome evolution in the plant genus *Cuscuta*. *New Phytol* 2021, 229(4):2365-2377. https://doi.org/10.1111/nph.17003.

18. Sanchez-Puerta MV, Ceriotti LF, Gatica-Soria LM, et al. Invited Review Beyond parasitic convergence: unravelling the evolution of the organellar genomes in holoparasites. *Ann Bot* 2023, 132(5):909-928. https://doi.org/10.1093/aob/mcad108.

19. Harbaugh DT, Baldwin BG. Phylogeny and biogeography of the sandalwoods (*Santalum*, Santalaceae): repeated dispersals throughout the Pacific. *Am J Bot* 2007, 94 6:1028-1040. https://doi.org/10.3732/ajb.94.6.1028

20. Scartezzini P, Speroni E. Review on some plants of Indian traditional medicine with antioxidant activity. *Journal of ethnopharmacology* 2000, 71(1-2):23-43. https://doi.org/10.1016/s0378-8741(00)00213-0.

21. Kumar ANA, Joshi G, Ram HYM. Sandalwood: history, uses, present status and the future. *Curr Sci* 2012, 103(12):1408-1416.

22. Yang TQ, Zhang RA, Tian XL, et al. The chromosome-level genome assembly and genes involved in biosynthesis of nervonic acid of *Malania oleifera*. *Sci Data* 2023, 10(1):298. https://doi.org/10.1038/s41597-023-02218-8.

23. Chen X, Fang D, Xu Y, et al. *Balanophora* genomes display massively convergent evolution with other extreme holoparasites and provide novel insights into parasite–host interactions. *Nat Plants* 2023, 9(10):1627-1642. https://doi.org/10.1038/s41477-023-01517-7.

24. Fu JN, Wan LY, Song LS, et al. Chromosome-level genome assembly of the hemiparasitic *Taxillus chinensis* (DC.) Danser. *Genome Biol Evol* 2022, 14(5):evac060. https://doi.org/10.1093/gbe/evac060.

25. Lyko P, Wicke S. Genomic reconfiguration in parasitic plants involves considerable gene losses alongside global genome size inflation and gene births. *Plant Physiol* 2021, 186(3):1412-1423. https://doi.org/10.1093/plphys/kiab192.

26. Hong Z, Peng D, Tembrock LR, et al. Chromosome-level genome assemblies from two sandalwood species provide insights into the evolution of the Santalales. *Commun Biol* 2023, 6(1):587. https://doi.org/10.1038/s42003-023-04980-2.

27. Zhang XH, Li MZ, Bian Z, et al. Improved chromosome-level genome assembly of Indian sandalwood (*Santalum album*). *Sci Data* 2023, 10(1):921. https://doi.org/10.1038/s41597-023-02849-x.

28. Wang H, Liao X, Tembrock LR, et al. Evaluation of intracellular gene transfers from plastome to nuclear genome across progressively improved assemblies for *Arabidopsis thaliana* and *Oryza sativa*. *Genes-Basel* 2022, 13(9):1620. https://doi.org/10.3390/genes13091620.

29. Chen C, Wu S, Sun Y, et al. Three near-complete genome assemblies reveal substantial centromere dynamics from diploid to tetraploid in *Brachypodium* genus. *Genome Biol* 2024, 25(1):63. https://doi.org/10.1186/s13059-024-03206-w.

30. Naish M, Alonge M, Wlodzimierz P, et al. The genetic and epigenetic landscape of the *Arabidopsis* centromeres. *Science* 2021, 374(6569):eabi7489. https://doi.org/10.1126/science.abi7489.

31. Qing L. Research progress on structure and evolution of plant centromeres. *J Trop and Subtrop Bot* 2015, 23(5):576-586. https://doi.org/10.11926/j.issn.1005-3395.2015.05.013.

32. Wang G, Zhang X, Jin W. An overview of plant centromeres. *J Genet Genomics* 2009, 36(9):529-537. https://doi.org/10.1016/S1673-8527(08)60144-7.

33. Shi X, Cao S, Wang X, et al. The complete reference genome for grapevine (Vitis vinifera L.) genetics and breeding. *Hortic Res* 2023, 10(5):uhad061. https://doi.org/10.1093/hr/uhad061.

34. Chang X, He X, Li J, et al. High-quality *Gossypium hirsutum* and *Gossypium barbadense* genome assemblies reveal the landscape and evolution of centromeres. *Plant Commun* 2024, 5(2):100722. https://doi.org/10.1016/j.xplc.2023.100722.

35. Zhang H, Wang K. The research progress on plant functional centromere DNAs. *Curr Biotechnol* 2022, 12(1):1-9.

36. Lv Y, Liu C, Li X, et al. A centromere map based on super pan-genome highlights the structure and function of rice centromeres. *J Integr Plant Biol* 2024, 66(2):196-207. https://doi.org/10.1111/jipb.13607.

37. Guo N, Wang S, Gao L, et al. Genome sequencing sheds light on the contribution of structural variants to *Brassica oleracea* diversification. *BMC Biol* 2021, 19(1):93. https://doi.org/10.1186/s12915-021-01031-2.

38. Plackova K, Bures P, Zedek F. Centromere size scales with genome size across Eukaryotes. *Sci Rep* 2021, 11(1):19811. https://doi.org/10.1038/s41598-021-99386-7.

39. Cheng H, Concepcion GT, Feng X, et al. Haplotype-resolved de novo assembly using phased

assembly graphs with hifiasm. *Nat Methods* 2021, 18(2):170-175. https://doi.org/10.1038/s41592-020-01056-5.

40. Durand NC, Shamim MS, Machol I, et al. Juicer provides a one-click system for analyzing loop-resolution Hi-C experiments. *Cell Syst* 2016, 3(1):95-98. https://doi.org/10.1016/j.cels.2016.07.002.

41. Langmead B, Salzberg SL. Fast gapped-read alignment with Bowtie 2. *Nat Methods* 2012, 9(4):357-359. https://doi.org/10.1038/nmeth.1923.

42. Roach MJ, Schmidt SA, Borneman AR. Purge Haplotigs: allelic contig reassignment for third-gen diploid genome assemblies. *BMC Biol* 2018, 19(1):460. https://doi.org/10.1186/s12859-018-2485-7.

43. Hu J, Wang Z, Sun Z, et al. NextDenovo: an efficient error correction and accurate assembly tool for noisy long reads. *Genome Biol* 2024, 25(1):107. https://doi.org/10.1186/s13059-024-03252-4.

44. Xu G-C, Xu T-J, Zhu R, et al. LR_Gapcloser: a tiling path-based gap closer that uses long reads to complete genome assembly. *GigaScience* 2018, 8(1):giy157. https://doi.org/10.1093/gigascience/giy157.

45. Li H. Minimap2: pairwise alignment for nucleotide sequences. *Bioinformatics* 2018, 34(18):3094-3100. https://doi.org/10.1093/bioinformatics/bty191.

46. Simao FA, Waterhouse RM, Ioannidis P, et al. BUSCO: assessing genome assembly and annotation completeness with single-copy orthologs. *Bioinformatics* 2015, 31(19):3210-3212. https://doi.org/10.1093/bioinformatics/btv351.

47. Kim D, Langmead B, Salzberg SL. HISAT: a fast spliced aligner with low memory requirements. *Nat Methods* 2015, 12(4):357-360. https://doi.org/10.1038/nmeth.3317.

48. Ou S, Su W, Liao Y, et al. Benchmarking transposable element annotation methods for creation of a streamlined, comprehensive pipeline. *Genome Biol* 2019, 20(1):275. https://doi.org/10.1186/s13059-019-1905-y.

49. Benson G. Tandem repeats finder: a program to analyze DNA sequences. *Nucleic Acids Res* 1999, 27(2):573-580. https://doi.org/10.1093/nar/27.2.573.

50. Goel M, Sun H, Jiao WB, et al. SyRI: finding genomic rearrangements and local sequence differences from whole-genome assemblies. *Genome Biol* 2019, 20(1):277. https://doi.org/10.1186/s13059-019-1911-0.

51. He W, Xiang K, Chen C, et al. Master graph: an essential integrated assembly model for the plant mitogenome based on a graph-based framework. *Brief Bioinform* 2023, 24(1):bbac522. https://doi.org/10.1093/bib/bbac522.

52. Prjibelski A, Antipov D, Meleshko D, et al. Using SPAdes de novo assembler. *Curr Protoc Bioinformatics* 2020, 70(1):e102. https://doi.org/10.1002/cpbi.102.

53. Wick RR, Schultz MB, Zobel J, et al. Bandage: interactive visualization of de novo genome assemblies. *Bioinformatics* 2015, 31(20):3350-3352. https://doi.org/10.1093/bioinformatics/btv383.

54. Lin Y, Ye C, Li X, et al. quarTeT: a telomere-to-telomere toolkit for gap-free genome assembly and centromeric repeat identification. *Hortic Res* 2023, 10(8):uhad127. https://doi.org/10.1093/hr/uhad127.

55. Zhang Y, Chu J, Cheng H, et al. De novo reconstruction of satellite repeat units from sequence data. *Genome Res* 2023, 33(11):1994-2001. https://doi.org/10.1101/gr.278005.123.

56. Jayakodi M, Golicz AA, Kreplak J, et al. The giant diploid faba genome unlocks variation in a global protein crop. *Nature* 2023, 615(7953):652-659. https://doi.org/10.1038/s41586-023-05791-5.
57. Quinlan AR, Hall IM. BEDTools: a flexible suite of utilities for comparing genomic features. *Bioinformatics* 2010, 26(6):841-842. https://doi.org/10.1093/bioinformatics/btq033.
58. Nakamura T, Yamada KD, Tomii K, et al. Parallelization of MAFFT for large-scale multiple sequence alignments. *Bioinformatics* 2018, 34(14):2490-2492. https://doi.org/10.1093/bioinformatics/bty121.
59. Criscuolo A, Gribaldo S. BMGE (Block Mapping and Gathering with Entropy): a new software for selection of phylogenetic informative regions from multiple sequence alignments. *BMC Evol Biol* 2010, 10:210. https://doi.org/10.1186/1471-2148-10-210.
60. Price MN, Dehal PS, Arkin AP. FastTree 2--approximately maximum-likelihood trees for large alignments. *PloS one* 2010, 5(3):e9490. https://doi.org/10.1371/journal.pone.0009490.
61. Letunic I, Bork P. Interactive Tree of Life (iTOL) v6: recent updates to the phylogenetic tree display and annotation tool. *Nucleic Acids Res* 2024. https://doi.org/10.1093/nar/gkae268.

Figure 1

Click here to access/download;Figure;Figure 1.pdf

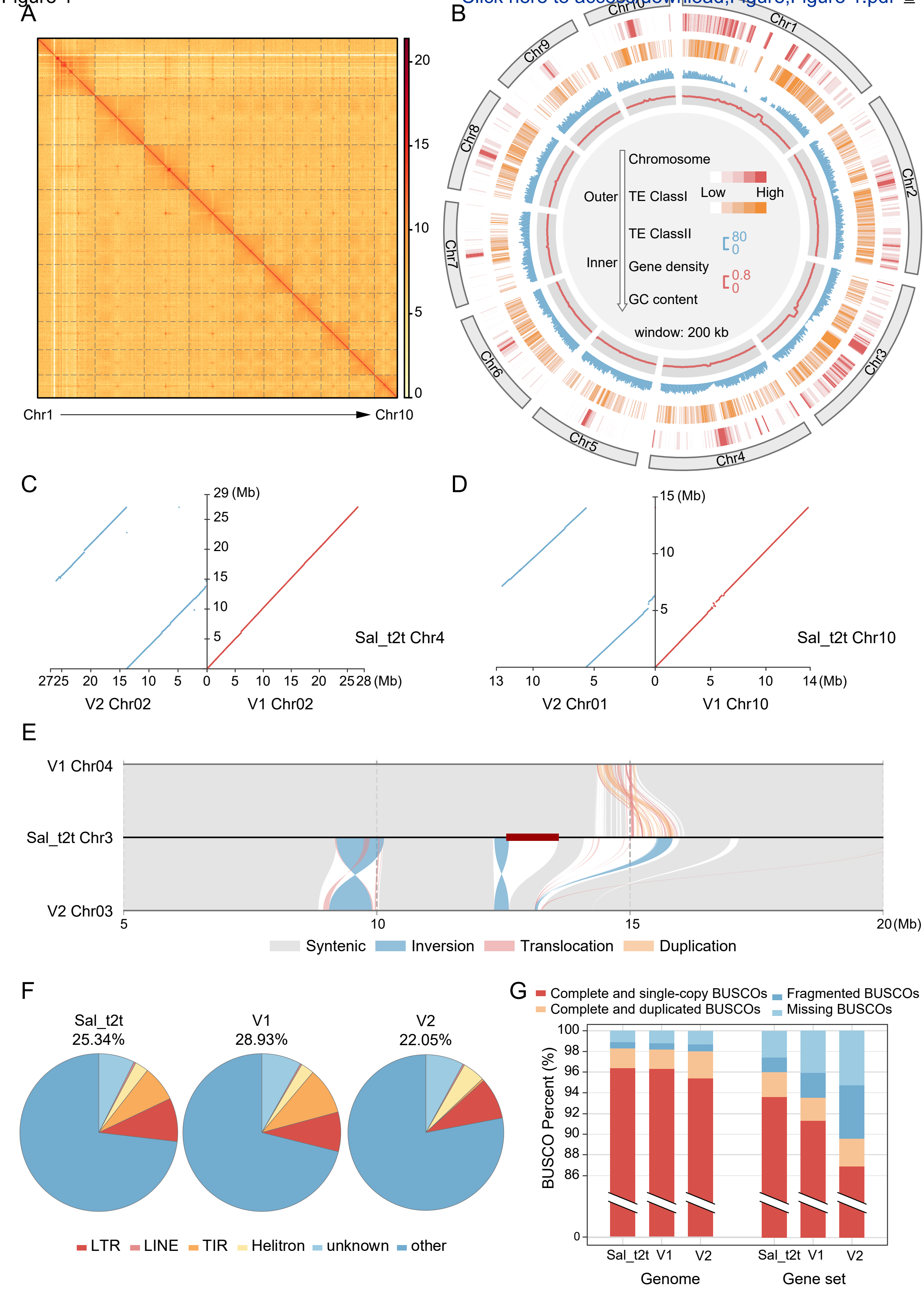

Figure 2

Click here to access/download;Figure;Figure 2.pdf

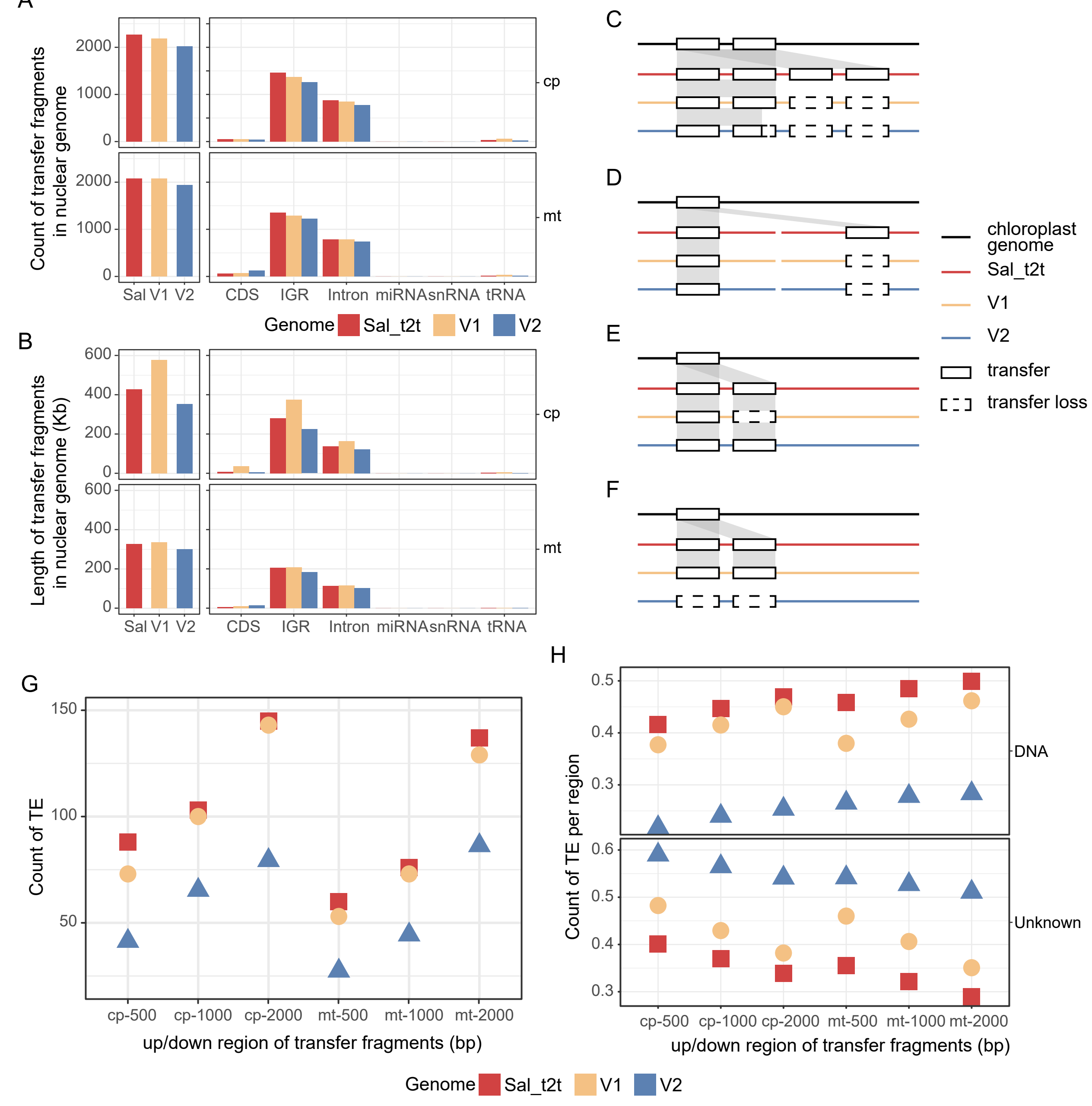

Figure 3

Click here to access/download;Figure;Figure 3.pdf

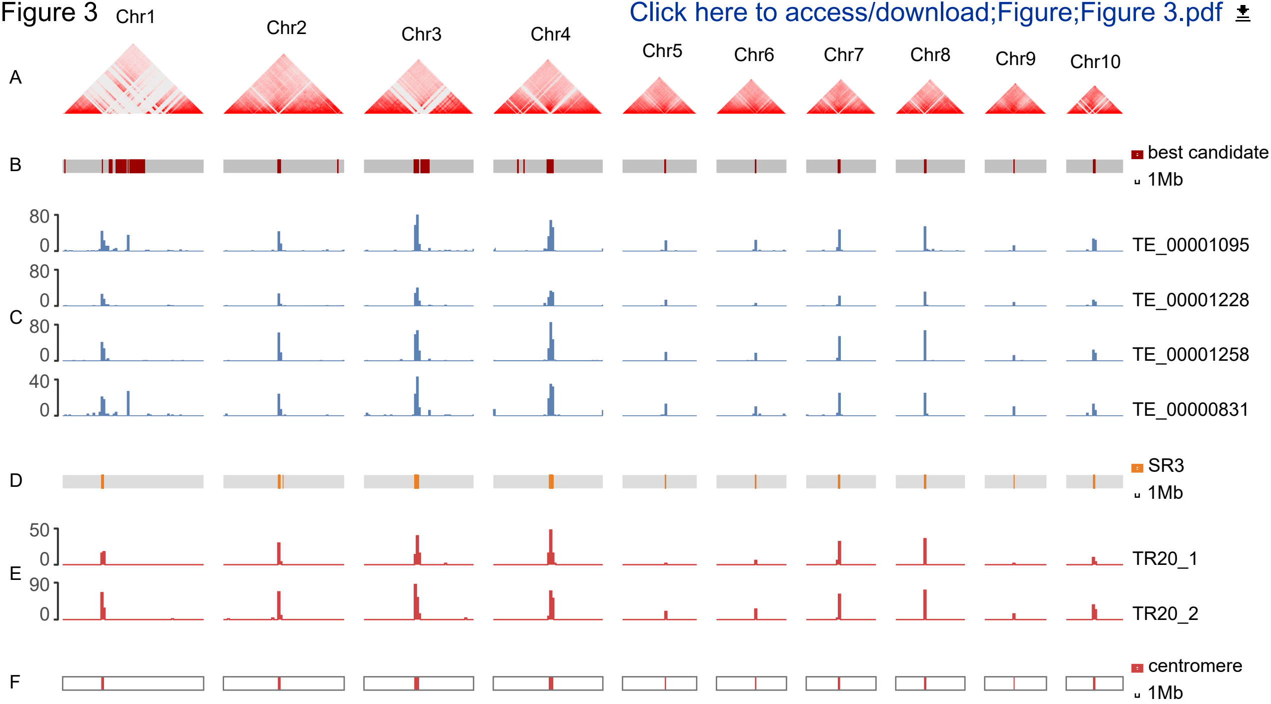

Figure 4

Click here to access/download;Figure;Figure 4.pdf

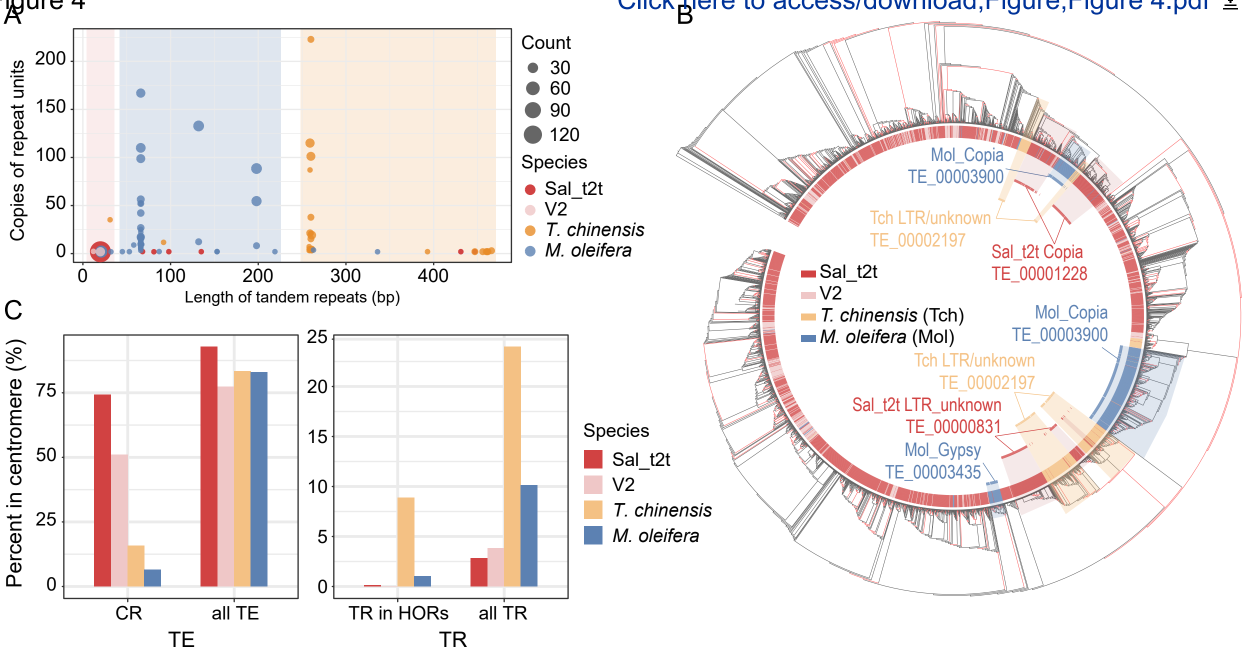

Figure 5

Click here to access/download;Figure;Figure 5.pdf

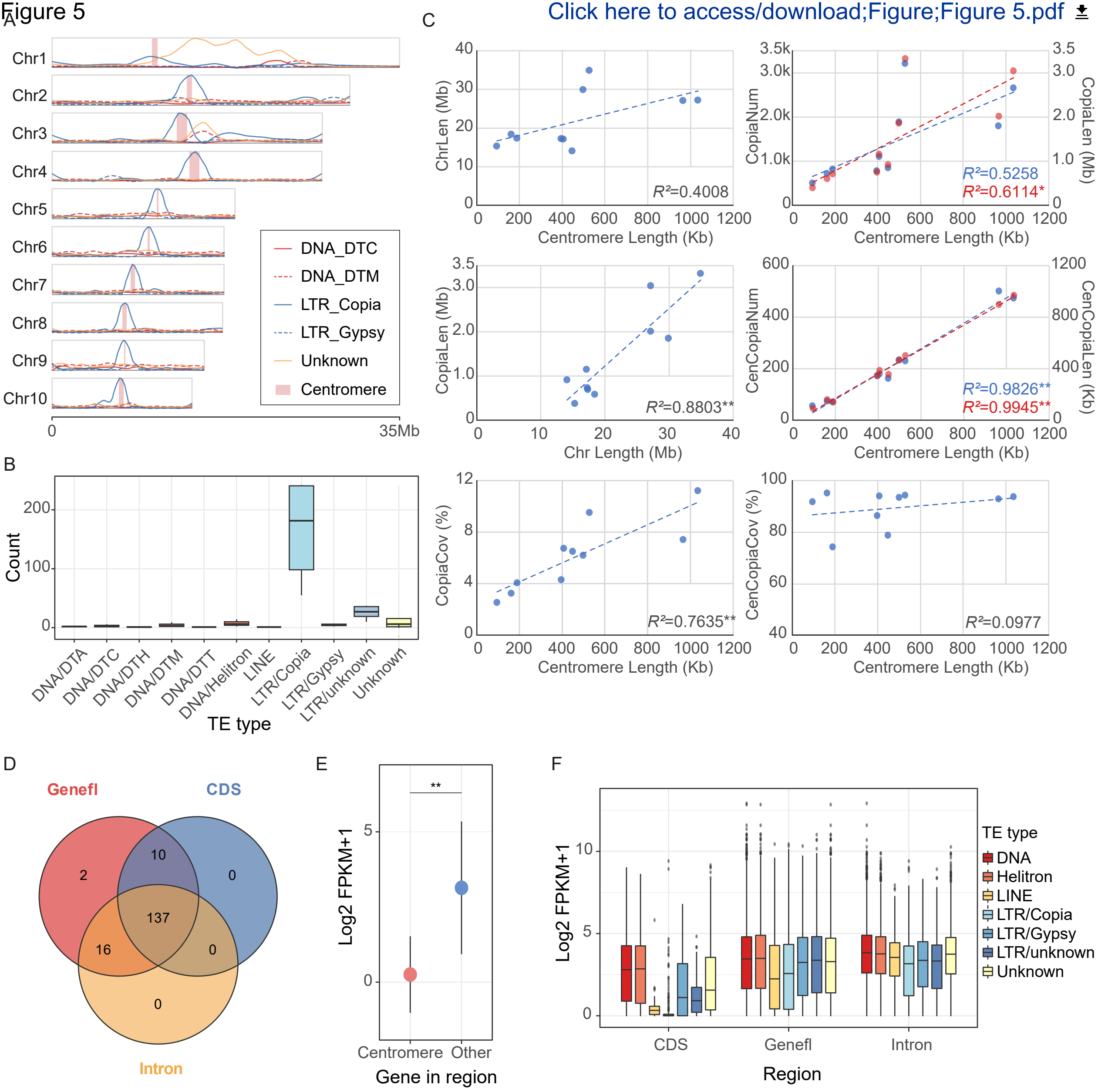

Click here to access/download
**Supplementary Material**
Data Resource.docx

Supplementary Fig. S1

Click here to access/download
**Supplementary Material**
Supplementary Fig. S1.pdf

Supplementary Fig. S2

Click here to access/download
**Supplementary Material**
Supplementary Fig. S2.pdf

Supplementary Fig. S3

Click here to access/download
**Supplementary Material**
Supplementary Fig. S3.pdf

Supplementary Fig. S4

Click here to access/download
**Supplementary Material**
Supplementary Fig. S4.pdf

Supplementary Fig. S5

Click here to access/download
**Supplementary Material**
Supplementary Fig. S5.pdf

Supplementary Fig. S6

Click here to access/download
**Supplementary Material**
Supplementary Fig. S6.pdf

Click here to access/download
**Supplementary Material**
Supplementary Fig. S7.pdf

Supplementary Fig. S8

Click here to access/download
**Supplementary Material**
Supplementary Fig. S8.pdf

Supplementary Table S1-13

Click here to access/download
**Supplementary Material**
Supplementary Table.xlsx

Cover Letter

Click here to access/download;Personal Cover;Cover Letter.docx

Dear *GigaScience* Editorial Board,

Please find enclosed our manuscript entitled, "**The telomere-to-telomere (T2T) genome provides insights into the evolution of specialized centromere sequences in sandalwood**". We assembled the first telomere-to-telomere (T2T) genome of *Santalum album*, a economically hemiparasitic plant in Santalales, and explored its unique centromere composition, evolution, and the improvement in the accuracy of cyto-nuclear transfer identification by the T2T genome assembly. We thought this submission to a special issue of the T2T series would be of interest to the broad readership of the **GigaScience**.

The novelty of our study was summarized in the following aspects:

1. **Our study provided the first T2T genome of a hemiparasitic plant in the Santalales.** This T2T genome had higher assembly quality, no gaps, and significantly improved annotation. Thus, our study provided a high-quality data source for future molecular, genetic, and functional studies of holoparasitic plants.
2. **Unique composition of centromeric sequences.** We identified the sandalwood centromeres were primarily composed of *Copia* transposons and few tandem repeats, differing from those in most published species, which were enriched in tandem repeats and *Gypsy* with only a few *Copia*. We found that the centromeric sequences of sandalwood were unique and had already diverged from other species within Santalales.
3. **T2T assembly is crucial for the study of cyto-nuclear transfers of organellar genes.** The T2T genome allowed for more accurate and effective identification of NUMTs, NUPTs and subsequent research than the previous assemblies. This indicated that previous genomes might have imperfections, and T2T-level assembly could enhance the accuracy of cyto-nuclear transfer studies.

We had read the "Editorial Policies & Reporting Standards" and fulfill the requirements in a sequential manner. We declared no competing interests and confirmed

that all authors have approved submission of this manuscript, and that the manuscript was not currently submitted elsewhere. We looked forward to your response and were ready to provide any further information required.

Suggested peer reviewers:

Prof. Jiayu Xue,

E-mail: xuejy@njau.edu.cn.

Prof. Jing Wang,

Lab homepage: https://jingwanglab.org/, E-mail: wangjing2019@scu.edu.cn

Prof. Tao Ma,

E-mail: matao.yz@gmail.com

Prof. Gong Lei

E-mail: goongl100@nenu.edu.cn

Yours sincerely,

Prof. Zhiqiang Wu, and Dr. Xuezhu Liao

Shenzhen Branch, Guangdong Laboratory of Lingnan Modern Agriculture, Key Laboratory of Synthetic Biology, Ministry of Agriculture and Rural Affairs, Agricultural Genomics Institute at Shenzhen, Chinese Academy of Agricultural Sciences, Shenzhen, 518120, China

E-mail: wuzhiqiang@caas.cn, liaoxuezhu@caas.cn
