# Supplementary material for: The telomere-to-telomere (T2T) genome provides insights into the evolution of specialized centromere sequences in sandalwood
Source: Gigascience. 2024 Dec 11;13:giae096. doi: 10.1093/gigascience/giae096 (PMC11633456; doi:10.1093/gigascience/giae096)
Supplement: giae096_GIGA-D-24-00225_Revision_1 [file giae096_giga-d-24-00225_revision_1.pdf]

GigaScience

The telomere-to-telomere (T2T) genome provides insights into the evolution of specialized centromere sequences in sandalwood

--Manuscript Draft--

**Manuscript Number:**	GIGA-D-24-00225R1
**Full Title:**	The telomere-to-telomere (T2T) genome provides insights into the evolution of specialized centromere sequences in sandalwood
**Article Type:**	Research
**Funding Information:**	Guangdong Pearl River Talent Program (2021QN02N792) — Dr Zhiqiang Wu<br>Chinese Academy of Agricultural Sciences Elite Youth Program (110243160001007) — Dr Zhiqiang Wu
**Abstract:**	Background<br>Sandalwood, a prized hemiparasitic plant, is highly sought in the commercial market because of its aromatic core materia. The structure and stability of the genome are instrumental in the rapid adaptation of parasitic plants to their surroundings. However, there is a conspicuous lack of research on the genomic-level adaptive evolution of sandalwood.<br>Results<br>In this study, we assembled a gap-free telomere-to-telomere (T2T) reference genome for Santalum album using PacBio HiFi, Hi-C, and ultra-long ONT data. The T2T reference genome (Sal_t2t) encompassed annotations of 24,171 genes and 25.34% repetitive sequences, in addition to all 10 centromeres and 20 telomeres across the 10 chromosomes. The results revealed that the three distinct parasitic species of Santalales had diverse centromeric compositions. The Copia-type LTR transposon emerged as the most significant in the S. album genome, constituting the primary sequence of the centromere and influencing gene expression. Third, in sandalwood, the presence of Copia affected the size of the centromeres, and consequently, the genome size. Identification of the sandalwood T2T genome in this study also enabled the identification of more precise organelle transfer fragments.<br>Conclusions<br>Our research provides a sandalwood T2T genome, laying the groundwork for future investigations on the evolution of energy organs in parasitic plants. Moreover, it offers novel insights into the function and evolution of centromeres, as well as the mechanisms of adaptation and parasitism.
**Corresponding Author:**	Zhiqiang Wu<br>Chinese Academy of Agricultural Sciences Agricultural Genomes Institute at Shenzhen<br>Shenzhen, Guangdong CHINA
**Corresponding Author Secondary Information:**	
**Corresponding Author's Institution:**	Chinese Academy of Agricultural Sciences Agricultural Genomes Institute at Shenzhen
**Corresponding Author's Secondary Institution:**	
**First Author:**	Dan Peng
**First Author Secondary Information:**	
**Order of Authors:**	Dan Peng
	Zhou Hong
	Shenglong Kan
	Zhiqiang Wu
	Xuezhu Liao
**Order of Authors Secondary Information:**	

Powered by Editorial Manager® and ProduXion Manager® from Aries Systems Corporation

Response to Reviewers:	Reviewer #1

Reviewer #1

Comment 1: Please present the results and discussion objectively to reduce the occupating coefficient of some words, such as "our" and "we".
Response: Thank you for your suggestion. We have made the corrections in the corresponding sections of the manuscript.

Comment 2: The language should be improved with native English speakers.
Response: Thanks to your advice, we have revised the inaccurate statements and worked with a professional language editing service to improve the manuscript's wording.

Comment 3: There were lots of abbreviation in the manuscript, please provide a section of abbreviation list at the end of manuscript.
Response: We apologize for not clearly labeling the abbreviations and have added an "Abbreviations" section in line 522, page 25.

Comment 4: Line 53,identify the exact representation of "its".
Response: Thank you. The revised wording now reads as (line 57, page 4):
"These areas are often challenging to handle, and complete assemblies may offer a deeper understanding of the structure and function of these unassembled regions."

Comment 5: Line 138, provide the details of paraments for LAI calculation in EDTA, which can be found in the process documents. This result is quite different with the results from LTR_retriever with the recommendation paraments from https://github.com/oushujun/LTR_retriever.
Response: We apologize for the confusion caused by the lack of detail in the methods section. We used the default parameters of LTR_retriever to generate the "*.LAI" file, based on intermediate results from the EDTA pipeline. The command used was: LTR_retriever -genome sal.fa.mod -inharvest sal.fa.mod.EDTA.raw/LTR/sal.fa.mod.rawLTR.scn -threads 24. We have revised the relevant section of the Materials and Methods section, now reading as follows (line 403, page 20):
"The completeness of the assembled Sal_t2t genome sequences was analyzed using BUSCO (v5.3.2) (RRID:SCR_015008) [46] with the embryophyta_odb10 databases (issued 2020-08-05, including 1614 proteins), and the LAI statistic in LTR_retriever (v2.9.0) (RRID:SCR_017623) [47] with default parameters based on intermediate results from the EDTA pipeline (v1.9.4) (RRID:SCR_022063) [48]."

Comment 6: Line 152-153, the Busco of genome with the parament of "-m geno" should be provided, which was higher than other two assembly in Table 1.
Response: Thank you for pointing this out. We have updated the relevant section to reflect the BUSCO score using the "-m geno" parameter, which is indeed higher than the other two assemblies in Table 1. The revised text can be found in the corresponding section (line 152, page 8):
"Moreover, the completeness of the annotations was assessed using BUSCO, revealing that approximately 98.3% and 96.0% of the core genes in the assembly and gene set were complete, which was much higher than those of the V1 (98.2% and 93.5 %) and V2 (98.0% and 89.6 %) versions (Fig. 1G and Table 1)."

Comment 7: Line 219, the region of centromeric sequences contained high density of TE and some mis-assembly might be obtained in these regions, especially for the previous assembly without T2T technology. So, which methods were adopted in centromeric region identification to reduce the mis-assembly in M. oleifera and T. chinensis.
Response: Thank you for raising this point. To ensure reliable identification of centromeric regions in M. oleifera and T. chinensis, we used three stringent criteria: 1) inclusion of TEs enriched in certain regions while excluding scattered TEs, following the approach for CRs identification in the T2T genome; 2) identification of high-order repeat (HORs), which are key indicators of centromere presence; 3) validation through overlap between HORs and LTR-enriched regions. These regions containing both HORs (TR-enriched) and LTR-enriched were further considered as candidate centromeric regions for M. oleifera and T. chinensis. We focused on unit sequence analysis for centromeric CRs and HORs to minimize assembly errors and believe this multimetric approach provides robust results despite non-T2T assemblies.

Powered by Editorial Manager® and ProduXion Manager® from Aries Systems Corporation

Comment 8: The gypsy content should be added in Table 1.
Response: Thank you for the suggestion. We have updated Table 1 to include the Gypsy contents, as requested (line 547, page 27).

Comment 9: It will be interesting by enrich the increased or disappeared genes in this T2T assembly by comparing with other two assembly.
Response: Thank you for your suggestion. We conducted a comparative analysis of gene annotations between V1 and Sal_t2t assemblies, as V2 was excluded due to poor annotation quality. The Sal_t2t assembly revealed 164 new genes, primarily enriched in metabolic and biosynthetic processes. Of these, 29 genes were located in previously unfilled regions of V1, suggesting possible duplication. Duplication was confirmed for 152 genes, with 56 showing expression, suggesting the assembly captured biologically relevant gene duplicates. The detailed analysis is added to the "Identification of new genes" section (line 160, page 9).
"Identification of new genes
To test whether different assembly methods selectively enrich certain types of genes, we analysed the differences between the annotation results of the two versions, Sal_t2t (Sal_ano) and V1 (V1_ano). Using Liftoff software with Sal_ano and V1_ano as references, 21,583 and 24,007 genes were annotated to the genomes of V1 and Sal_t2t, respectively. Sal_ano contained 1,282 previously unannotated genes, while V1 contained 2,583 genes, which may be due to differences in annotation software and parameters. In addition, the de novo Sal_t2t genome assembled 164 genes, whereas V1 had 92 genes, which were identified as new genes based on Liftoff software (Supplementary Table S5).
In addition, the 164 new genes identified in Sal_t2t were mainly enriched in the GO terms oxidoreductase activity, biosynthetic processes, and metabolic processes (Supplementary Fig. S2). Whereas 29 of these 164 new genes were located in regions located in the filled gaps of V1, and 28 of these new genes were located in the filled regions enriched with rRNAs and TRs in Sal_t2t. These genes showed high similarity and were supported by ultra-long ONT reads, suggesting that they may be duplicated. Classification of all new genes by DupGen_finder software revealed 152 (92.68%) new genes were categorized (dispersed/proximal/tandem/transposed/wgd: 26/60/36/12/18), and the proportion of duplicated genes in the genome-wide background was 70.84%. Moreover, 56 duplicate genes (dispersed/proximal/tandem/transposed/wgd: 4/18/18/5/11) were expressed (Supplementary Table S6)."

Comment 10: In Figure 1A, some blank was exhibited in HiC cluster, why?
Response: Thank you for your question. Similar to what is shown in Figure 3A, these blank regions often correspond to complex areas with a high density of TEs, TRs, or regions enriched with tandem repeats of rRNAs (Supplementary Fig. S11). When mapped to such highly repetitive regions, Hi-C reads with multiple comparisons would be filtered, leading to missing signals in the Hi-C heatmap.

Comment 11: In Figure 1B, why the density of TE and genes were not complementary? Total TE density was suggested to present after Chromosome in Figure 1B.
Response: As shown in Supplementary Fig. S11 above, the second row represents the TE distribution (excluding Chr1), which is complementary to the distribution of genes. Furthermore, we have placed the total TE density after the chromosomes in Figure 1B, as suggested.

Reviewer #2
Comment 1: This study entitled 'The telomere-to-telomere (T2T) genome provides insights into the evolution of specialized centromere sequences in sandalwood' has provided a T2T genome of sandalwood. This genome assembly is pretty well, and it contains all 20 telomeres and 10 centromeres. I think this data needs to be shown in the abstract.
Response: Thank you for your comments. The abstract already includes the number of annotated telomeres and centromeres (lines 33, page 2). For improved clarity, we have replaced the descriptive text with Arabic numerals.

Comment 2: On the other hand, this paper introduced a T2T genome and focused on the repeat sequences and their impacts on genome and expression. But for this sandalwood genome, I still did not find any analysis for the biology points for this

Powered by Editorial Manager® and ProduXion Manager® from Aries Systems Corporation

species. Why the author sequenced this species? Only want to show a T2T genome?

Response: Thank you for your valuable feedback. We understand the importance of addressing the biological significance of the sandalwood genome beyond just showcasing the T2T assembly. Our study goes beyond presenting the T2T sandalwood genome assembly; it aims to investigate the relationships between genomic features and the parasitic forms of the plant. The possibility that parasitic plant centromere length may lead to an increase in genome size has been explored in published articles, and was refuted by recent study confirming that there is no correlation between the form of parasitism and genome size [1, 2]. Building on these findings, our study focused on the centromere sequence composition and its potential relationship with parasitic forms and genome size. In the course of assembling the T2T sandalwood genome, we identified species-specific centromere sequences and explored their evolutionary dynamics in comparison to different parasitic forms. Our results demonstrate that the centromere compositions vary among the three parasitic species studied. Specifically, in sandalwood, the Copia-type LTR transposon predominates in the centromere, impacting gene expression and correlating positively with both centromere size and overall genome size (line 285, page 14).

Moreover, our study aligns with the goals of the T2T special issue in GigaScience, which is why we chose to submit our findings there. We hope this explanation underscores the biological insights our research contributes and addresses the concerns regarding the study's scope.

Reference：

1.Neumann P, Oliveira L, Cizkova J, et al. Impact of parasitic lifestyle and different types of centromere organization on chromosome and genome evolution in the plant genus Cuscuta. New Phytol 2021, 229(4):2365-2377. https://doi.org/10.1111/nph.17003.

2.Plackova K, Bures P, Zedek F. Centromere size scales with genome size across Eukaryotes. Sci Rep 2021, 11(1):19811. https://doi.org/10.1038/s41598-021-99386-7.

Comment 3: For the figure5, it's better to perform this analysis among more species.

Response: Thank you for your constructive suggestions. We have addressed your concern by including additional species with T2T genomes in our analysis. Specifically, we added lettuce, rice, and grape (Supplementary Fig. S8, Supplementary Tables S16-18, line 288, page 14) to broaden the scope of our comparison. Our findings reveal that the correlations between chromosome length, number and length of centromere repeats (CRs), and centromere length in lettuce and rice are similar to those observed in sandalwood. However, we found that the coverage of CRs is negatively correlated with centromere length in these species, which contrasts with our observations in sandalwood.

Additionally, we noted that no prominent CRs were detected in grape centromeres, which made it difficult for visualization. Consequently, grape were excluded from Supplementary Fig. S8.

These results further highlight the species-specificity of sandalwood centromere composition. We have revised the results section to reflect these updates and provide a clearer understanding of the comparative analysis. The revised section now reads as follows:

“Statistics and analysis of centromere sequences in lettuce and rice showed that the correlations among chromosome length, number and length of CRs, and centromere length were similar to those in sandalwood. However, in contrast to sandalwood, the coverage of CRs was negatively correlated with centromere length, indicating that the centromere composition of sandalwood was species-specific and differed from that of the other species (Supplementary Fig. S8).”

Other comments and questions

For annotation methods, the detailed parameters should be introduced.

Response: The parameters for repeats and gene annotation were added (line 417, page 20; line 419, page 20; line 425, page 21).

Line 60, The typeface of kiwifruit was different from all other words.

Response: We corrected (line 63, page 4).

Line 97, Add blank between furniture and [21].

Response: We corrected (line 99, page 6).

Powered by Editorial Manager® and ProduXion Manager® from Aries Systems Corporation

	Line 101, sequence should be sequences.<br>Response: We corrected (line 103, page 6).<br><br>Line 102, assembly should be assemblies.<br>Response: We corrected (line 104, page 6).<br><br>Line 136, to should be onto.<br>Response: We corrected (line 139, page 8).<br><br>Line 166, sequence should be sequences.<br>Response: We corrected (line 190, page 10).<br><br>Line 309, between should be among.<br>Response: We corrected (line 335, page 16).<br><br>Line 320, CDS should be defined. In first time.<br>Response: Thank you. CDS has been defined in line 298, page 15.
Additional Information:	
Question	Response
Are you submitting this manuscript to a special series or article collection?	No
**Experimental design and statistics**<br><br>Full details of the experimental design and statistical methods used should be given in the Methods section, as detailed in our Minimum Standards Reporting Checklist. Information essential to interpreting the data presented should be made available in the figure legends.<br><br>Have you included all the information requested in your manuscript?	Yes
**Resources**<br><br>A description of all resources used, including antibodies, cell lines, animals and software tools, with enough information to allow them to be uniquely identified, should be included in the Methods section. Authors are strongly encouraged to cite Research Resource Identifiers (RRIDs) for antibodies, model organisms and tools, where possible.<br><br>Have you included the information requested as detailed in our Minimum	Yes

Standards Reporting Checklist?	
**Availability of data and materials**<br><br>All datasets and code on which the conclusions of the paper rely must be either included in your submission or deposited in publicly available repositories (where available and ethically appropriate), referencing such data using a unique identifier in the references and in the "Availability of Data and Materials" section of your manuscript.<br><br>Have you have met the above requirement as detailed in our Minimum Standards Reporting Checklist?	Yes

Manuscript

Click here to access/download;Manuscript;manuscript_final.docx

The telomere-to-telomere (T2T) genome provides insights into the evolution of specialized centromere sequences in sandalwood

Dan Peng[1,2*], Zhou Hong[3*], Shenglong Kan[4], Zhiqiang Wu[1#], Xuezhu Liao[1#]

1. Shenzhen Branch, Guangdong Laboratory for Lingnan Modern Agriculture, Key Laboratory of Synthetic Biology, Ministry of Agriculture and Rural Affairs, Agricultural Genomics Institute at Shenzhen, Chinese Academy of Agricultural Sciences, 518120, Shenzhen, China.
2. Center for Genomics and Biotechnology, Haixia Institute of Science and Technology, Fujian Agriculture and Forestry University, 350002, Fuzhou, China.
3. Research Institute of Tropical Forestry, Chinese Academy of Forestry, 510520, Guangzhou, China.
4. Marine College, Shandong University, 264209, Weihai, China

*These authors contributed equally: Dan Peng, Zhou Hong.

#Corresponding author: Xuezhu Liao (liaoxuezhu@caas.cn), Zhiqiang Wu (wuzhiqiang@caas.cn).

ORCID: Dan Peng [0009-0007-6164-4132]; Zhou Hong [0000-0001-9955-9469]; Shenglong Kan [0000-0001-5396-627X]; Zhiqiang Wu [0000-0002-4238-7317]; Xuezhu Liao [0000-0003-4599-5809].

Abstract

Background

Sandalwood, a prized hemiparasitic plant, is highly sought in the commercial market because of its aromatic core materia. The structure and stability of the genome are instrumental in the rapid adaptation of parasitic plants to their surroundings. However, there is a conspicuous lack of research on the genomic-level adaptive evolution of sandalwood.

Results

In this study, we assembled a gap-free telomere-to-telomere (T2T) reference genome for *Santalum album* using PacBio HiFi, Hi-C, and ultra-long ONT data. The T2T reference genome (Sal_t2t) encompassed annotations of 24,171 genes and 25.34% repetitive sequences, in addition to all 10 centromeres and 20 telomeres across the 10 chromosomes. The results revealed that the three distinct parasitic species of Santalales had diverse centromeric compositions. The *Copia*-type LTR transposon emerged as the most significant in the *S. album* genome, constituting the primary sequence of the centromere and influencing gene expression. Third, in sandalwood, the presence of *Copia* affected the size of the centromeres, and consequently, the genome size. Identification of the sandalwood T2T genome in this study also enabled the identification of more precise organelle transfer fragments.

Conclusions

Our research provides a sandalwood T2T genome, laying the groundwork for future investigations on the evolution of energy organs in parasitic plants. Moreover, it offers

novel insights into the function and evolution of centromeres, as well as the mechanisms of adaptation and parasitism.

Keywords

T2T genome; Centromere; Hemiparasitic species; Cyto-nuclear transfer

Introduction

With the advent of long-read-length sequencing technologies and improved algorithms, genome assembly has entered a new era of telomere-to-telomere (T2T) assembly [1-3]. Compared to genomes with gaps, T2T genomes consist of minimal or no unassembled regions. They contain comprehensive information about telomeres, centromeres, rDNA, complex chromosomal regions, and intracellular gene transfer (IGT) [4]. These areas are often challenging to handle, and complete assemblies may offer a deeper understanding of the structure and function of these unassembled regions. The T2T genome of *Arabidopsis thaliana* reported in 2021 facilitated the exploration of its centromeric genetic and epigenetic characteristics, revealed the mechanism of centromere evolution driven by the homologation of satellite sequences and retrotransposons, and marked the first application of T2T assembly technology in plants [5]. Since then, the sequence composition and evolution of centromeres have been elucidated by T2T genomes in several species, including rice, kiwifruit, watermelon, grape, carnation, and *Peucedanum praeruptorum*, among others [6-11]. However, the number of T2T genomes published to date is limited.

The centromere is a vital structure in eukaryotic chromosomes and plays an indispensable role in cell division [12]. Centromere dysfunction often leads to incorrect chromosome segregation during cell division, which can affect growth and development[13]. For instance, in plants, abnormal centromere function can result in stunted growth and development [14]. The structural and functional elucidation of the centromere is not only a fundamental scientific issue in the field of chromosome biology,

but also a cornerstone for the advancement of synthetic biology. Studies on the composition, structure, and evolution of centromeric sequences are key to elucidating their functions. However, the high number of repetitive sequences in the centromere presents a challenge for precise assembly and functional resolution. Generally, plant centromeric DNA sequences comprise three types: tandem repeats (TR), centromeric retrotransposons (CR), and a few functional genes with transcriptional activity [9, 15, 16]. CRs are typically interspersed with TRs and are abundant in plant centromeric regions, which are depicted as blank regions in the Hi-C contact heatmaps. Therefore, the T2T genome provides a more accurate sequence foundation for the identification of these signature sequences. However, most studies have been confined to crops or widely recognised horticultural plants, with few reports on the sequence characteristics of the centromeres of certain plant taxa such as parasitic plants. In contrast, maintaining chromosomal stability in parasitic plants is essential for their survival and reproduction, as they may need to adapt rapidly to changes in the host plant or environment. For instance, in the parasitic genus *Cuscuta*, the form of the centromeres is believed to be associated with its genome size and chromosome base, with monocentric *Cuscuta* species having a 102-fold variation in genome size and holocentric species having moderately sized genomes [17]. Moreover, to accommodate their parasitic lifestyle, the organelles of the parasitic plant may have undergone specific adaptive modifications, particularly cytonuclear interactions or transfers [18]. Therefore, the complete genomes of parasitic plants are required for relevant studies.

Santalum album, also known as sandalwood, is a valuable hemiparasitic plant

belonging to Santalaceae. Sandalwood, an evergreen tree found across Southeast Asia, Australia, and the Pacific islands, is known for its medicinal properties, such as antimicrobial, antioxidant, and anti-inflammatory effects, and is commercially prized for its aromatic core materials [19, 20]. Its essential oil, termed "liquid gold", is used in perfumes, cosmetics, and incense. Its hard texture and beautiful grains also make sandalwood ideal for carving and furniture [21]. Interestingly, Santalales not only encompasses hemiparasitic plants, such as sandalwood, but also includes non-parasitic (*Malania oleifera*), holoparasitic (*Balanophora subcupularis*), and other hemiparasitic species (*Taxillus chinensis*) [22-24]. Parasitic plants typically display greater genomic sequences and structural differences than non-parasitic plants, as an adaptation to their environment [25]. Previous studies have reported the assemblies of two chromosome/contig-level genomes with dozens to hundreds of gaps for sandalwood [26, 27]. Thus, there remains a research gap regarding the sequence differences between parasitic and non-parasitic plants in Santalales. Therefore, it is imperative to obtain a high-quality T2T genome for further investigation of unknown information in sandalwood.

In this study, we successfully obtained the T2T genome of *S. album*, with a size of 218.90 megabase pairs (Mb), comprising 10 chromosomes and no gaps. We identified all the centromeric and telomere sequences across the chromosomes. Our findings revealed that three species in Santalales with different parasitic forms exhibited differences in centromere composition and that *Copia*-type LTRs significantly influenced *S. album* gene expression. In summary, this study represents the first T2T

assembly of the *S. album* genome and provides an opportunity to investigate the genome structure and function of Santalales species.

Result

The gap-free genome assembly, completeness evaluation and annotation for *S. album*

By integrating 19.91 gigabase pairs (Gb, ~90 x coverage) of Hifi reads, 98.13 Gb (~430 x coverage) of Hi-C data, and 35.02 Gb (~160 x coverage) of ultra-long ONT reads, we obtained a gap-free sandalwood genome (named Sal_t2t) with a size of 218.90 Mb, comprising 10 chromosomes. The longest chromosome measures 34.97 Mb, and the shortest is 14.10 Mb (Fig. 1A, Table 1 and Supplementary Table S1). Compared with the two previously published versions of the sandalwood genome (V1: 23 gaps; V2: 108 gaps), all gaps in Sal_t2t were filled. Additionally, all SVs and PAVs that differed from the previous two assemblies in complex regions or diversity between haplotypes, were supported by more than three long reads (Hifi or ultra-long ONT reads) in the Sal_t2t genome (Fig. 1 C-E, Supplementary Fig. S1). The contig N50 length of the Sal_t2t genome (18.40 Mb) is 1.44-3.31 times greater than that of V1 (12.75 Mb) and V2 (5.56 Mb) genomes, demonstrating a significant improvement in continuity and completeness in the newly assembled Sal_t2t (Table 1).

Assembly accuracy was further assessed using several methods. First, the Benchmarking Universal Single-Copy Orthologs (BUSCO) assessment based on embryophyta_odb10 revealed that 98.3% of the core conserved plant genes (1,586/1,614 genes) were fully characterised in this genome. Second, the comparison

showed that 99.65% of short reads, 99.97% of HiFi reads, and 96.12% of RNA-Seq data from leaf samples could be mapped onto the Sal_t2t genome. Furthermore, integrity checks of the long terminal repeats (LTRs) revealed an assembled LTR assembly index (LAI) of 26.41. This genome has a consensus quality value (QV) of 67.30. Collectively, these data demonstrated the high accuracy of the Sal_t2t assembly (Table 1).

The Sal_t2t genome provides an unprecedented opportunity to identify all the repeat sequences and genes. Consistent with previous predictions, 25.34% (55.48 Mb) of the sequences were identified as transposable elements (TEs), with 19.54 Mb (8.93%) of retrotransposons and 23.04 Mb (10.51%) of DNA transposons in Sal_t2t (Fig. 1F and Supplementary Table S2). Retrotransposons and DNA transposons were predominantly distributed in the central regions of the chromosomes (Fig. 1B). We further predicted 24,171 protein-coding genes, 12 of which were either pseudogenes or incomplete (Table 1 and Supplementary Table S3). Moreover, the completeness of the annotations was assessed using BUSCO, revealing that approximately 98.3% and 96.0% of the core genes in the assembly and gene set were complete, which was much higher than those of the V1 (98.2% and 93.5 %) and V2 (98.0% and 89.6 %) versions (Fig. 1G and Table 1). A total of 567 tRNA, 332 snRNA, and 3,910 rRNA sequences were annotated and predicted in the sandalwood genome using the Rfam database (Table 1 and Supplementary Table S4). Among these, 3,324 were annotated as 5S rRNA, accounting for 85.01% of the total rRNA count, primarily distributed in the genomic region of 21.72-23.38 Mb on Chr1.

Identification of new genes

To test whether different assembly methods selectively enrich certain types of genes, we analysed the differences between the annotation results of the two versions, Sal_t2t (Sal_ano) and V1 (V1_ano). Using Liftoff software with Sal_ano and V1_ano as references, 21,583 and 24,007 genes were annotated to the genomes of V1 and Sal_t2t, respectively. Sal_ano contained 1,282 previously unannotated genes, while V1 contained 2,583 genes, which may be due to differences in annotation software and parameters. In addition, the *de novo* Sal_t2t genome assembled 164 genes, whereas V1 had 92 genes, which were identified as new genes based on Liftoff software (Supplementary Table S5).

In addition, the 164 new genes identified in Sal_t2t were mainly enriched in the GO terms oxidoreductase activity, biosynthetic processes, and metabolic processes (Supplementary Fig. S2). Whereas 29 of these 164 new genes were located in regions located in the filled gaps of V1, and 28 of these new genes were located in the filled regions enriched with rRNAs and TRs in Sal_t2t. These genes showed high similarity and were supported by ultra-long ONT reads, suggesting that they may be duplicated. Classification of all new genes by DupGen_finder software revealed 152 (92.68%) new genes were categorized (dispersed/proximal/tandem/transposed/wgd: 26/60/36/12/18), and the proportion of duplicated genes in the genome-wide background was 70.84%. Moreover, 56 duplicate genes (dispersed/proximal/tandem/transposed/wgd: 4/18/18/5/11) were expressed (Supplementary Table S6).

Organelle gene transfer

The integrity of organelle transfer fragments, such as nuclear integrants of mitochondrial DNA (NUMTs) and nuclear integrants of plastid DNA (NUPTs), also serves as an index for evaluating assembly quality [28]. By assembling the nuclear, mitochondrial, and chloroplast genomes of *S. album*, we evaluated the frequency and patterns of NUPTs and NUMTs among the Sal_t2t, V1, and V2 genomes and found similar quantity and length distribution patterns. However, more accurate organelle transfer fragments were identified in Sal_t2t than in the other two versions, especially in the intergenic region (IGR) (Fig. 2A, B and Supplementary Table S7).

In comparison with the ultra-long ONT sequences, we also identified a mis-assembly of the nuclear genome involving five NUPTs larger than 10 kilobase pairs (Kb), which was corrected for Sal_t2t (Supplementary Fig. S3). Additionally, we observed that regions where the same chloroplast fragment was transferred multiple times to the nuclear genome might also have been misassembled, including redundancy in low-copy chloroplast transfer fragments and partial or complete loss of multi-copy chloroplast transfer fragments (Supplementary Tables S8, S9). Sequence alignment verification results also demonstrated the integrity and accuracy of the transfer fragment assembly in the T2T genome (Fig. 2C-F).

We also examined the quantity of transposons upstream and downstream of the organelle transfer fragments and found that the V2 and V1 genomes had fewer transposable elements surrounding the organelle transfer fragments, whereas Sal_t2t had the highest number of intact TEs (Fig. 2G and Supplementary Fig. S4). Further categorisation revealed that the quantities of LTR, MITE, TIR, LINE, and Helitron

transposons were similar among the three versions, but there were significant differences between DNA transposons and unknown transposons (Supplementary Fig. S5). In DNA transposons, the average content was the highest in Sal_t2t and lowest in V2, whereas the trend was reversed in unknown transposons (Fig. 2H). This indicates that the Sal_t2t genome provides a clearer and more accurate prediction of the distribution, types, and quantities of TEs surrounding the transfer fragments.

Architecture and context of telomeres and centromeres

The completion and accuracy of genome sequencing have enabled the identification of telomeres and centromeres. First, the results of telomeric regions revealed that both ends of the 10 sandalwood chromosomes possessed telomere repeat units (AAACCCT/AGGGTTT), aligning with the telomere structure of most plants. The longest telomere, located on chromosome 1, measured 16.62 Kb and contained 2,374 repeats, while the shortest telomere, found on chromosome 9, measured 1,869 base pairs (bp) with only 267 repeats (Supplementary Tables S10).

In addition, we identified centromeric regions using quarTeT, combined with EDTA annotations and the blank regions in the Hi-C contact matrices as candidate centromeric regions (Fig. 3A and B). We quantified and visualised TEs in each chromosome's candidate regions and observed that three *Copia* repeats (TE_00001095, TE_00001228, and TE_00001258) and one unknown LTR repeat (TE_00000831) were highly enriched in eight candidate regions and two secondary candidate regions across chromosomes, whereas they were scarce on chromosome arms (Fig. 3C). We speculated that these four LTR sequences were CRs.

Second, 470 distinct TR units were identified in the Sal_t2t genome. A 312 bp repeat was the most abundant unit in the genome, with a total of 10,335 copies of ⩾2 repeats, accounting for 1.47% of the entire genome sequence. This was followed by 500 bp (0.72%), 32 bp (0.42%), and 63 bp (0.30%). However, the top 20 units in the genome, in terms of total length and total copy number, lacked typical centromeric TR sequence distribution characteristics (Supplementary Fig. S6). Subsequently, 21 types of high-order repeat (HORs) regions were identified, among which SR3 (prefix#circ3-7198) was primarily composed of two tandem repeats of 20 base pairs in length (AGCCCAAGCACACTTGGAGG and TCCAAGTGTCATTGGGCTCC), which highly overlapped with the candidate regions and CRs (Fig. 2D-E and Supplementary Table S11). Therefore, we defined the distribution ranges of the CR and TR (from the SRF results) as the centromeric regions of all chromosomes (Supplementary Table S12).

Comparative analysis of centromeric sequences in Santalales

To study the differences and evolution of centromere sequences among various species of Santalales, we identified and analysed the centromere sequences of available genomes of Santalales species. We focused on the CRs and TRs of *S. album*, *M. oleifera*, and *T. chinensis*. The genomes of *Balanophora* and *Scurrula* were excluded from the analysis because of the low quality of their assembly.

In the Sal_t2t genome, the TRs consisted of two 20 bp sequences, a pattern similar to that observed in the V2 genome. These 20 bp sequences were found in low numbers in the centromeric region, with only two copies per repeat unit. The number of repeat units per chromosome ranged from 2 (Chr9) to 46 (Chr4), averaging 19.5 units per

chromosome. We identified TRs in the same pipeline for the other two species and found that the length (259 and 260 bp) and copy number (101 - 223) of the TR repeat units in *T. chinensis* were significantly higher than those in the sandalwood genome. In *M. oleifera*, centromeric tandem repeats primarily consisted of 66 bp repeat sequences, with copy numbers ranging from 10 to 110 (Fig. 4A).

In terms of centromeric retrotransposon sequences, we identified three *Copia* sequences and one unknown LTR sequence in Sal_t2t, with one-quarter of the TE_00001095 sequence being intact LTRs. However, in *T. chinensis*, we identified only one unknown LTR sequence, whereas in *M. oleifera*, we identified one *Copia* sequence and one *Gypsy* sequence. We utilised these CRs to construct phylogenetic trees and observed that the LTR sequence TE_00001228 of sandalwood clustered with several LTR sequences of *T. chinensis* and *Copia* sequences of *M. oleifera* in the same clade, whereas the LTR sequence TE_00000831 of sandalwood belonged to the same clade as the majority of the LTR sequences of *T. chinensis* (Fig. 4B). Additionally, the complete LTR sequence TE_00001095 of sandalwood did not cluster with *T. chinensis* or *M. oleifera*, whereas the majority of *Copia* and *Gypsy* sequences of *M. oleifera* clustered separately into distinct clades. Furthermore, differentiation occurred in the same LTR classification in the same species, as evidenced by the differences in CR and TR content in these four species (Fig. 4C). These findings suggest that while centromeric TR and CR sequences are conserved in species, they differ significantly between species. However, owing to limitations in the quantity and quality of published genome assemblies, further analysis and comparison of telomeric regions may require

additional T2T genome sequences from the same family or genus. This could be particularly relevant for assessing the conservation of the CR and TR sequences at different taxonomic levels.

Relationship between centromere and chromosome characteristics

To explore the correlation between the centromere and the evolution of chromosome length, we compared the composition of sequences on chromosomes. The distribution of transposons across the entire chromosome indicated a high proportion of *Copia* transposons significantly enriched in the centromeric region (>30% of the total length; *chi*-test, $p < 0.01$) throughout the genome. Several complex regions exhibited high DNA transposon enrichment (Fig. 5A, B and Supplementary Fig. S7).

We further analysed the quantity and length of *Copia* and the correlation between centromere length, and chromosome length and revealed a correlation between centromere length and chromosome length (R^2 = 0.4008), although this was not statistically significant (p = 0.1665). Subsequently, we found that chromosomes with longer centromeres had more and longer *Copia* elements (R^2 = 0.6114, $p < 0.05$) and centromeres *Copia* (R^2 = 0.9945, $p < 0.01$) (Fig. 5C). Therefore, we speculated that the enrichment of *Copia* might lead to a positive association between centromere and chromosome lengths in Sal_t2t, and this pattern might be diluted by the low-density of *Copia* in the chromosome arms (Fig. 5C). Statistics and analysis of centromere sequences in lettuce and rice showed that the correlations among chromosome length, number and length of CRs, and centromere length were similar to those in sandalwood. However, in contrast to sandalwood, the coverage of CRs was negatively correlated

with centromere length, indicating that the centromere composition of sandalwood was species-specific and differed from that of the other species (Supplementary Fig. S8).

Given that TE insertion can influence gene expression, we also investigated TE insertion and expression levels in the centromeric and other regions. The results revealed that TE insertions occurred in all 165 centromeric genes in their 2 Kb flanking regions, with 92.73% (153/165) of centromeric genes containing TEs in introns, and 89.09% (147/165) of genes overlapping between TEs and coding sequences (CDS) (Fig. 5D). These proportions were significantly higher than those of genes in other genomic regions (74.05%, 34.18% and 5.65% of genes containing TEs in 2 Kb flanking regions, introns, and CDS, respectively), and centromeric genes exhibited significantly lower expression compared to other genes in the genome (*wilcox* test, $p < 0.01$) (Fig. 5E and Supplementary Fig. S9). Additionally, most of these genes could not be annotated using the NR and Swiss-Prot databases. Among the few genes identified, only partial fragments were functionally annotated (Supplementary Table S13). This indicates that these genes underwent rapid mutations, which may be related to the high density of *Copia* insertions.

Furthermore, we explored the effect of TE insertions on gene expression across all genes. We found that genes containing *Copia* insertions exhibited significantly lower expression levels than other types of TE insertions at the genome-wide level, with *Copia* insertions in the CDS regions resulting in the greatest reduction in gene expression (Fig. 5F). Importantly, we found that *Copia* insertion into the intronic regions significantly reduced gene expression in the sucker, a specialised organ of

sandalwood, compared to expression in roots, whereas insertion into the gene flanking region was more strongly inhibited in the root, and there was no significant difference when inserted into the CDS region, suggesting a potential effect on organ differentiation (Supplementary Fig. S10).

Discussion

Centromeres play a crucial role in maintaining genome stability in eukaryotes, shaping genome structure and driving karyotype evolution[29, 30]. However, in plants, the evolutionary relationship between centromere structure and function remains unclear. This is because the centromeric sequences themselves do not encode proteins and lack evolutionary dependence despite their high diversity among closely related species, and the highly conserved function of the centromere [31, 32]. In this study, we identified the sandalwood-specific centromere sequence composition and its impact on genome length and gene expression by assembling a sandalwood T2T genome. We compared the sequence compositions of different species in Santalales and found that although the centromere TR sequences were conserved, there were significant differences among the species, even in the case of conspecific hemiparasitism. Moreover, differentiation occurred even within the same LTR classification for the same species, as evidenced by differences in the CR and TR contents of these three species.

Three types of centromere sequence compositions have been reported in many species [30, 31]. For instance, in grapes, a 107 bp repeat sequence serves as a centromere signature sequence and is highly conserved among chromosomes [33]. In

addition, most TEs in plant centromeres are composed of LTR-type *Gypsy*-like retrotransposons [34, 35]. For example, in rice, *Gypsy* plays a pivotal role in the formation and evolution of the centromere, particularly in young *Gypsy* LTRs [36]. In cotton (*Gossypium hirsutum*), a similar situation was observed that unclassified LTRs and *Gypsy*-type LTRs were the primary components of centromeric regions, and *Gypsy* contributed to the centromere evolution compared to *Copia* [34]. In our study, we found that the type of centromere-specific LTR was mainly *Copia*, with a higher frequency distribution in the centromeres, similar to that in *Brassica oleracea* [37]. Moreover, the presence of *Copia* significantly reduced gene expression, and the insertion of *Copia* into CDS, introns and flanking regions of genes significantly reduced gene expression in roots and suckers. For genes with *Copia* insertions in the intron, genes with lower expression in the sucker relative to the root were enriched for pathways such as biosynthesis and metabolism, suggesting that the expression of genes related to metabolic pathways was suppressed in the sucker, whereas basic nutrient uptake functions might be retained (Supplementary Table S14 and S15). This might be an adaptive and survival strategy for sandalwood that reduces the production of unwanted secondary metabolites in the sucker, thus optimising resource utilisation for better uptake of nutrients from the host. Therefore, this particular *Copia* composition may be related to sandalwood parasitism.

In addition, some researchers have proposed that parasitism might lead to an increase in genome size [17, 38] because the parasitised plant is liberated from the limitations of the growth rate of the root meristematic tissue, or the resources obtained

from the host. However, this was subsequently refuted in studies of the genus *Cuscuta*, which nonetheless found a correlation between genome size changes and the centromeric form, whereas no association was found between parasitic forms and genome size. For instance, species with monocentric chromosomes exhibit a 102-fold variation in genome size and a higher basic chromosome number, whereas species with holocentric chromosomes have modest genome sizes [17]. We also found that parasitism did not lead to genome expansion in sandalwood. The genome size of the non-parasitized *M. oleifera* was 1.5 Gb, whereas that of the hemiparasitic species *T. chinensis* was 521.90 Mb, and the genome size of hemiparasitic species *S. album* was 218.90 Mb. However, we found that *Copia* was positively correlated with the genome size. We found that chromosomes with longer centromeres exhibited more *Copia* and centromere-specific *Copia*. This suggest that *Copia* content promoted the length expansion of both centromeres and chromosomes.

In conclusion, we constructed the first T2T genome of sandalwood by combining HiFi, Hi-C, and ultra-long ONT data. We resolved the sequence composition and function of telomeres and centromeres and provided new insights into the genome evolution of parasitic plants.

Methods

Plant materials and genome sequencing

Genomic DNA was extracted from the leaves collected at the Experimental Station of the Research Institute of Tropical Forestry, Chinese Academy of Forestry, Guangzhou, China. The extracted DNA was assessed for concentration and quality

using NanoDrop 2000 and used to construct linked read libraries using a PacBio SMRTbell library from an SMRTbell Prep Kit 3.0 (PN: 102-182-700), following the manufacturer's protocols and then sequenced on the PacBio Revio platform (PacBio Sequel II System, RRID:SCR_017990) for generating HiFi reads. For ONT ultra-long sequencing, a standard library was prepared using the SQK-LSK109 kit, following the standard protocol. The purified library was sequenced using a PromethION sequencer (Oxford Nanopore Technologies; RRID:SCR_017987). For RNA-seq, phenol/chloroform was used to isolate RNA from root, sucker, stem, and leaf samples (three bioreplicates), which were checked for purity and integrity before construction. Libraries for all four tissues were prepared using mRNA-seq preparation kits and sequenced in PE150 mode on the MGISEQ-2000 platform.

Genome assembly and quality evaluation

De novo assembly was performed using Hifiasm (v0.19.6-r595) (RRID:SCR_021069) [39]. For Hi-C sequence data [26], Juicer (v1.6) (RRID:SCR_017226) [40] and bowtie2 (v2.3.2) (RRID:SCR_016368) [41] were used to filter out low-quality, and 3D-DNA (v180922) (3D de novo assembly, RRID:SCR_017227) [42] was used to unvalidated paired-end reads and construct interaction matrices to obtain chromosome-scale genomes. The redundant contigs were removed using Purge_Haplotigs (v1.1.2) (RRID:SCR_017616) [43]. The draft genome was then subjected to a final round of gap filling using ONT Ultra-long reads corrected by NextDenovo (v2.17-r941) (RRID:SCR_025033) [44] with LR_Gapcloser (v1.9.4) (RRID:SCR_016194) [45] and Minimap2 (v2.24-r1122) (RRID:SCR_018550) [46] to

obtain a T2T genome.

The completeness of the assembled Sal_t2t genome sequences was analyzed using BUSCO (v5.3.2) (RRID:SCR_015008) [47] with the embryophyta_odb10 databases (issued 2020-08-05, including 1614 proteins), and the LAI statistic in LTR_retriever (v2.9.0) (RRID:SCR_017623) [48] with default parameters based on intermediate results from the EDTA pipeline (v1.9.4) (RRID:SCR_022063) [49]. To measure genome coverage based on read-mapping rates, NGS short reads [26], HiFi reads, and RNA-seq reads were mapped against the assembled genome sequences using BWA-MEM (v0.7.9a, https://github.com/lh3/bwa) (RRID:SCR_022192), minimap2 and HISAT2 (v2.2.1) (RRID:SCR_015530) [50]. The GC content distribution was used to detect sample contamination.

Genome annotation

To identify repeat sequences Sal_t2t and other three Santalales species, several programs in EDTA, including LTR_FINDER (v1.07) (RRID:SCR_015247), LTRharvest (genometools, v1.6.1) (RRID:SCR_018970), LTR_retriever, Generic Repeat Finder (v1.0), HelitronScanner (v1.1), TIR-Learner (v2.5), RepeatMasker (v4.1.1) (RRID:SCR_012954), and RepeatModeler (v2.0.1) (RRID:SCR_015027), as well as a series of integration scripts, were used to annotate and identify LTR, LINE, SINE, Helitron, MITE, and other retrotransposon and transposon sequences, with options "--anno 1 --force 1 --debug 1 -sensitive 1". In addition, we utilized Tandem Repeat Finder (TRF v4.09, http://tandem.bu.edu/trf/trf.html) (RRID:SCR_022193) [51] with the parameters "2 7 7 80 10 50 500 -f -d -m" to independently predict tandem repeats in the genome.

To annotate the gene structure, we used the GETA pipeline (v2.5.1, https://github.com/chenlianfu/geta) with three methods: homology, *de novo*, and transcript-based annotation. Published protein information for *Vitis vinifera*, *Arabidopsis thaliana*, *M. oleifera*, *S. yasi* and V1 genomes was used as a homology reference. The parameters "[hisat2] --min-intronlen 20 --max-intronlen 20000 --dta --score-min L,0.0,-0.4, [sam2transfrag] --fraction 0.05 --min_expressed_base_depth 2 --max_expressed_base_depth 50 --min_junction_depth 2 --max_junction_depth 50 --min_fragment_count_per_transfrags 10 --min_intron_length 20, [TransDecoder.LongOrfs] -m 100 -G universal, [homolog_genewise] --coverage_ratio 0.4 --evalue 1e-9, [homolog_genewiseGFF2GFF3] --min_score 15 --gene_prefix genewise –filterMiddleStopCodon, [geneModels2AugusutsTrainingInput] --min_evalue 1e-9 --min_identity 0.8 --min_coverage_ratio 0.8 --min_cds_num 2 --min_cds_length 450 --min_cds_exon_ratio 0.60, [prepareAugusutusHints] --margin 20, [paraAugusutusWithHints] --gene_prefix augustus --min_intron_len 20, [paraCombineGeneModels] --overlap 30 --min_augustus_transcriptSupport_percentage 10.0 --min_augustus_intronSupport_number 1 --min_augustus_intronSupport_ratio 0.01, [pickout_better_geneModels_from_evidence] --overlap_ratio 0.2 --ratio1 2 --ratio2 1.5 --ratio3 0.85 --ratio4 0.85, [PfamValidateABinitio] --CDS_length 750 --CDS_num 2 --evalue 1e-5 --coverage 0.25, [remove_genes_in_repeats] --ratio 0.8" were used in GETA pipline.

Finally, we used CMSScan (v1.1.4, https://github.com/ajinabraham/CMSScan) to mine ncRNA information using the Rfam non-redundant database, which is based on the homology annotation of ncRNAs, including tRNA, rRNA, miRNA, and snRNA.

These results were visualized using the Integrative Genomics Viewer (IGV, v.2.12.3) (RRID:SCR_011793) (Supplementary Fig. S11).

Genome comparison

V1 assembled genome, and *M. oleifera* genome were downloaded from the CNCB under accession number PRJCA009490 and PRJNA472200. V2 assembled genome and annotation were downloaded from the Figshare database [52]. *T. chinensis* genome was downlowd from NCBI under PRJNA855314.

The Synteny and Rearrangement Identifier (SyRI v1.5.4) (RRID:SCR_023008) [53] was utilized to detect collinearity, SVs, and PAVs among the three versions of the sandalwood genomes. ONT ultra-long and HiFi reads were used to validate the accuracy of the assembly of Sal_t2t with read mapping.

Identification of new genes

We used Liftoff (v1.6.3) [54] with default options to identify new genes. The homologous annotation results were compared with annotation files using the BEDtools (v2.30.0) (RRID:SCR_006646) [55] and the AWK command. Genes that were newly annotated in the homologous annotations but not in the annotation files were considered new results because of the differences in software use. The genes in the unmapped files were new genes.

Organelle genome assembly

GSAT (v1.11) [56] was used to assemble the mitochondrial genome with 4 Gb Illumina reads and all HiFi reads. SPAdes (v3.15.5) (RRID:SCR_000131) [57] was used to assemble the chloroplast genome with 5 Gb Illumina reads. The complete CP

and MT assemblies were visualised with Bandage (v0.9.0) (RRID:SCR_022772) [58] to remove contigs with abnormal coverage and simplify the genome using the organelle genomes downloaded from NCBI (NC_081498.1 and NC_048953.1) as a reference.

Organelle gene transfer

Based on the assembly and annotation files of the *S. album* nuclear genome, Blastn (v0.8.1) (RRID:SCR_001598) software was used to identify transfer events from organelle to nuclear genomes with default parameters. We filtered the transfer fragments less than 30bp in length and the identify score less than 80%. We extracted the 500bp,1000bp and 2000bp upstream and downstream of the transferred fragments for TE statistics combined with the EDTA annotation results. These results were visualized using the ggplot2 package (RRID:SCR_014601) in R [59].

Identification of telomeres and centromeres

Referring to the research methods used in the grape (PN40024) genome [9], we used TIDK (v.0.2.0) [60], TRF for the identification of centromeres and telomeres, and combined with the result from quarTeT (v1.1.4) (RRID:SCR_025258) [61], EDTA pipeline and srf [62] as a complement. The telomere repeat units were explored by TIDK with options "tidk explore -f genome.fa -minimum 5 -maximum 12 -o tidk_explore -t 2 -log -dir telomere_find -extension TSV". Then the whole genome was searched using the following parameters "tidk search -f genome.fa -s AAACCCT -o tidk_search -dir telomere_find".

For centromere annotation, we used the candidate regions identified by quarTeT and the blank regions in Hi-C contact matrices referring to the method for the faba

genome [63], extracted all TE sequences presented in the candidate centromeric regions and calculated the length and count of these TEs. We selected the top 10 TEs and found that three *Copia* repeats (TE_00001095, TE_00001228, and TE_00001258) and one unknown LTR repeat (TE_00000831) were mainly enriched in the candidate regions. TRF was used to scan tandem repeats ranging from 30 to 500 base pairs in the genome, and then we merged the results of annotation using trf2gff in TRF. We visualised the top 30 repetitive sequences in terms of total count and length using the ggplot2 package in R but could not find the enrichment of tandem repeats in most centromeres. We then used SRF to identify HORs and found only one HOR located on all chromosomes. Combined with the TRF results, this HOR was found to be composed of two 20 bp tandem repeat sequences and was considered to be a characteristic of centromeric satellites. To complete the data statistics and visualisation, we used information from the TRF, SRF, and EDTA results extracted by the AWK command in the Linux system and analysed the results using the IGV. Fitting and visualisation were implemented using the ggplot2 package in R. We considered the intersection region of the TRs (from SRF) and TEs (from the EDTA pipeline and TIDK) to be the centromere region.

Candidate centromeres in *M. oleifera*, *T. chinensis*, V2, rice and lettuce [64] were identified using the method described above (Supplementary Tables S16-18).

Evolution analysis of CRs in Santalales

We used BEDTools to extracted the CRs and aligned using MAFFT (v7.480) (RRID:SCR_011811) [65]. Then we used BMGE (v1.12) [66] to remove ambiguously aligned regions with options “-g 0.85 -h 1 -b 1 -w 1”. We constructed an ML tree using

FastTree (v2.1.10) (RRID:SCR_015501) [67] with a GTR model. Finally, the tree was adjusted, customized, and displayed using iTOL (RRID:SCR_018174) [68].

Data Availability

The nuclear genome assembly and all sequencing data including Hifi, ONT, and RNA-seq have been submitted to the NCBI under BioProject number PRJNA1127301. Gene annotations, chloroplast genome, and mitochondrial genome have been deposited to figshare[69]. All supporting data and materials are available in the *GigaScience* GigaDB database [70].

Abbreviations

BUSCO: Benchmarking Universal Single-Copy Orthologs; bp: base pairs; CDS: coding sequences; CRs: centromeric retrotransposons; Gb: gigabase pairs; Hi-C: High-throughput chromosome conformation capture; HORs: high-order repeats; IGR: intergenic region; IGT: intracellular gene transfer; Kb: kilobase pairs; LAI: LTR assembly index; LTRs: long terminal repeats; Mb: megabase pairs; miRNA: micro RNA; NCBI: National Center for Biotechnology Information; ncRNA: non-coding RNA; NUMTs: nuclear integrants of mitochondrial DNA; NUPTs: nuclear integrants of plastid DNA; ONT: Oxford Nanopore Technologies; PacBio: Pacific Biosciences; QV: quality value; rRNA: ribosomal RNA; SMRT: single-molecule real-time sequencing; snRNA: small nuclear RNA; T2T: telomere-to-telomere; TEs: transposable elements; TR: tandem repeats; tRNA: transfer RNA.

Authors' contributions

Xuezhu Liao, Zhiqiang Wu and Zhou Hong provided the ideas and frame of this paper. Dan Peng performed the bioinformatic analysis and drafted the manuscript with Xuezhu Liao. Dan Peng and Xuezhu Liao revised the manuscript with the help of Zhiqiang Wu and Zhou Hong. Shenglong Kan and Zhou Hong prepared the sequencing samples. All authors read and approved the final manuscript.

Funding

This work was funded by the Guangdong Pearl River Talent Program (grant 2021QN02N792), the Chinese Academy of Agricultural Sciences Elite Youth Program (grant 110243160001007) and the Shenzhen Fundamental Research Program (grant JCYJ20220818103212025).

Competing Interests

The authors declare that they have no competing interests.

Table

Table 1: Comparision of three sandalwood assemblies

	Sal_t2t	V1 (Hong)	V2 (Zhang)
Total length (Mb)	218.90	229.60	207.45
Gaps	0	23	108
Contig N50 (Mb)	18.40	12.75	5.56
BUSCO - genome	C:98.3%[S:96.4%, D:1.9%]	C:98.2%[S:96.3%, D:1.9%]	C:98.0%[S:95.4%, D:2.6%]
RNA reads mapping rate - Leaf	96.12%	96.05%	92.54%
NGS reads mapping rate	99.65%	98.81%	96.93%
HiFi reads mapping rate	99.97%	99.98%	99.48%
TE content	25.34%	28.93%	22.05%
TE content - Copia	6.63%	6.10%	5.73%
TE content - Gypsy	1.50%	1.09%	1.81%
Gene counts	24,171	21,673	23,282
BUSCO - gene set	C:96.0%[S:93.6%, D:2.4%]	C:93.5%[S:91.3%, D:2.2%]	C:89.6%[S:86.9%, D:2.7%]
rRNAs	3,910	7,720	610
tRNAs	567	614	506
Transfer count - mitochondria	2,416	2,400	2,246
Transfer count - chloroplast	2,620	2,515	2,299

Figures

Figure 1: Assembly and annotation of Sal_t2t. (A) Heatmap of genomic interactions of Sal_t2t genome. (B) Characterization of Sal_t2t genome. The density of genes, TEs and GC content were calculated per 200 Kb. (C) Translocations in Chr4 (Sal_t2t) vs

Chr02 (V2). Dots and lines represent chromosomes alignments between Sal_t2t, V1 (red) and V2 (blue). (D) Translocations in Chr10 (Sal_t2t) vs Chr01 (V2). The other contents were the same as panel a. (E) Alignments between Chr3 (Sal_t2t), Chr04 (V1) and Chr03 (V2) from 5Mb to 20 Mb. Deep red thick lines represent the centromere region. (F) Proportion of TE elements in three sandalwood assemblies. (G) BUSCO assessment of genomes and gene sets in three sandalwood assemblies.

Figure 2: Organelle gene transfer. (A) Counts of NUPTs and NUMTs in three sandalwood assemblies. (B) Length of NUPTs and NUMTs in three sandalwood assemblies. (C - F) Simplified comparison of the chloroplast genome and three nuclear genome assemblies: (C) Overall deletion of a neighboring chloroplast fragment transferred multiple times; (D) Partial copy number deletion of a chloroplast fragment transferred to a different chromosome; (E) Partial copy number deletion of a chloroplast fragment transferred to the same chromosome; (F) Complete deletion of a chloroplast fragment transferred to a chromosome. (G) Counts of intact TEs in flanking regions of transfer fragments. (H) Counts of DNA and Unknown type of TEs in flanking regions of transfer fragments.

Figure 3: Characteristics and distribution of repeats in centromeres. (A) Heatmap of genomic interactions of each chromosome in Sal_t2t genome. (B) Best candidate regions predicted by quarTeT. (C) Distribution of CRs. (D) HORs regions predicted using SRF. (E) Distribution of TRs in HORs. (F) The final centromere regions.

Figure 4: Comparison of centromeric repeats in Santalales. (A) Scatter plot of TRs in HORs of four Santalales genomes/assembles. Counts of each TR unit associated with

circle sizes. Shadows in the background represent the roughly distributed areas of TRs in each species. (B) Phylogeny of CRs of four Santalales genomes/assembles without branch length. Different colors in the inner ring represent TE sequences in each species. (C) Proportions of TE and TR in centromere regions of four Santalales genomes/assembles.

Figure 5: Relationship between centromere and chromosome characteristics. (A) Genome-wide fitting curve of TE coverage (windows: 500 Kb). (B) Counts of each TE types in centromere regions. (C) Point plot and linear correlation analysis. * represented $p < 0.05$, ** represented $p < 0.01$. Chr: chromosome. (D) TE insertion statistics in centromeric genes. (E) Comparison of expression among centromere genes and genes in other regions (*wilcox* test, ** represents $p < 0.01$). The circle represents the mean expression and the vertical line represents the standard deviation. (F) Relationship between TE insertion and expression at the genome-wide genes. Genefl, flanking region of genes.

Supplementary Material

Supplementary Fig. S1: Structural variation between three Santalales assemblies.

Supplementary Fig. S2: GO enrichments of new genes and disappeared genes in the Sal_t2t genome.

Supplementary Fig. S3: Density distribution of transfer fragment length of three sandalwood assemblies. Cp: chloroplast genome, Mt: mitochondrial genome.

Supplementary Fig. S4: Counts of TE insertions in different flanking region (bp) of

transfer fragments.

Supplementary Fig. S5: Counts of different types of TE insertions in different flanking regions (bp) of transfer fragments.

Supplementary Fig. S6: Distribution of top 20 TRs in each chromosome.

Supplementary Fig. S7: Count distribution of different type of TEs in each chromosome.

Supplementary Fig. S8: Point plot and linear correlation analysis of rice and lettuce. * represented $p < 0.05$, ** represented $p < 0.01$.

Supplementary Fig. S9: TE insertion statistics in all genes. Genefl, flanking region of genes; CDS: Coding Sequence; Intron: intronic regions.

Supplementary Fig. S10: Relationship between TE insertion and expression in root and sucker. The vertical line represents the standard error. Genefl, flanking region of gene; CDS: Coding Sequence; Intron: intronic region.

Supplementary Fig. S11: Annotations of complex regions were visualized by IGV. and the distribution of genes, TEs (excluding Chr1), all TEs, TRs, 5S rRNAs, and rRNAs other than 5S are shown from top to bottom.

References

1. Li H, Durbin R. Genome assembly in the telomere-to-telomere era. *Nat Rev Genet* 2024. https://doi.org/10.1038/s41576-024-00718-w.
2. Sohn JI, Nam JW. The present and future of de novo whole-genome assembly. *Brief Bioinform* 2018, 19(1):23-40. https://doi.org/10.1093/bib/bbw096.
3. Kong W, Wang Y, Zhang S, et al. Recent advances in assembly of complex plant genomes. *Genom Proteom Bioinf* 2023, 21(3):427-439. https://doi.org/10.1016/j.gpb.2023.04.004.
4. Chen J, Wang ZJ, Tan KW, et al. A complete telomere-to-telomere assembly of the maize genome. *Nat Methods* 2023, 55(7):1221-1231. https://doi.org/10.1038/s41588-023-01419-6.

5. Wang B, Yang X, Jia Y, et al. High-quality *Arabidopsis thaliana* genome assembly with Nanopore and HiFi long reads. *Genom Proteom Bioinf* 2022, 20(1):4-13. https://doi.org/10.1016/j.gpb.2021.08.003.

6. Shang L, He W, Wang T, et al. A complete assembly of the rice Nipponbare reference genome. *Mol Plant* 2023, 16(8):1232-1236. https://doi.org/10.1016/j.molp.2023.08.003.

7. Han X, Zhang YL, Zhang Q, et al. Two haplotype-resolved, gap-free genome assemblies for *Actinidia latifolia* and *Actinidia chinensis* shed light on the regulatory mechanisms of vitamin C and sucrose metabolism in kiwifruit. *Mol Plant* 2023, 16(2):452-470. https://doi.org/10.1016/j.molp.2022.12.022.

8. Deng Y, Liu SC, Zhang YL, et al. A telomere-to-telomere gap-free reference genome of watermelon and its mutation library provide important resources for gene discovery and breeding. *Mol Plant* 2022, 15(8):1268-1284. https://doi.org/10.1016/j.molp.2022.06.010.

9. Shi XY, Cao S, Wang X, et al. The complete reference genome for grapevine (*Vitis vinifera* L.) genetics and breeding. *Hortic Res* 2023, 10(05):uhad061. https://doi.org/10.1093/hr/uhad061.

10. Lan L, Leng L, Liu W, et al. The haplotype-resolved telomere-to-telomere carnation (*Dianthus caryophyllus*) genome reveals the correlation between genome architecture and gene expression. *Hortic Res* 2024, 11(1):uhad244. https://doi.org/10.1093/hr/uhad244.

11. Bai M, Jiang S, Chu S, et al. The telomere-to-telomere (T2T) genome of *Peucedanum praeruptorum* Dunn provides insights into the genome evolution and coumarin biosynthesis. *GigaScience* 2024, 13:giae025. https://doi.org/10.1093/gigascience/giae025.

12. Zhou J, Liu Y, Guo X, et al. Centromeres: From chromosome biology to biotechnology applications and synthetic genomes in plants. *Plant Biotechnol J* 2022, 20(11):2051-2063. https://doi.org/10.1111/pbi.13875.

13. Oliveira LC, Torres GA. Plant centromeres: genetics, epigenetics and evolution. *Mol Biol Rep* 2018, 45(5):1491-1497. https://doi.org/10.1007/s11033-018-4284-7.

14. Liu Y, Yi C, Fan C, et al. Pan-centromere reveals widespread centromere repositioning of soybean genomes. *Proc Natl Acad Sci U S A* 2023, 120(42):e2310177120. https://doi.org/10.1073/pnas.2310177120.

15. Naish M, Henderson IR. The structure, function, and evolution of plant centromeres. *Genome Res* 2024, 34(2):161-178. https://doi.org/10.1101/gr.278409.123.

16. Neumann P, Navratilova A, Koblizkova A, et al. Plant centromeric retrotransposons: a structural and cytogenetic perspective. *Mob DNA* 2011, 2(1):4. https://doi.org/10.1186/1759-8753-2-4.

17. Neumann P, Oliveira L, Cizkova J, et al. Impact of parasitic lifestyle and different types of centromere organization on chromosome and genome evolution in the plant genus *Cuscuta*. *New Phytol* 2021, 229(4):2365-2377. https://doi.org/10.1111/nph.17003.

18. Sanchez-Puerta MV, Ceriotti LF, Gatica-Soria LM, et al. Invited Review Beyond parasitic convergence: unravelling the evolution of the organellar genomes in holoparasites. *Ann Bot* 2023, 132(5):909-928. https://doi.org/10.1093/aob/mcad108.

19. Harbaugh DT, Baldwin BG. Phylogeny and biogeography of the sandalwoods (*Santalum*, Santalaceae): repeated dispersals throughout the Pacific. *Am J Bot* 2007, 94 6:1028-1040. https://doi.org/10.3732/ajb.94.6.1028

20. Scartezzini P, Speroni E. Review on some plants of Indian traditional medicine with antioxidant activity. *Journal of ethnopharmacology* 2000, 71(1-2):23-43. https://doi.org/10.1016/s0378-8741(00)00213-0.

21. Kumar ANA, Joshi G, Ram HYM. Sandalwood: history, uses, present status and the future. *Curr Sci* 2012, 103(12):1408-1416.
22. Yang TQ, Zhang RA, Tian XL, et al. The chromosome-level genome assembly and genes involved in biosynthesis of nervonic acid of *Malania oleifera*. *Sci Data* 2023, 10(1):298. https://doi.org/10.1038/s41597-023-02218-8.
23. Chen X, Fang D, Xu Y, et al. *Balanophora* genomes display massively convergent evolution with other extreme holoparasites and provide novel insights into parasite–host interactions. *Nat Plants* 2023, 9(10):1627-1642. https://doi.org/10.1038/s41477-023-01517-7.
24. Fu JN, Wan LY, Song LS, et al. Chromosome-level genome assembly of the hemiparasitic *Taxillus chinensis* (DC.) Danser. *Genome Biol Evol* 2022, 14(5):evac060. https://doi.org/10.1093/gbe/evac060.
25. Lyko P, Wicke S. Genomic reconfiguration in parasitic plants involves considerable gene losses alongside global genome size inflation and gene births. *Plant Physiol* 2021, 186(3):1412-1423. https://doi.org/10.1093/plphys/kiab192.
26. Hong Z, Peng D, Tembrock LR, et al. Chromosome-level genome assemblies from two sandalwood species provide insights into the evolution of the Santalales. *Commun Biol* 2023, 6(1):587. https://doi.org/10.1038/s42003-023-04980-2.
27. Zhang XH, Li MZ, Bian Z, et al. Improved chromosome-level genome assembly of Indian sandalwood (*Santalum album*). *Sci Data* 2023, 10(1):921. https://doi.org/10.1038/s41597-023-02849-x.
28. Wang H, Liao X, Tembrock LR, et al. Evaluation of intracellular gene transfers from plastome to nuclear genome across progressively improved assemblies for *Arabidopsis thaliana* and *Oryza sativa*. *Genes-Basel* 2022, 13(9):1620. https://doi.org/10.3390/genes13091620.
29. Chen C, Wu S, Sun Y, et al. Three near-complete genome assemblies reveal substantial centromere dynamics from diploid to tetraploid in *Brachypodium* genus. *Genome Biol* 2024, 25(1):63. https://doi.org/10.1186/s13059-024-03206-w.
30. Naish M, Alonge M, Wlodzimierz P, et al. The genetic and epigenetic landscape of the *Arabidopsis* centromeres. *Science* 2021, 374(6569):eabi7489. https://doi.org/10.1126/science.abi7489.
31. Qing L. Research progress on structure and evolution of plant centromeres. *J Trop and Subtrop Bot* 2015, 23(5):576-586. https://doi.org/10.11926/j.issn.1005-3395.2015.05.013.
32. Wang G, Zhang X, Jin W. An overview of plant centromeres. *J Genet Genomics* 2009, 36(9):529-537. https://doi.org/10.1016/S1673-8527(08)60144-7.
33. Shi X, Cao S, Wang X, et al. The complete reference genome for grapevine (Vitis vinifera L.) genetics and breeding. *Hortic Res* 2023, 10(5):uhad061. https://doi.org/10.1093/hr/uhad061.
34. Chang X, He X, Li J, et al. High-quality *Gossypium hirsutum* and *Gossypium barbadense* genome assemblies reveal the landscape and evolution of centromeres. *Plant Commun* 2024, 5(2):100722. https://doi.org/10.1016/j.xplc.2023.100722.
35. Zhang H, Wang K. The research progress on plant functional centromere DNAs. *Curr Biotechnol* 2022, 12(1):1-9.
36. Lv Y, Liu C, Li X, et al. A centromere map based on super pan-genome highlights the structure and function of rice centromeres. *J Integr Plant Biol* 2024, 66(2):196-207. https://doi.org/10.1111/jipb.13607.
37. Guo N, Wang S, Gao L, et al. Genome sequencing sheds light on the contribution of structural

variants to *Brassica oleracea* diversification. *BMC Biol* 2021, 19(1):93. https://doi.org/10.1186/s12915-021-01031-2.

38. Plackova K, Bures P, Zedek F. Centromere size scales with genome size across Eukaryotes. *Sci Rep* 2021, 11(1):19811. https://doi.org/10.1038/s41598-021-99386-7.

39. Cheng H, Concepcion GT, Feng X, et al. Haplotype-resolved de novo assembly using phased assembly graphs with hifiasm. *Nat Methods* 2021, 18(2):170-175. https://doi.org/10.1038/s41592-020-01056-5.

40. Durand NC, Shamim MS, Machol I, et al. Juicer provides a one-click system for analyzing loop-resolution Hi-C experiments. *Cell Syst* 2016, 3(1):95-98. https://doi.org/10.1016/j.cels.2016.07.002.

41. Langmead B, Salzberg SL. Fast gapped-read alignment with Bowtie 2. *Nat Methods* 2012, 9(4):357-359. https://doi.org/10.1038/nmeth.1923.

42. Dudchenko O, Batra SS, Omer AD, et al. De novo assembly of the *Aedes aegypti* genome using Hi-C yields chromosome-length scaffolds. *Science* 2017, 356(6333):92-95. https://doi.org/10.1126/science.aal3327.

43. Roach MJ, Schmidt SA, Borneman AR. Purge Haplotigs: allelic contig reassignment for third-gen diploid genome assemblies. *BMC Biol* 2018, 19(1):460. https://doi.org/10.1186/s12859-018-2485-7.

44. Hu J, Wang Z, Sun Z, et al. NextDenovo: an efficient error correction and accurate assembly tool for noisy long reads. *Genome Biol* 2024, 25(1):107. https://doi.org/10.1186/s13059-024-03252-4.

45. Xu G-C, Xu T-J, Zhu R, et al. LR_Gapcloser: a tiling path-based gap closer that uses long reads to complete genome assembly. *GigaScience* 2018, 8(1):giy157. https://doi.org/10.1093/gigascience/giy157.

46. Li H. Minimap2: pairwise alignment for nucleotide sequences. *Bioinformatics* 2018, 34(18):3094-3100. https://doi.org/10.1093/bioinformatics/bty191.

47. Simao FA, Waterhouse RM, Ioannidis P, et al. BUSCO: assessing genome assembly and annotation completeness with single-copy orthologs. *Bioinformatics* 2015, 31(19):3210-3212. https://doi.org/10.1093/bioinformatics/btv351.

48. Ou S, Jiang N. LTR_retriever: A Highly Accurate and Sensitive Program for Identification of Long Terminal Repeat Retrotransposons. *Plant Physiol* 2018, 176(2):1410-1422. https://doi.org/10.1104/pp.17.01310.

49. Ou S, Su W, Liao Y, et al. Benchmarking transposable element annotation methods for creation of a streamlined, comprehensive pipeline. *Genome Biol* 2019, 20(1):275. https://doi.org/10.1186/s13059-019-1905-y.

50. Kim D, Langmead B, Salzberg SL. HISAT: a fast spliced aligner with low memory requirements. *Nat Methods* 2015, 12(4):357-360. https://doi.org/10.1038/nmeth.3317.

51. Benson G. Tandem repeats finder: a program to analyze DNA sequences. *Nucleic Acids Res* 1999, 27(2):573-580. https://doi.org/10.1093/nar/27.2.573.

52. Zhang X. Improved chromosome-level genome assembly of Indian sandalwood (*Santalum album*). figshare Dataset 2023. https://doi.org/10.6084/m9.figshare.23694729.v1.

53. Goel M, Sun H, Jiao WB, et al. SyRI: finding genomic rearrangements and local sequence differences from whole-genome assemblies. *Genome Biol* 2019, 20(1):277. https://doi.org/10.1186/s13059-019-1911-0.

54. Shumate A, Salzberg SL. Liftoff: accurate mapping of gene annotations. *Bioinformatics* 2021, 37(12):1639-1643. https://doi.org/10.1093/bioinformatics/btaa1016.

55. Quinlan AR, Hall IM. BEDTools: a flexible suite of utilities for comparing genomic features. *Bioinformatics* 2010, 26(6):841-842. https://doi.org/10.1093/bioinformatics/btq033.

56. He W, Xiang K, Chen C, et al. Master graph: an essential integrated assembly model for the plant mitogenome based on a graph-based framework. *Brief Bioinform* 2023, 24(1):bbac522. https://doi.org/10.1093/bib/bbac522.

57. Prjibelski A, Antipov D, Meleshko D, et al. Using SPAdes de novo assembler. *Curr Protoc Bioinformatics* 2020, 70(1):e102. https://doi.org/10.1002/cpbi.102.

58. Wick RR, Schultz MB, Zobel J, et al. Bandage: interactive visualization of de novo genome assemblies. *Bioinformatics* 2015, 31(20):3350-3352. https://doi.org/10.1093/bioinformatics/btv383.

59. R Core Team. R: A Language and Environment for Statistical Computing. R Foundation for Statistical Computing 2023, Vienna, Austria, https://www.R-project.org/.

60. Yin D, Chen C, Lin D, et al. Gapless genome assembly of East Asian finless porpoise. *Sci Data* 2022, 9(1):765. https://doi.org/10.1038/s41597-022-01868-4.

61. Lin Y, Ye C, Li X, et al. quarTeT: a telomere-to-telomere toolkit for gap-free genome assembly and centromeric repeat identification. *Hortic Res* 2023, 10(8):uhad127. https://doi.org/10.1093/hr/uhad127.

62. Zhang Y, Chu J, Cheng H, et al. De novo reconstruction of satellite repeat units from sequence data. *Genome Res* 2023, 33(11):1994-2001. https://doi.org/10.1101/gr.278005.123.

63. Jayakodi M, Golicz AA, Kreplak J, et al. The giant diploid faba genome unlocks variation in a global protein crop. *Nature* 2023, 615(7953):652-659. https://doi.org/10.1038/s41586-023-05791-5.

64. Cao S, Sawettalake N, Shen L. Gapless genome assembly and epigenetic profiles reveal gene regulation of whole-genome triplication in lettuce. *Gigascience* 2024, 13. https://doi.org/10.1093/gigascience/giae043.

65. Nakamura T, Yamada KD, Tomii K, et al. Parallelization of MAFFT for large-scale multiple sequence alignments. *Bioinformatics* 2018, 34(14):2490-2492. https://doi.org/10.1093/bioinformatics/bty121.

66. Criscuolo A, Gribaldo S. BMGE (Block Mapping and Gathering with Entropy): a new software for selection of phylogenetic informative regions from multiple sequence alignments. *BMC Evol Biol* 2010, 10:210. https://doi.org/10.1186/1471-2148-10-210.

67. Price MN, Dehal PS, Arkin AP. FastTree 2--approximately maximum-likelihood trees for large alignments. *PloS one* 2010, 5(3):e9490. https://doi.org/10.1371/journal.pone.0009490.

68. Letunic I, Bork P. Interactive Tree of Life (iTOL) v6: recent updates to the phylogenetic tree display and annotation tool. *Nucleic Acids Res* 2024. https://doi.org/10.1093/nar/gkae268.

69. Peng D. *S. album* assembly and annotation. figshare Dataset 2024. https://doi.org/10.6084/m9.figshare.26019481.v2.

70. Peng D; Hong Z; Kan S; Wu Z; Liao X. Supporting data for "The telomere-to-telomere (T2T) genome provides insights into the evolution of specialized centromere sequences in sandalwood" GigaScience Database 2024. https://doi.org/10.5524/102596.

Table 1. Comparision of three sandalwood assemblies

	Sal_t2t	V1 (Hong)
Total length (Mb)	218.90	229.60
Gaps	0	23
Contig N50 (Mb)	18.40	12.75
BUSCO - genome	C:98.3%[S:96.4%,D:1.9%]	C:98.2%[S:96.3%,D:1.9%]
RNA reads mapping rate - Leaf	96.12%	96.05%
NGS reads mapping rate	99.65%	98.81%
HiFi reads mapping rate	99.97%	99.98%
TE content	25.34%	28.93%
TE content - Copia	6.63%	6.10%
TE content - Gypsy	1.50%	1.09%
Gene counts	24,171	21,673
BUSCO - gene set	C:96.0%[S:93.6%,D:2.4%]	C:93.5%[S:91.3%,D:2.2%]
rRNAs	3,910	7,720
tRNAs	567	614
Transfer count - mitochondria	2,416	2,400
Transfer count - chloroplast	2,620	2,515

V2 (Zhang)
207.45
108
5.56
C:98.0%[S:95.4%,D:2.6%]
92.54%
96.93%
99.48%
22.05%
5.73%
1.81%
23,282
C:89.6%[S:86.9%,D:2.7%]
610
506
2,246
2,299

Figure 2

Click here to access/download;Figure;Figure 2.pdf

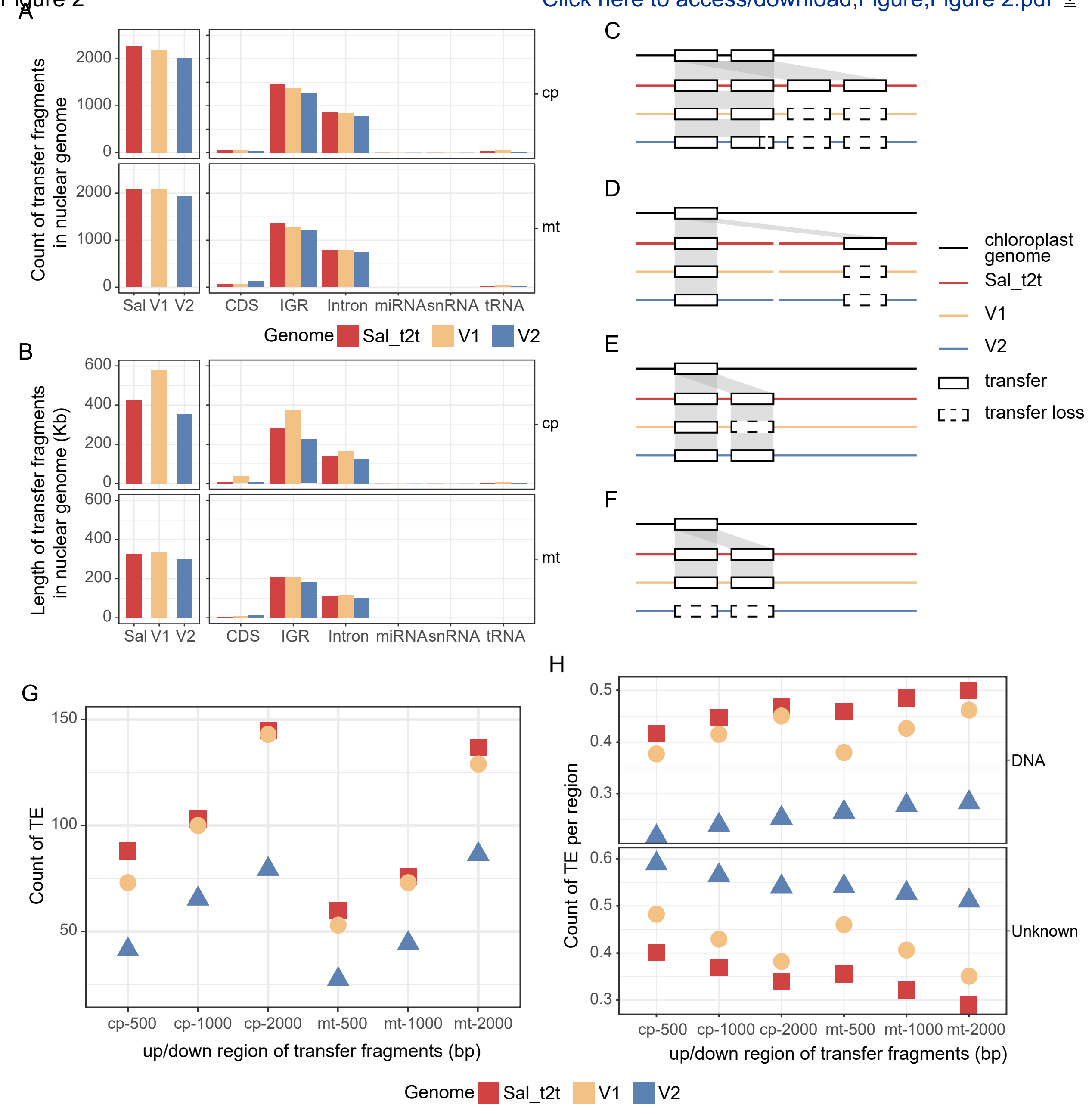

Figure 3

Click here to access/download;Figure;Figure 3.pdf

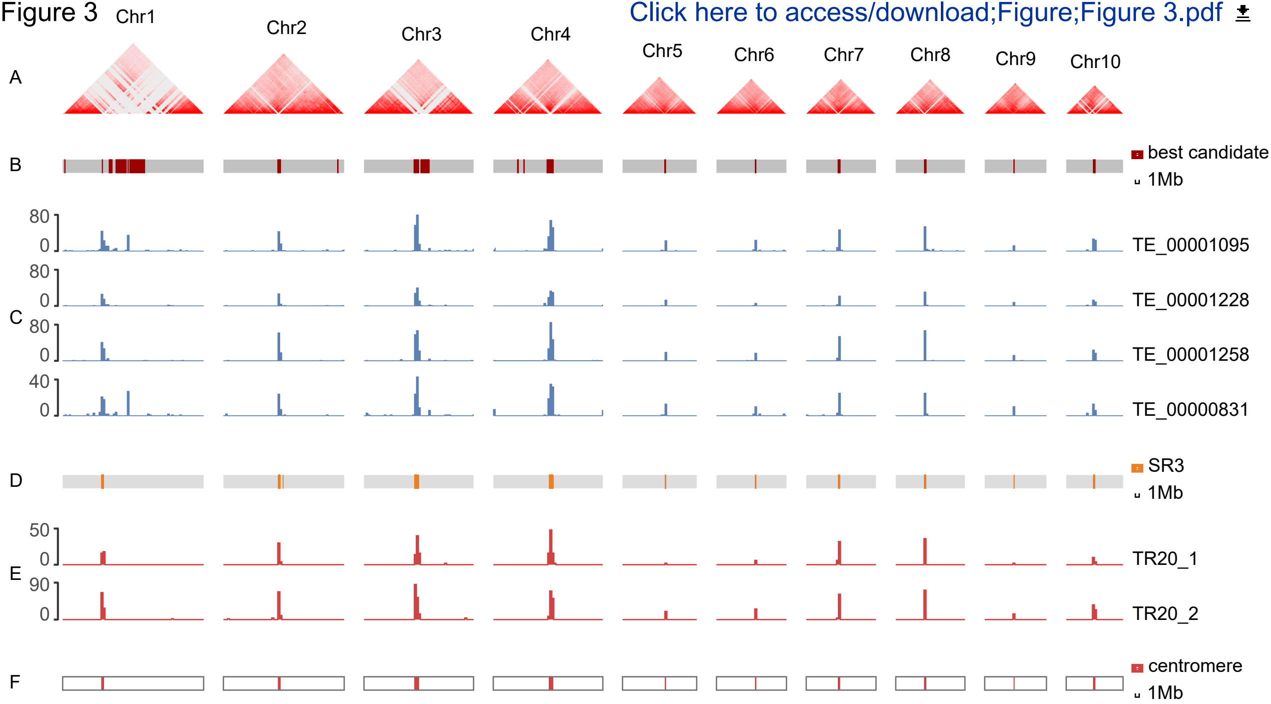

Figure 4

Click here to access/download;Figure;Figure 4.pdf

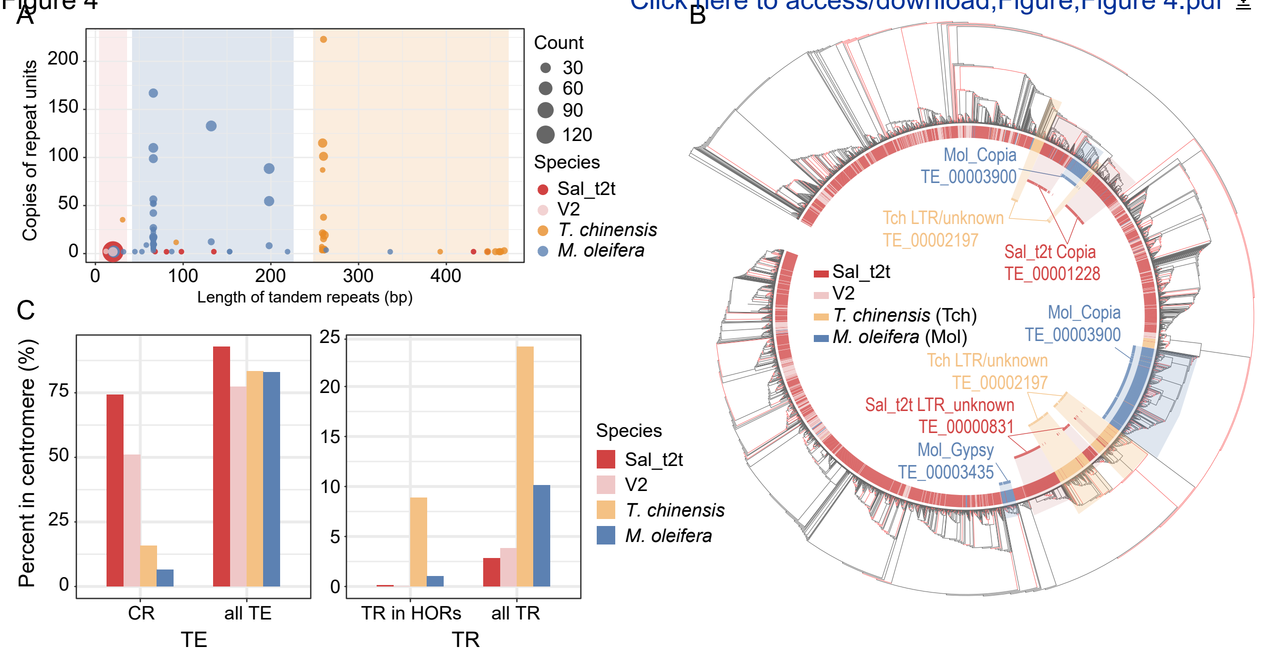

Figure 5

Click here to access/download;Figure;Figure 5.pdf

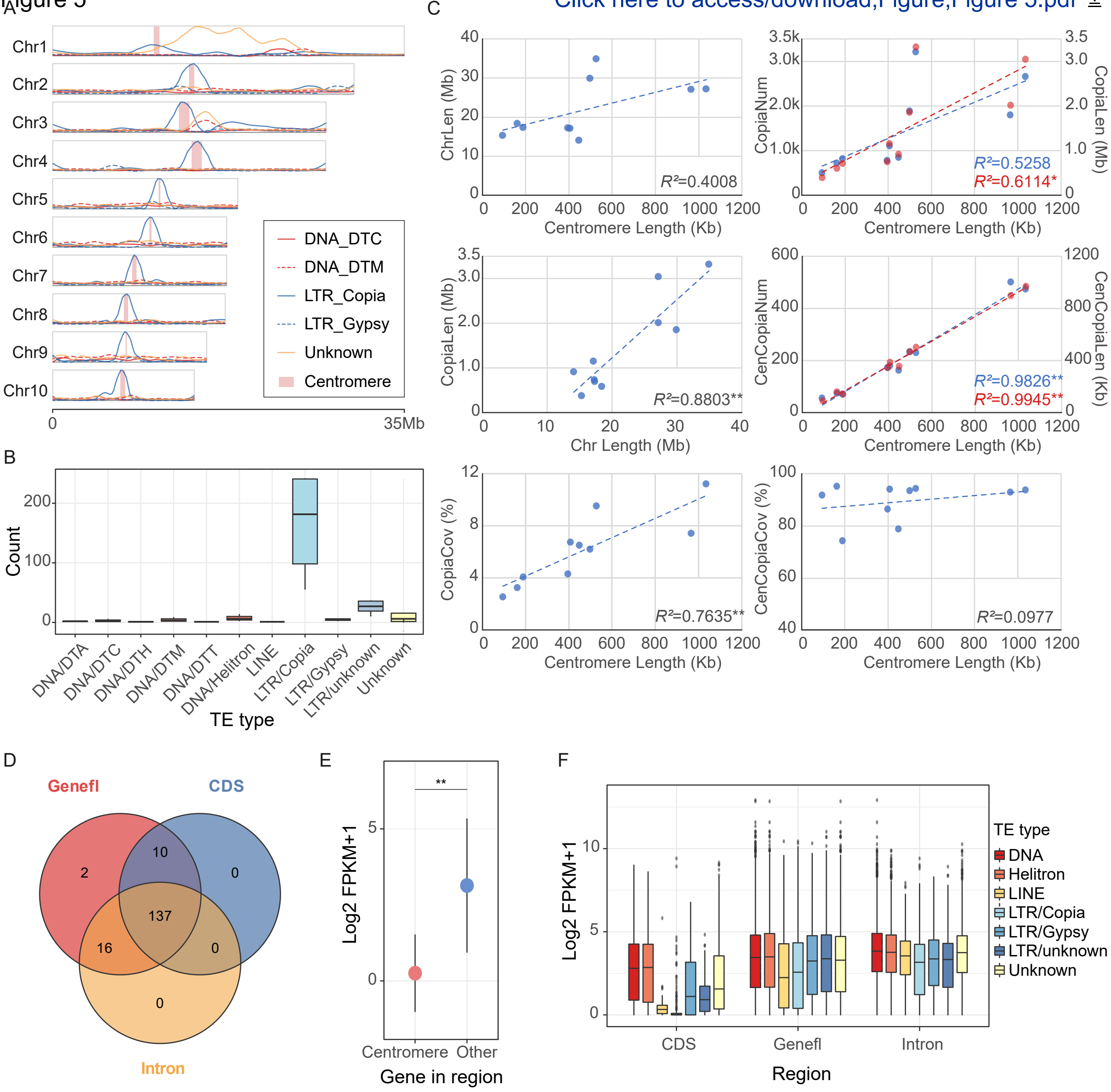

Figure 1

Click here to access/download;Figure;Figure 1.pdf

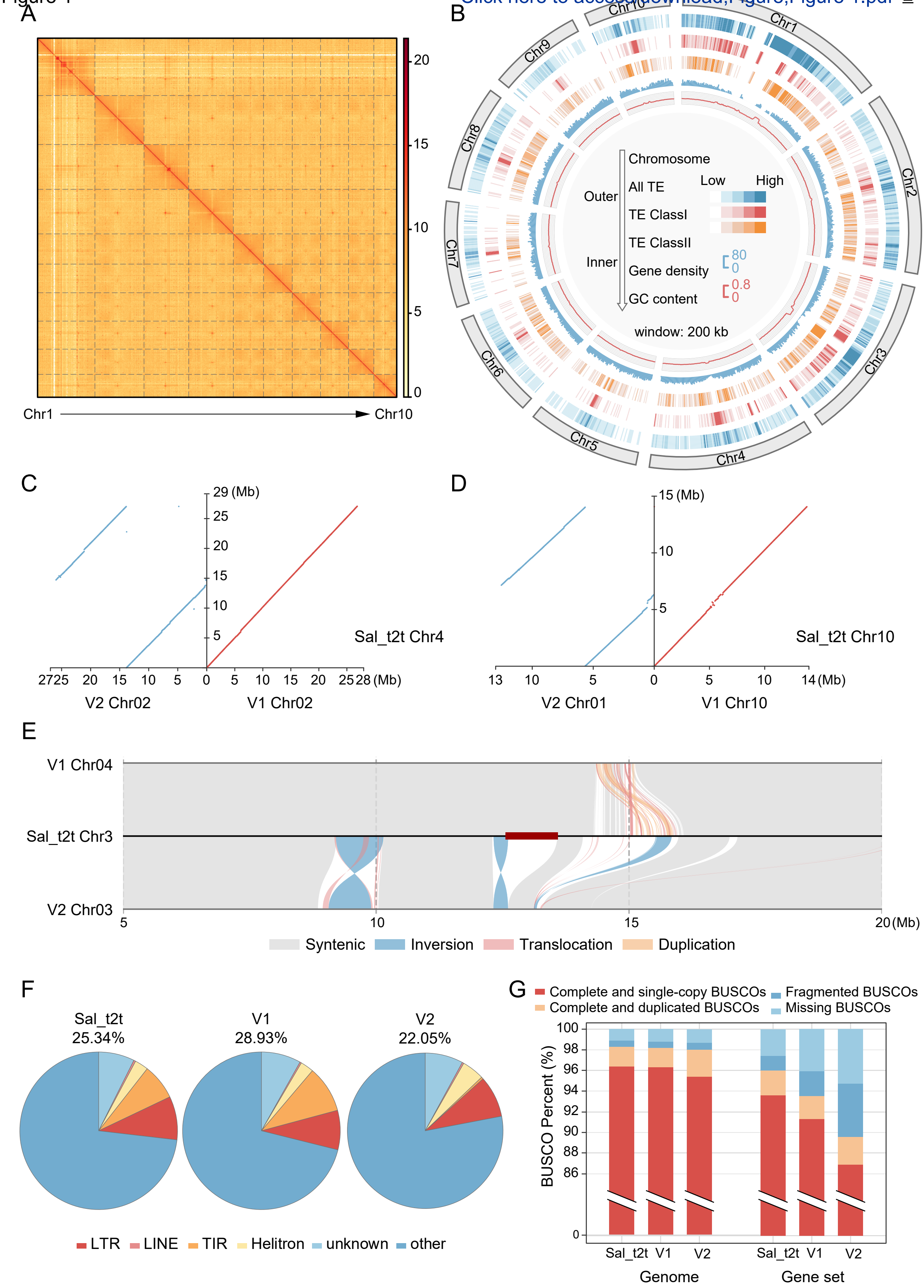

Click here to access/download
Supplementary Material
Data Resource.docx

Supplementary Fig. S1

Click here to access/download
Supplementary Material
Supplementary Fig. S1.pdf

Supplementary Fig. S2

Click here to access/download
Supplementary Material
Supplementary Fig. S2.pdf

Supplementary Fig. S3

Click here to access/download
Supplementary Material
Supplementary Fig. S3.pdf

Supplementary Fig. S4

Click here to access/download
Supplementary Material
Supplementary Fig. S4.pdf

Supplementary Fig. S5

Click here to access/download
Supplementary Material
Supplementary Fig. S5.pdf

Supplementary Fig. S6

Click here to access/download
Supplementary Material
Supplementary Fig. S6.pdf

Supplementary Fig. S7

Click here to access/download
Supplementary Material
Supplementary Fig. S7.pdf

Supplementary Fig. S8

Click here to access/download
Supplementary Material
Supplementary Fig. S8.pdf

Click here to access/download
Supplementary Material
Supplementary Fig. S9.pdf

Supplementary Fig. S10

Click here to access/download
Supplementary Material
Supplementary Fig. S10.pdf

Supplementary Fig. S11

Click here to access/download
Supplementary Material
Supplementary Fig. S11.pdf

Click here to access/download
Supplementary Material
Supplementary Table.xlsx

Response to Reviews

Dear Editor,

Thank you for considering our manuscript, “The telomere-to-telomere (T2T) genome provides insights into the evolution of specialized centromere sequences in sandalwood” (**GIGA-D-24-00225**). We sincerely appreciate the constructive comments from both reviewers. In response, we have thoroughly revised the manuscript, addressing all comments and improving the language with the help of a professional editing service. Below, we provide a detailed, point-by-point response, with reviewer comments italicized and our replies in standard font. A tracked version of the revised manuscript, with changes highlighted in red, is also included.

Thank you kindly,

Dr. Xuezhu Liao and Zhiqiang Wu

Reviewer #1

Comment 1: *Please present the results and discussion objectively to reduce the occupating coefficient of some words, such as "our" and "we".*

Response: Thank you for your suggestion. We have made the corrections in the corresponding sections of the manuscript.

Comment 2: *The language should be improved with native English speakers.*

Response: Thanks to your advice, we have revised the inaccurate statements and worked with a professional language editing service to improve the manuscript's wording.

Comment 3: *There were lots of abbreviation in the manuscript, please provide a section of abbreviation list at the end of manuscript.*

Response: We apologize for not clearly labeling the abbreviations and have added an "Abbreviations" section in line 522, page 25.

Comment 4: *Line 53,identify the exact representation of "its".*

Response: Thank you. The revised wording now reads as (line 57, page 4):

"These areas are often challenging to handle, and complete assemblies may offer a deeper understanding of the structure and function of these unassembled regions."

Comment 5: *Line 138, provide the details of paraments for LAI calculation in EDTA, which can be found in the process documents. This result is quite different with the results from LTR_retriever with the recommendation paraments from https://github.com/oushujun/LTR_retriever.*

Response: We apologize for the confusion caused by the lack of detail in the methods section. We used the default parameters of LTR_retriever to generate the "*.LAI" file, based on intermediate results from the EDTA pipeline. The command used was: LTR_retriever -genome sal.fa.mod -inharvest sal.fa.mod. EDTA.raw/LTR/sal.fa.mod.rawLTR.scn -threads 24. We have revised the relevant section of the Materials and Methods section, now reading as follows (line 403, page 20):

"The completeness of the assembled Sal_t2t genome sequences was analyzed using BUSCO (v5.3.2) (RRID:SCR_015008) [46] with the embryophyta_odb10 databases (issued 2020-08-05, including 1614 proteins), and the LAI statistic in LTR_retriever (v2.9.0) (RRID:SCR_017623) [47] with default parameters based on intermediate results from the EDTA pipeline (v1.9.4) (RRID:SCR_022063) [48]."

Comment 6: *Line 152-153, the Busco of genome with the parament of "-m geno" should be provided, which was higher than other two assembly in Table 1.*

Response: Thank you for pointing this out. We have updated the relevant section to reflect the BUSCO score using the "-m geno" parameter, which is indeed higher than the other two assemblies in Table 1. The revised text can be found in the corresponding section (line 152, page 8):

"Moreover, the completeness of the annotations was assessed using BUSCO, revealing that approximately 98.3% and 96.0% of the core genes in the assembly and gene set were complete, which was much higher than those of the V1 (98.2% and 93.5 %) and V2 (98.0% and 89.6 %) versions (Fig. 1G and Table 1)."

Comment 7: *Line 219, the region of centromeric sequences contained high density of TE and some mis-assembly might be obtained in these regions, especially for the previous assembly without T2T technology. So, which methods were adopted in centromeric region identification to reduce the mis-assembly in M. oleifera and T. chinensis.*

Response: Thank you for raising this point. To ensure reliable identification of centromeric regions in *M. oleifera* and *T. chinensis*, we used three stringent criteria: 1) inclusion of TEs enriched in certain regions while excluding scattered TEs, following the approach for CRs identification in the T2T genome; 2) identification of high-order repeat (HORs), which are key indicators of centromere presence; 3) validation through overlap between HORs and LTR-enriched regions. These regions containing both HORs (TR-enriched) and LTR-enriched were further considered as candidate centromeric regions for *M. oleifera* and *T. chinensis*. We focused on unit sequence analysis for centromeric CRs and HORs to minimize assembly errors and believe this multimetric approach provides robust results despite non-T2T assemblies.

Comment 8: *The gypsy content should be added in Table 1.*

Response: Thank you for the suggestion. We have updated Table 1 to include the *Gypsy* contents, as requested (line 547, page 27).

Comment 9: *It will be interesting by enrich the increased or disappeared genes in this T2T assembly by comparing with other two assembly.*

Response: Thank you for your suggestion. We conducted a comparative analysis of gene annotations between V1 and Sal_t2t assemblies, as V2 was excluded due to poor annotation quality. The Sal_t2t assembly revealed 164 new genes, primarily enriched in metabolic and biosynthetic processes. Of these, 29 genes were located in previously unfilled regions of V1, suggesting possible duplication. Duplication was confirmed for 152 genes, with 56 showing expression, suggesting the assembly captured biologically relevant gene duplicates. The detailed analysis is added to the "Identification of new genes" section (line 160, page 9).

"**Identification of new genes**

To test whether different assembly methods selectively enrich certain types of genes, we analysed the differences between the annotation results of the two versions, Sal_t2t (Sal_ano) and V1 (V1_ano). Using Liftoff software with Sal_ano and V1_ano as references, 21,583 and 24,007 genes were annotated to the genomes of V1 and Sal_t2t, respectively. Sal_ano contained 1,282 previously unannotated genes, while V1 contained 2,583 genes, which may be due to differences in annotation software and parameters. In addition, the de novo Sal_t2t genome assembled 164 genes, whereas V1 had 92 genes, which were identified as new genes based on Liftoff software (Supplementary Table S5).

In addition, the 164 new genes identified in Sal_t2t were mainly enriched in the GO terms oxidoreductase activity, biosynthetic processes, and metabolic processes (Supplementary Fig. S2). Whereas 29 of these 164 new

genes were located in regions located in the filled gaps of V1, and 28 of these new genes were located in the filled regions enriched with rRNAs and TRs in Sal_t2t. These genes showed high similarity and were supported by ultra-long ONT reads, suggesting that they may be duplicated. Classification of all new genes by DupGen_finder software revealed 152 (92.68%) new genes were categorized (dispersed/proximal/tandem/transposed/wgd: 26/60/36/12/18), and the proportion of duplicated genes in the genome-wide background was 70.84%. Moreover, 56 duplicate genes (dispersed/proximal/tandem/transposed/wgd: 4/18/18/5/11) were expressed (Supplementary Table S6)."

Supplementary Table S5: Liftoff homologous annotation results of Sal_t2t and V1.

	Sal_t2t	V1
Protein-coding genes	24,171	21,675
Genes predicted with Liftoff	21,583	24,007
Additional predicted genes with Liftoff	1,282	2,583
New genes (Unaligned)	164	92
Unaligned - gap	29	-
Unaligned - gap (complex)	28	-
Unaligned - other	135	-

Supplementary Table S6: Statistics of duplicated genes and the genes with expression.

	Sal_t2t		V1	
	count	exp count	count	exp count
New genes (Unaligned)	164	56	92	35
Dispersed genes	26	4	4	2
Proximal genes	60	18	36	15
Tandem genes	36	18	30	12
Transposed genes	12	5	3	2
Wgd genes	18	11	11	4

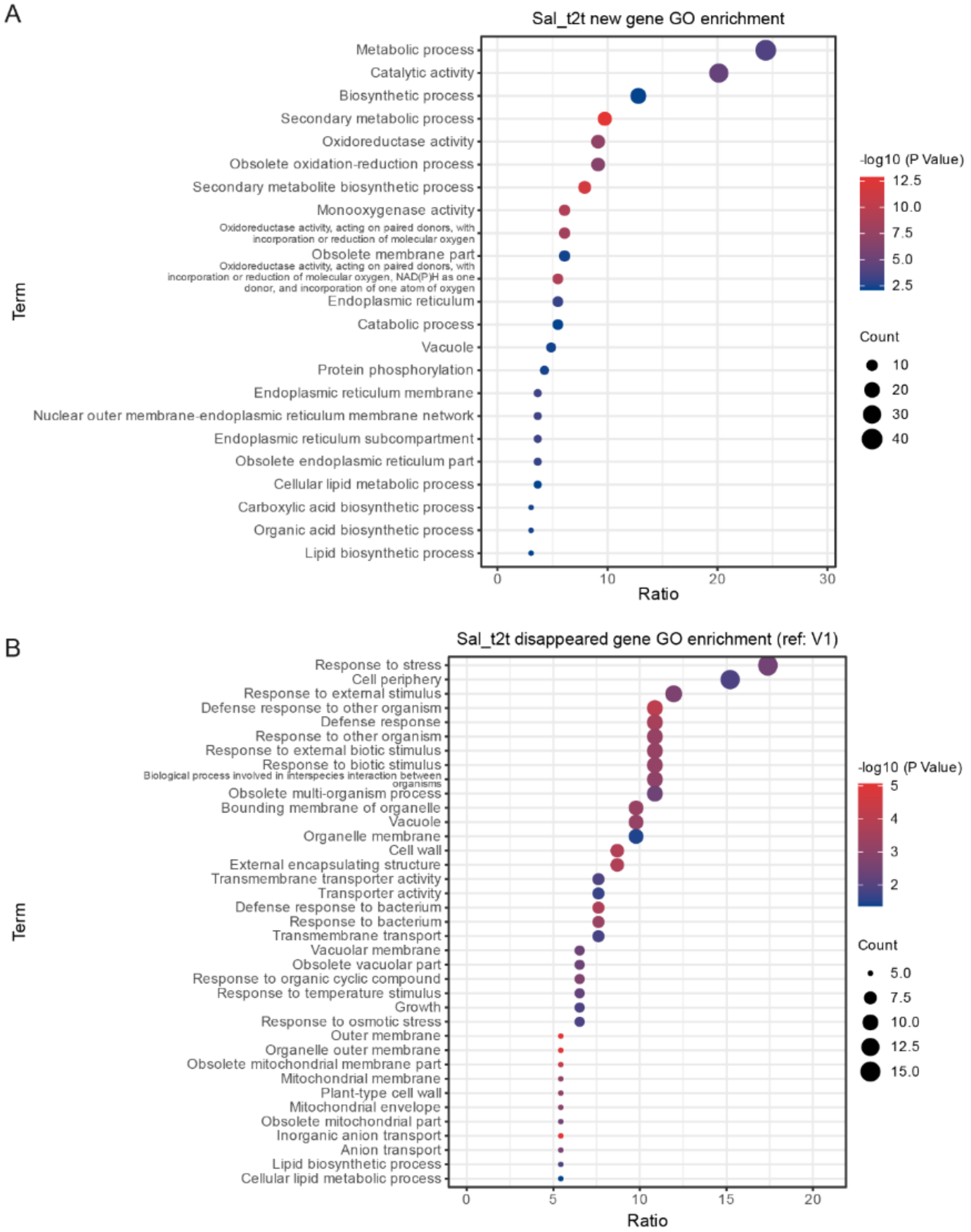

Supplementary Fig. S2: GO enrichments of new genes and disappeared genes of Sal_t2t genome.

Comment 10: *In Figure 1A, some blank was exhibited in HiC cluster, why?*

Response: Thank you for your question. Similar to what is shown in Figure 3A, these blank regions often correspond to complex areas with a high density of TEs, TRs, or regions enriched with tandem repeats of rRNAs (Supplementary Fig. S11). When mapped to such highly repetitive regions, Hi-C reads with multiple comparisons would be filtered, leading to missing signals in the Hi-C heatmap.

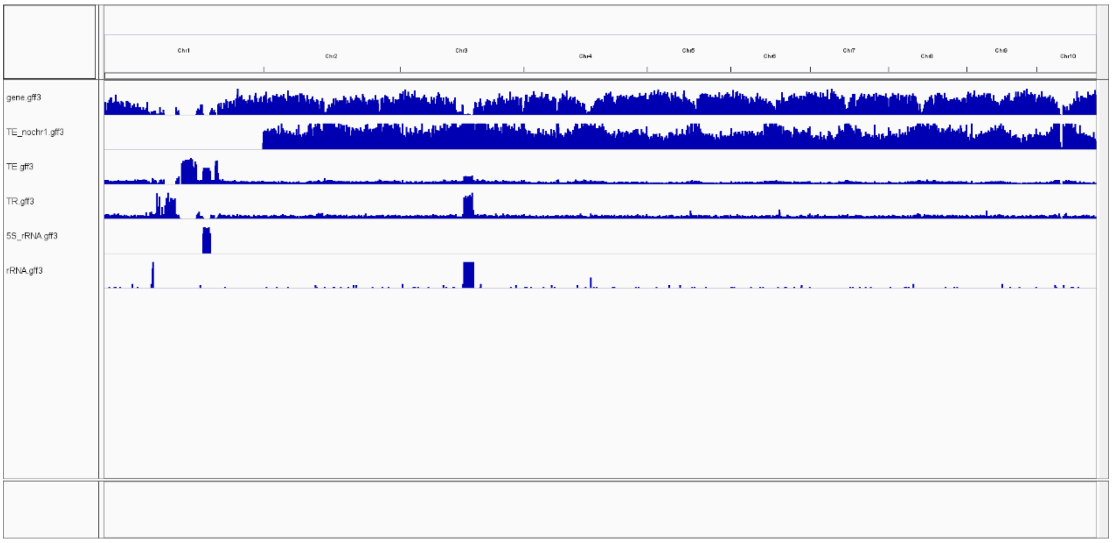

Supplementary Fig. S11: Annotations of complex regions were visualized by IGV. and the distribution of genes, TEs (excluding Chr1), all TEs, TRs, 5S rRNAs, and rRNAs other than 5S are shown from top to bottom.

***Comment 11:** In Figure 1B, why the density of TE and genes were not complementary? Total TE density was suggested to present after Chromosome in Figure 1B.*

Response: As shown in Supplementary Fig. S11 above, the second row represents the TE distribution (excluding Chr1), which is complementary to the distribution of genes. Furthermore, we have placed the total TE density after the chromosomes in Figure 1B, as suggested.

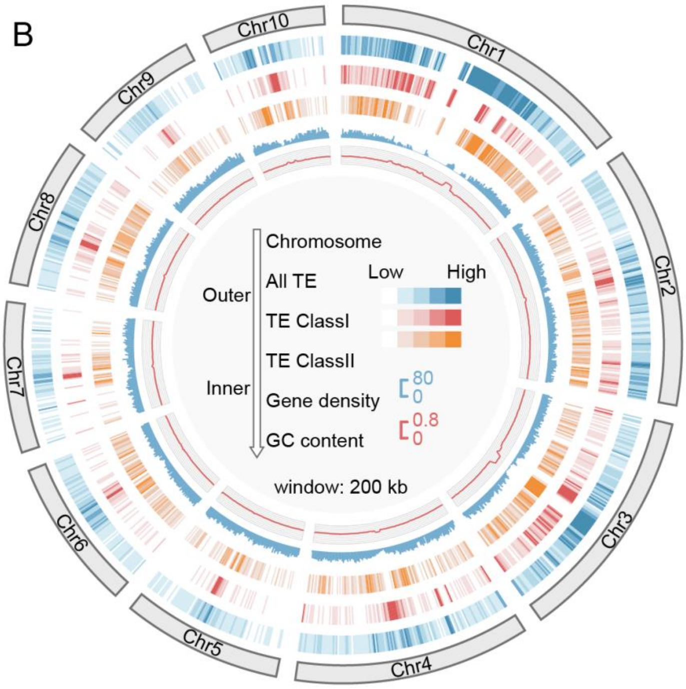

Figure 1B: Characterization of Sal_t2t genome. The density of genes, TEs and GC content were calculated per 200 Kb.

Reviewer #2

Comment 1: *This study entitled 'The telomere-to-telomere (T2T) genome provides insights into the evolution of specialized centromere sequences in sandalwood' has provided a T2T genome of sandalwood. This genome assembly is pretty well, and it contains all 20 telomeres and 10 centromeres. I think this data needs to be shown in the abstract.*

Response: Thank you for your comments. The abstract already includes the number of annotated telomeres and centromeres (lines 33, page 2). For improved clarity, we have replaced the descriptive text with Arabic numerals.

Comment 2: *On the other hand, this paper introduced a T2T genome and focused on the repeat sequences and their impacts on genome and expression. But for this sandalwood genome, I still did not find any analysis for the biology points for this species. Why the author sequenced this species? Only want to show a T2T genome?*

Response: Thank you for your valuable feedback. We understand the importance of addressing the biological significance of the sandalwood genome beyond just showcasing the T2T assembly. Our study goes beyond presenting the T2T sandalwood genome assembly; it aims to investigate the relationships between genomic features and the parasitic forms of the plant. The possibility that parasitic plant centromere length may lead to an increase in genome size has been explored in published articles, and was refuted by recent study confirming that there is no correlation between the form of parasitism and genome size [1, 2]. Building on these findings, our study focused on the centromere sequence composition and its potential relationship with parasitic forms and genome size. In the course of assembling the T2T sandalwood genome, we identified species-specific centromere sequences and explored their evolutionary dynamics in comparison to different parasitic forms. Our results demonstrate that the centromere compositions vary among the three parasitic species studied. Specifically, in sandalwood, the *Copia*-type LTR transposon predominates in the centromere, impacting gene expression and correlating positively with both centromere size and overall genome size (line 285, page 14).

Moreover, our study aligns with the goals of the T2T special issue in GigaScience, which is why we chose to submit our findings there. We hope this explanation underscores the biological insights our research contributes and addresses the concerns regarding the study's scope.

Reference:

1. Neumann P, Oliveira L, Cizkova J, et al. Impact of parasitic lifestyle and different types of centromere organization on chromosome and genome evolution in the plant genus Cuscuta. New Phytol 2021, 229(4):2365-2377. https://doi.org/10.1111/nph.17003.
2. Plackova K, Bures P, Zedek F. Centromere size scales with genome size across Eukaryotes. Sci Rep 2021, 11(1):19811. https://doi.org/10.1038/s41598-021-99386-7.

Comment 3: *For the figure5, it's better to perform this analysis among more species.*

Response: Thank you for your constructive suggestions. We have addressed your concern by including additional species with T2T genomes in our analysis. Specifically, we added lettuce, rice, and grape (Supplementary Fig. S8, line 288, page 14) to broaden the scope of our comparison. Our findings reveal that the correlations between chromosome length, number and length of centromere repeats (CRs), and centromere length in lettuce and rice are similar to those observed in sandalwood. However, we found that the coverage of CRs is negatively correlated with centromere length in these species, which contrasts with our observations in sandalwood.

Additionally, we noted that no prominent CRs were detected in grape centromeres, which made it difficult for visualization. Consequently, grape were excluded from Supplementary Fig. S8.

These results further highlight the species-specificity of sandalwood centromere composition. We have revised the results section to reflect these updates and provide a clearer understanding of the comparative analysis. The revised section now reads as follows:

"Statistics and analysis of centromere sequences in lettuce and rice showed that the correlations among chromosome length, number and length of CRs, and centromere length were similar to those in sandalwood. However, in contrast to sandalwood, the coverage of CRs was negatively correlated with centromere length, indicating that the centromere composition of sandalwood was species-specific and differed from that of the other species (Supplementary Fig. S8)."

Supplementary Table S16: Centromeric region of grape.

PN40024	Centromeric region		Shi [33]	
PN1	15,058,321	18,499,079	15,058,323	18,498,588
PN2	12,796,981	14,916,289	12,853,764	13,981,401
PN3	13,509,958	14,255,523	13,509,959	14,254,027
PN4	12,561,177	13,649,350	12,561,177	13,649,342
PN5	13,637,405	14,825,803	13,637,421	14,825,803
PN6	10,502,173	12,649,601	10,502,173	12,649,428
PN7	12,546,374	13,875,820	12,546,375	13,875,549
PN8	6,567,060	7,257,959	6,566,735	7,245,262
PN9	14,971,604	15,597,975	14,971,629	15,597,964
PN10	20,055,902	22,028,783	20,196,477	21,984,661
PN11	13,446,850	13,717,666	13,446,850	13,717,651
PN12	11,251,804	11,513,990	11,260,923	11,513,970
PN13	11,666,510	12,298,952	11,666,622	12,291,982
PN14	15,094,658	15,538,063	15,103,386	15,538,063
PN15	7,169,960	8,965,386	7,170,066	8,965,387
PN16	9,283,704	12,781,283	9,285,205	12,780,129
PN17	15,042,104	15,647,448	15,042,439	15,647,460
PN18	14,746,485	17,483,467	15,013,184	17,754,640
PN19	16,195,936	19,078,334	16,196,018	19,078,334

Supplementary Table S17: Centromeric region of rice.

NIP-T2T	Centromeric region		Shang [6]	
Chr1	16,199,590	17,692,074	16,765,400	17,553,481
Chr2	13,325,619	14,335,680	13,617,389	14,214,518
Chr3	20,033,764	20,434,133	19,954,695	20,551,000
Chr4	9,942,094	10,131,767	9,652,000	10,112,504
Chr5	12,458,668	12,621,012	12,261,000	12,840,000
Chr6	15,558,301	16,337,048	15,612,461	16,338,095
Chr7	12,074,992	12,467,395	12,108,000	12,457,000
Chr8	12,973,108	13,907,425	12,961,751	13,761,000
Chr9	6,737,398	7,282,774	6,771,892	7,241,524
Chr10	8,328,193	8,681,051	8,320,734	8,690,470
Chr11	12,546,038	14,261,944	12,803,000	14,055,729
Chr12	11,870,261	12,614,898	11,939,000	12,499,575

Supplementary Table S18: Centromeric region of lettuce.

cutv01	Centromeric region		Cao [60]	
Chr1	176,593,082	190,552,109	182,699,531	185,911,258
Chr2	88,284,582	93,866,037	88,942,479	92,160,659
Chr3	213,514,190	233,818,278	218,237,282	221,018,516
Chr4	227,085,391	277,705,034	274,382,309	277,914,143
Chr5	95,972,443	120,779,339	116,080,000	120,300,123
Chr6	106,795,078	110,629,668	107,065,176	109,801,206
Chr7	112,417,023	116,110,117	112,782,001	117,265,064
Chr8	249,258,644	264,092,051	260,739,975	263,574,706
Chr9	120,906,510	134,931,520	131,940,743	135,756,805

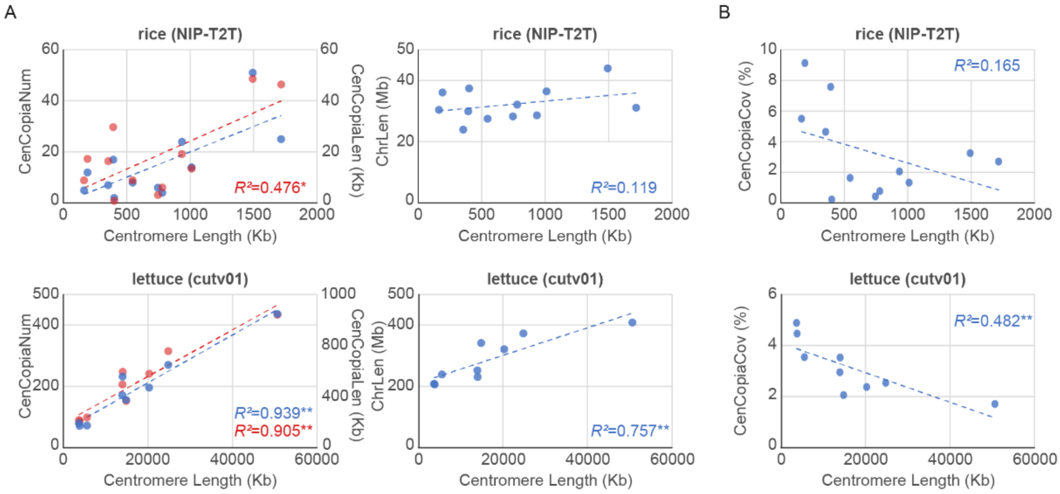

Supplementary Fig. S8: Point plot and linear correlation analysis of rice and lettuce. * represented $p < 0.05$, ** represented $p < 0.01$.

Other comments and questions

For annotation methods, the detailed parameters should be introduced.

Response: The parameters for repeats and gene annotation were added (line 417, page 20; line 419, page 20; line 425, page 21).

Line 60, The typeface of kiwifruit was different from all other words.

Response: We corrected (line 63, page 4).

Line 97, Add blank between furniture and [21].

Response: We corrected (line 99, page 6).

Line 101, sequence should be sequences.

Response: We corrected (line 103, page 6).

Line 102, assembly should be assemblies.

Response: We corrected (line 104, page 6).

Line 136, to should be onto.

Response: We corrected (line 139, page 8).

Line 166, sequence should be sequences.

Response: We corrected (line 190, page 10).

Line 309, between should be among.

Response: We corrected (line 335, page 16).

Line 320, CDS should be defined. In first time.

Response: Thank you. CDS has been defined in line 298, page 15.
