# Supplementary figures and images for: The telomere-to-telomere (T2T) genome provides insights into the evolution of specialized centromere sequences in sandalwood
Source: Gigascience. 2024 Dec 11;13:giae096. doi: 10.1093/gigascience/giae096 (PMC11633456; doi:10.1093/gigascience/giae096)

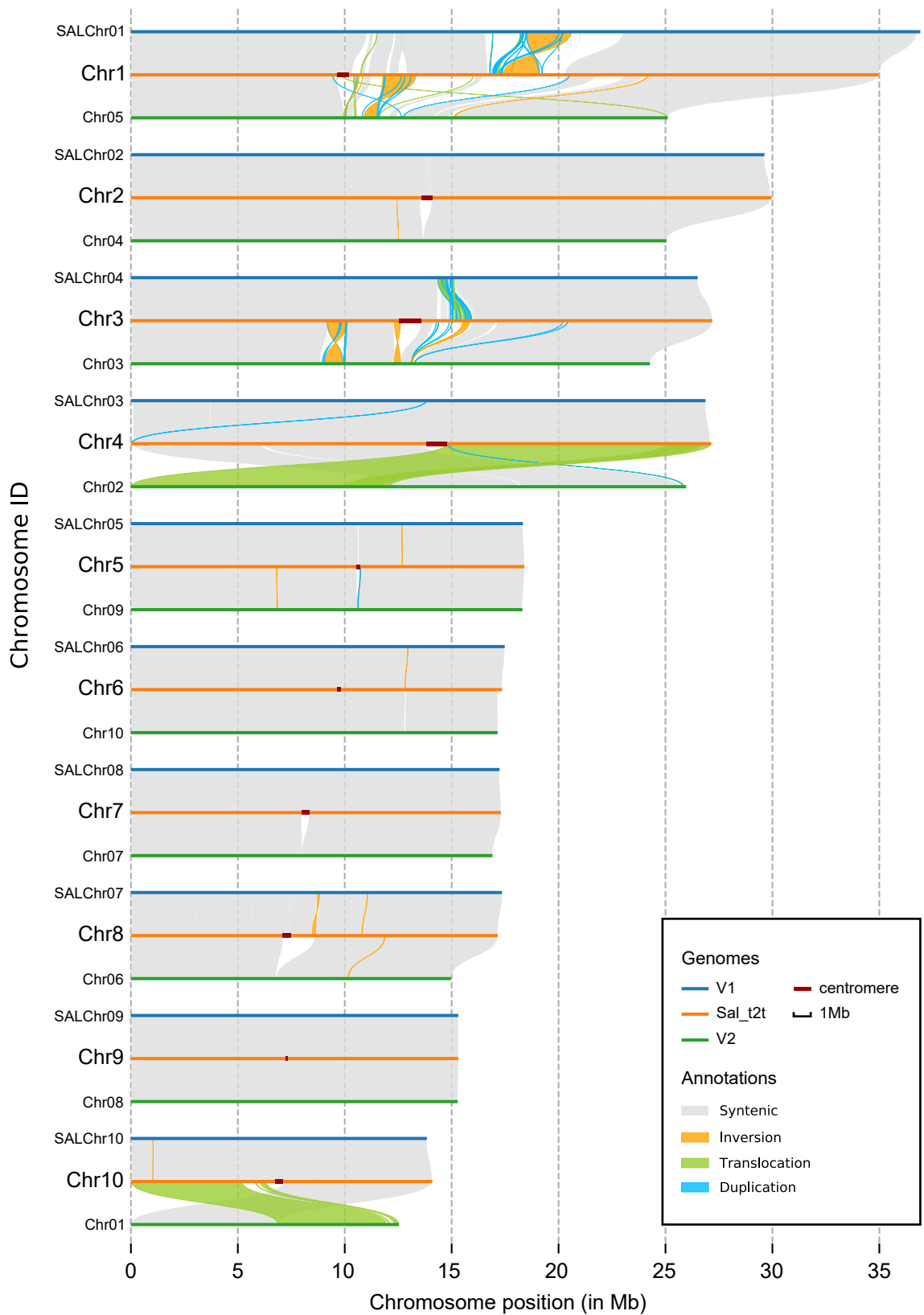

Supplement: giae096_Supplemental_Files [file giae096_supplemental_files.zip › Supplementary Fig. S1.pdf]

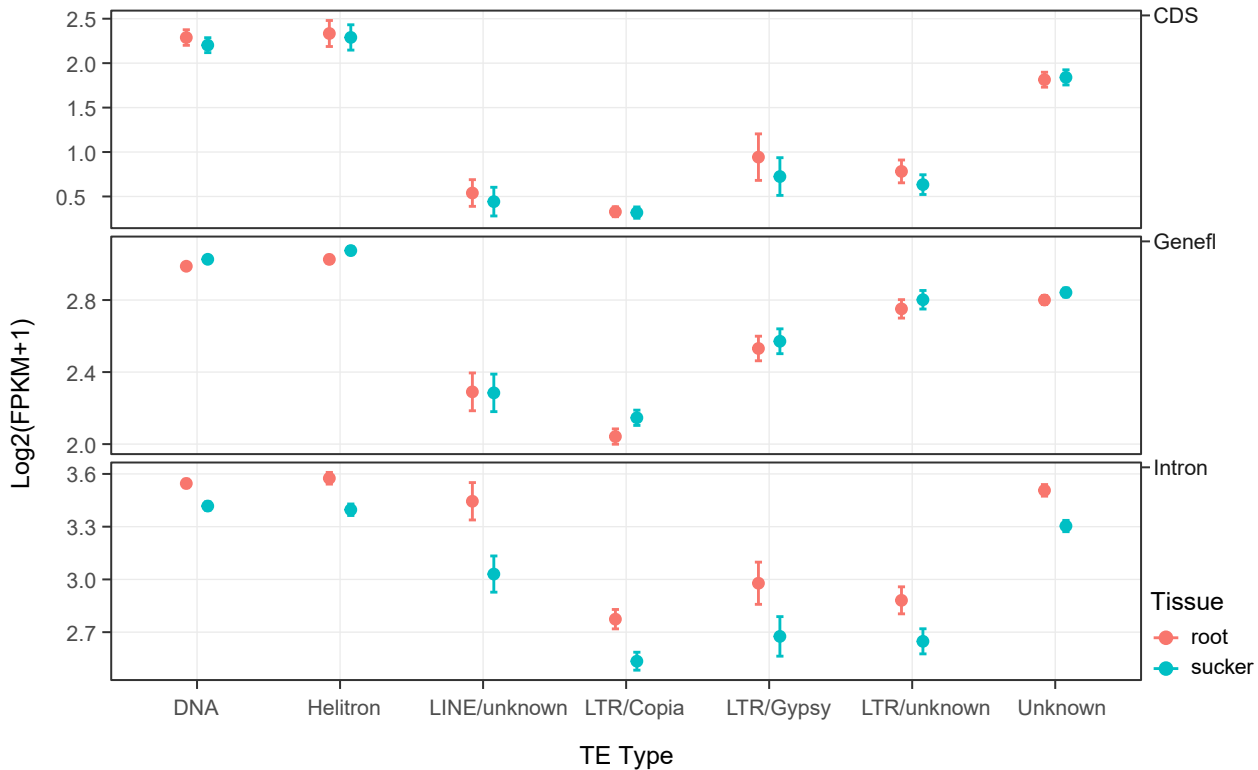

Supplement: giae096_Supplemental_Files [file giae096_supplemental_files.zip › Supplementary Fig. S10.pdf]

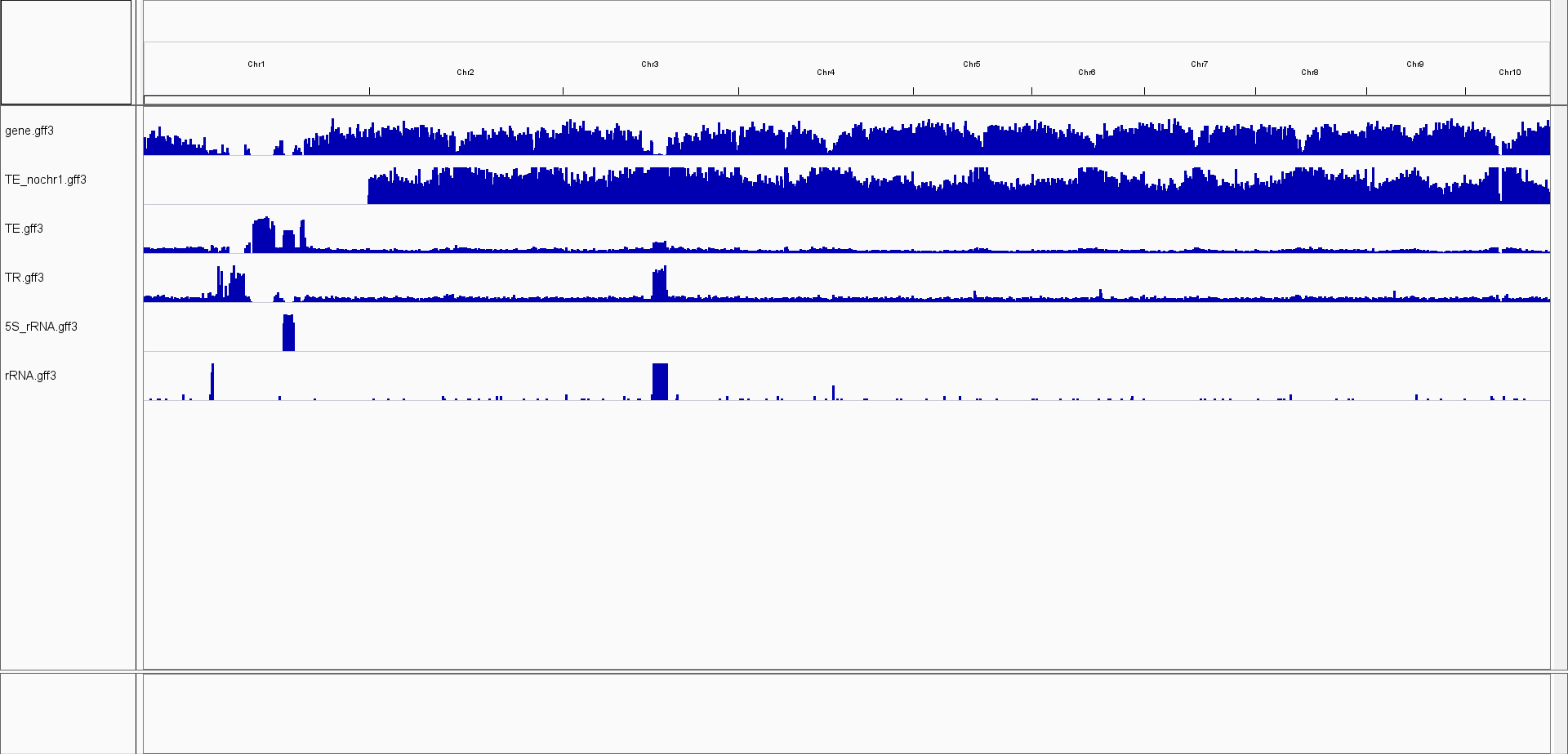

Supplement: giae096_Supplemental_Files [file giae096_supplemental_files.zip › Supplementary Fig. S11.pdf]

A

Sal\_t2t new gene GO enrichment

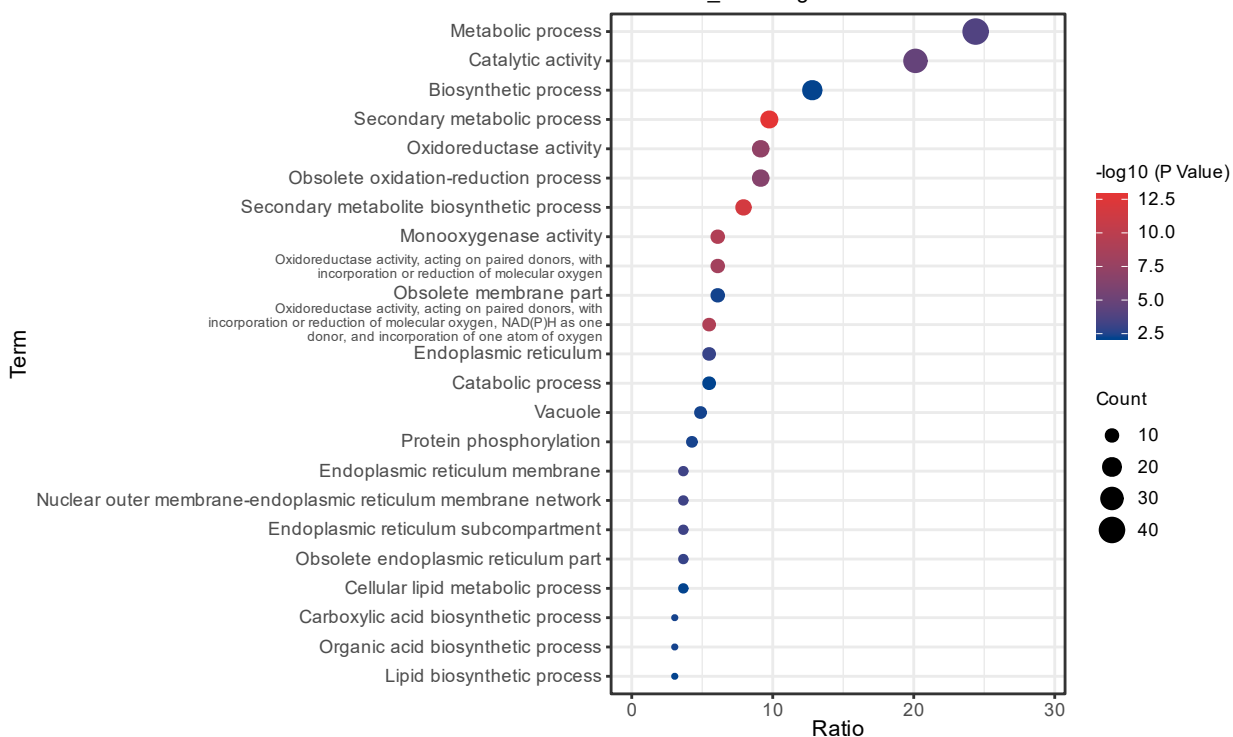

B

Sal\_t2t disappeared gene GO enrichment (ref: V1)

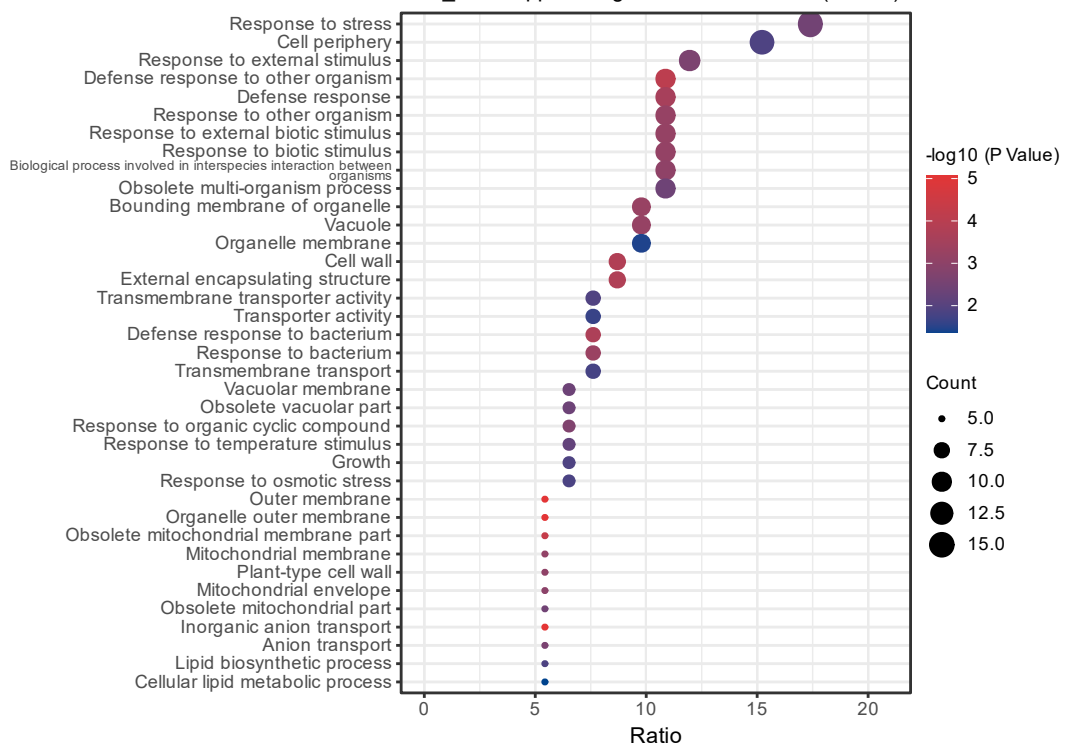

Supplement: giae096_Supplemental_Files [file giae096_supplemental_files.zip › Supplementary Fig. S2.pdf]

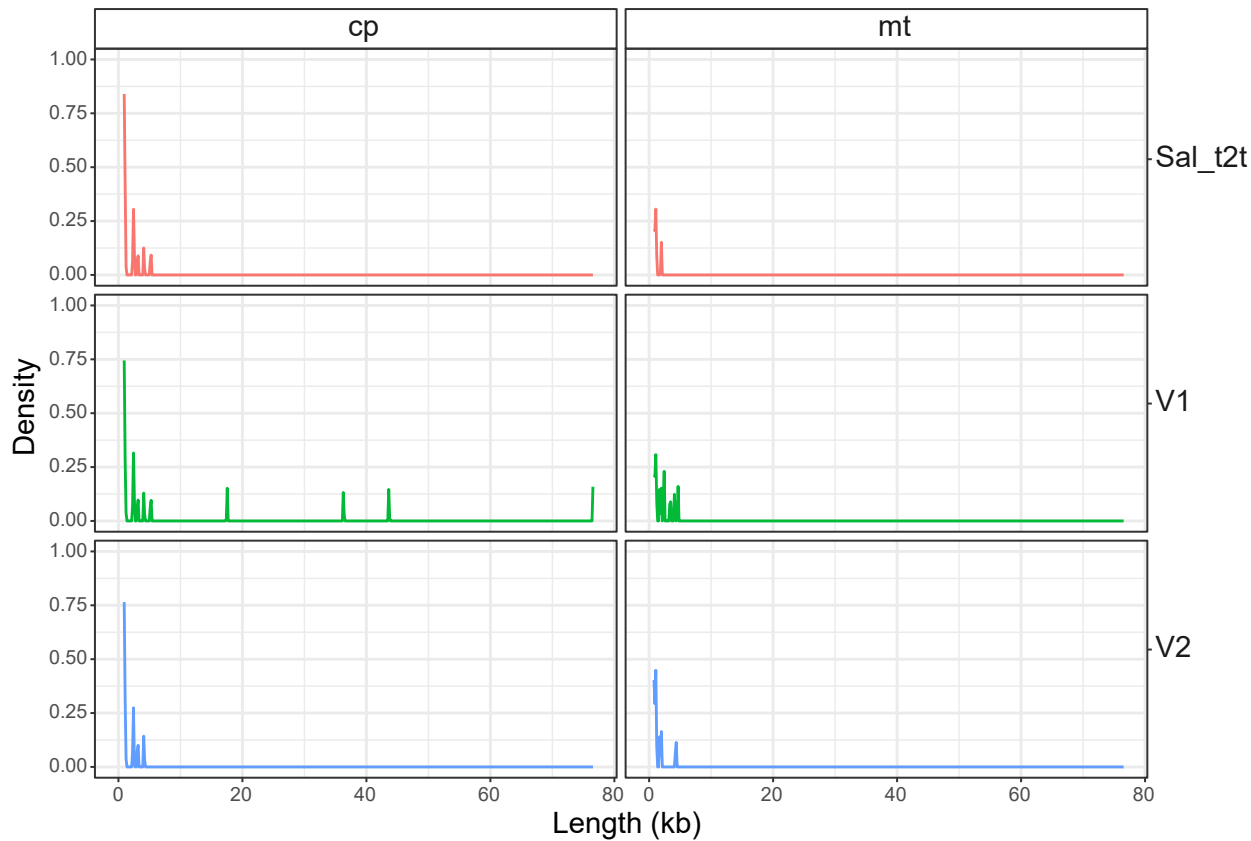

Supplement: giae096_Supplemental_Files [file giae096_supplemental_files.zip › Supplementary Fig. S3.pdf]

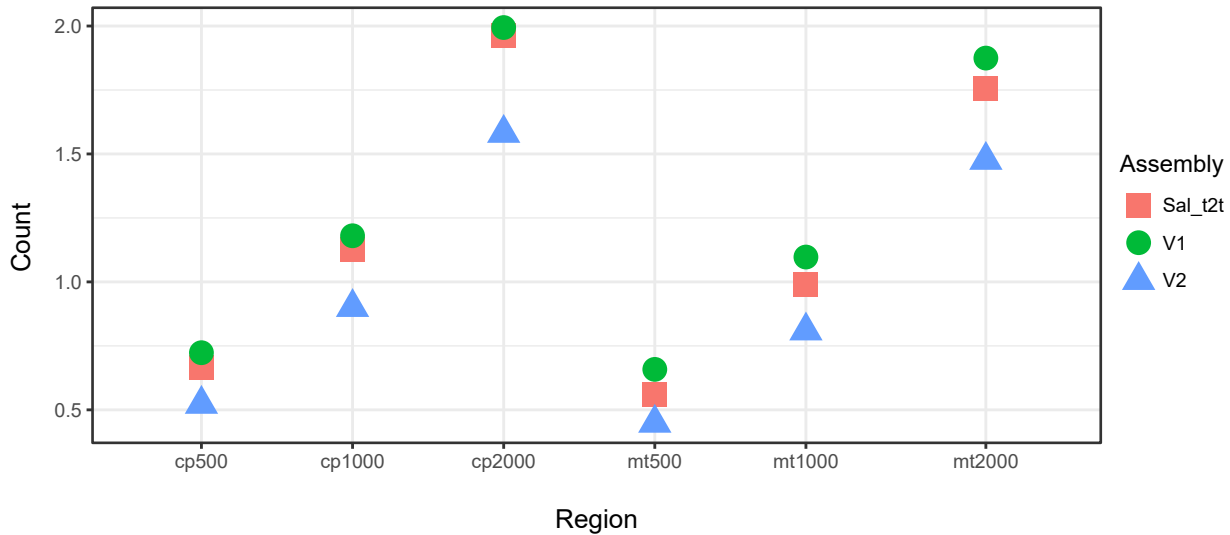

Supplement: giae096_Supplemental_Files [file giae096_supplemental_files.zip › Supplementary Fig. S4.pdf]

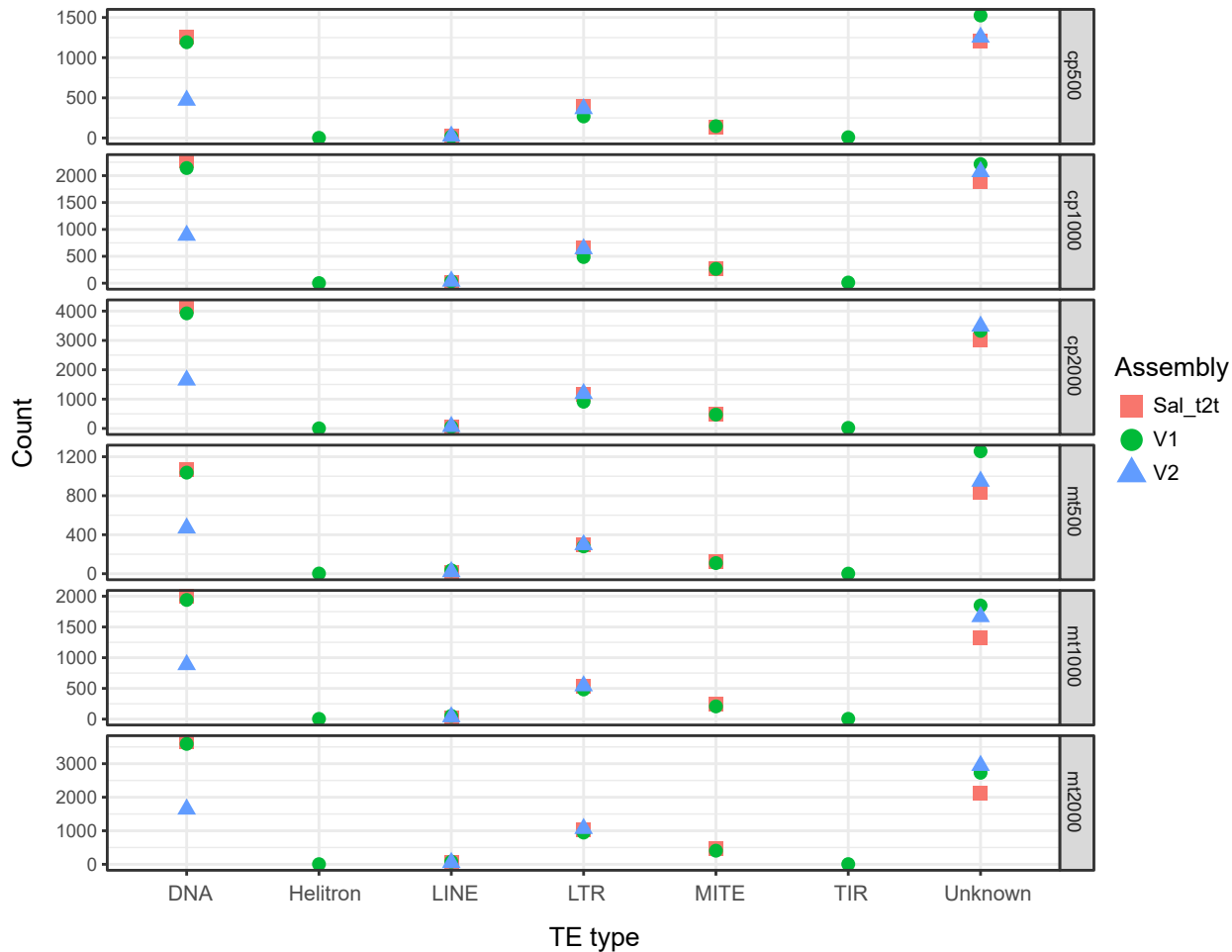

Supplement: giae096_Supplemental_Files [file giae096_supplemental_files.zip › Supplementary Fig. S5.pdf]

Count

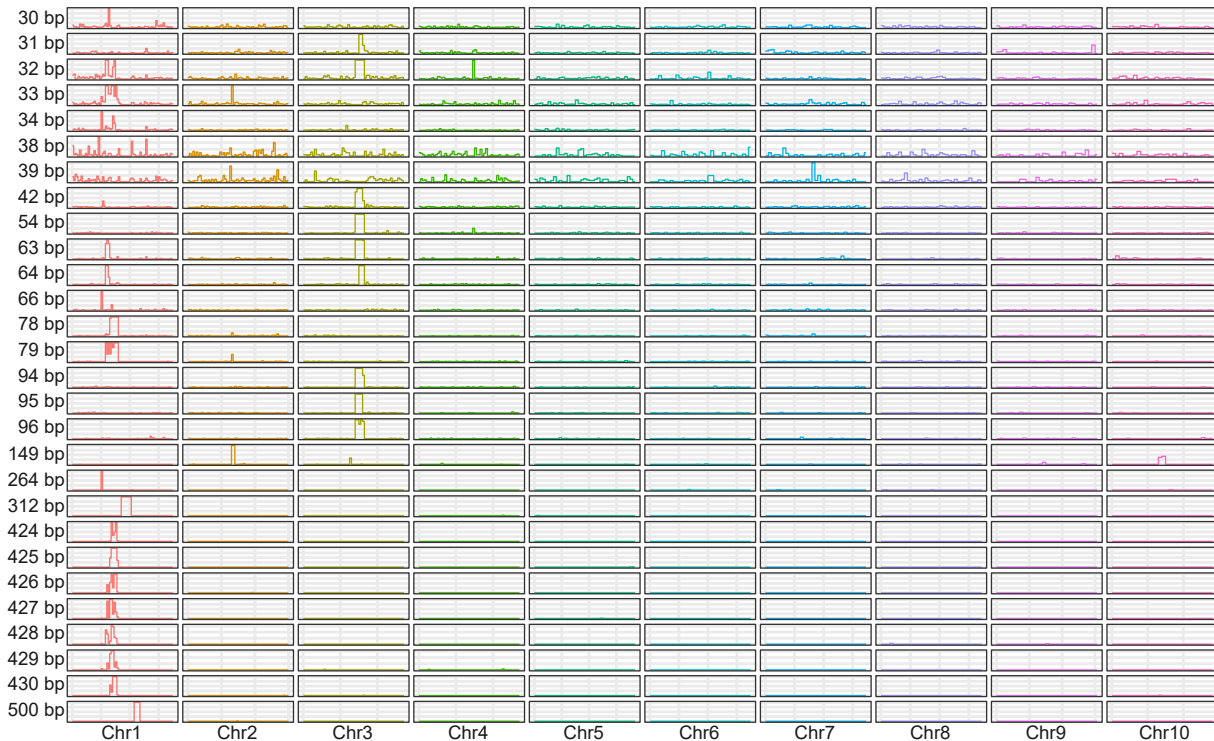

Chromosome region

5Mb

Supplement: giae096_Supplemental_Files [file giae096_supplemental_files.zip › Supplementary Fig. S6.pdf]

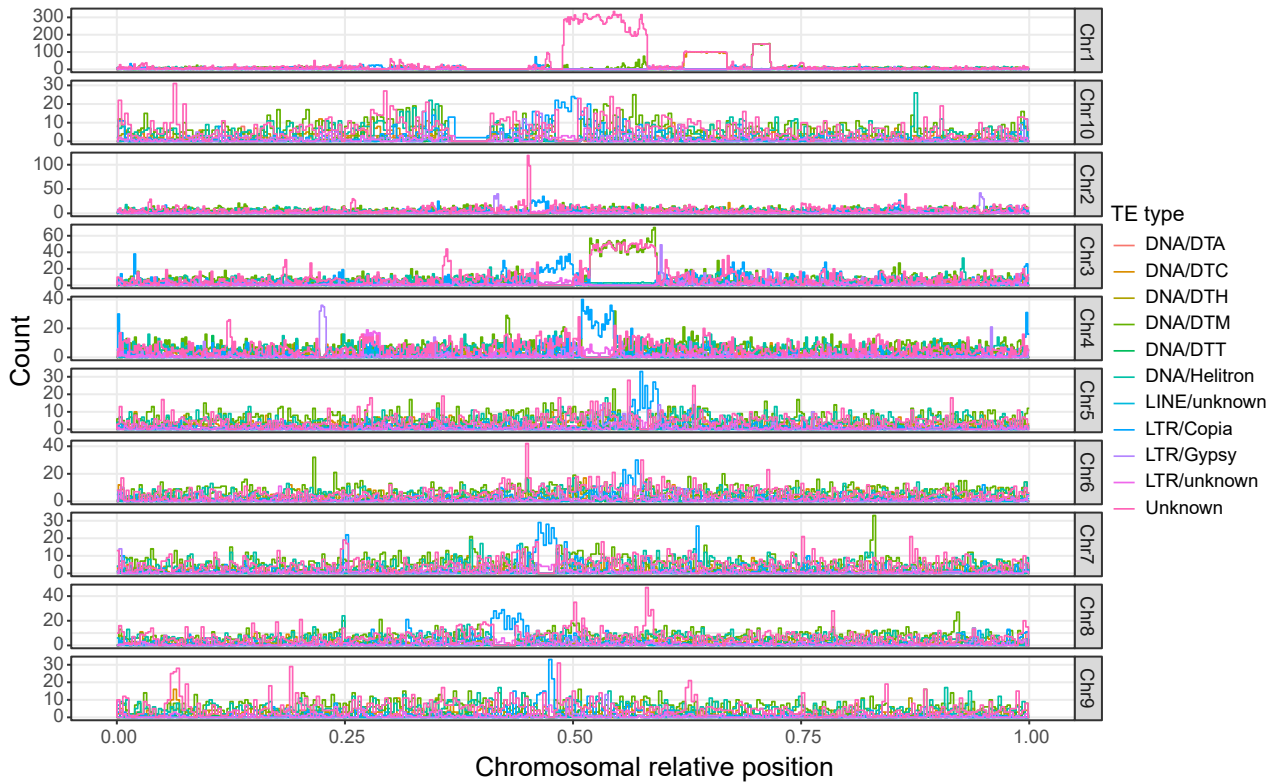

Supplement: giae096_Supplemental_Files [file giae096_supplemental_files.zip › Supplementary Fig. S7.pdf]

A

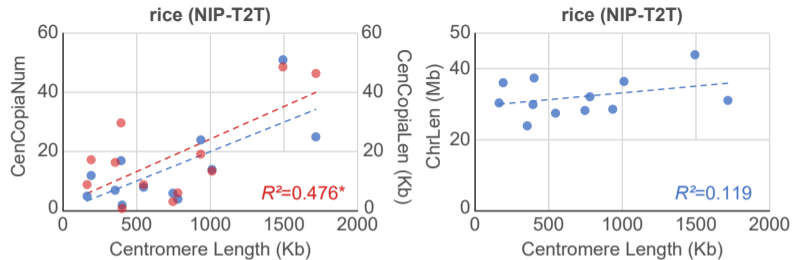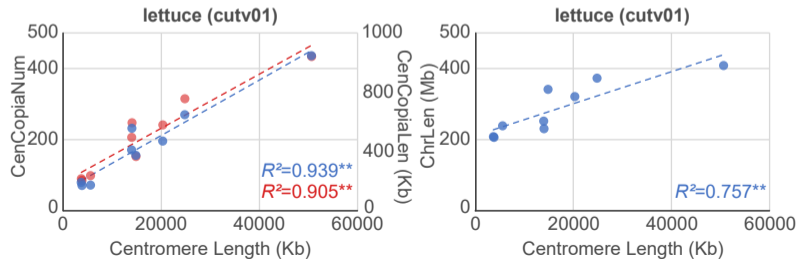

B

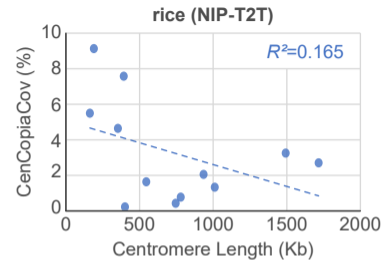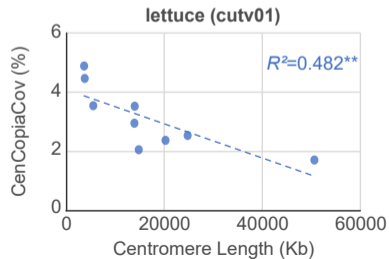

Supplement: giae096_Supplemental_Files [file giae096_supplemental_files.zip › Supplementary Fig. S8.pdf]

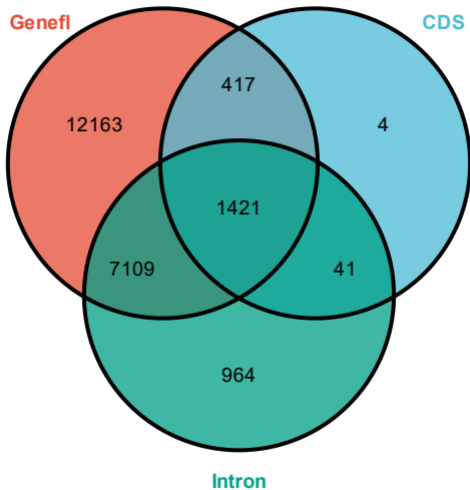

Supplement: giae096_Supplemental_Files [file giae096_supplemental_files.zip › Supplementary Fig. S9.pdf]
