# Supplementary material for: The telomere-to-telomere (T2T) genome provides insights into the evolution of specialized centromere sequences in sandalwood
Source: Gigascience. 2024 Dec 11;13:giae096. doi: 10.1093/gigascience/giae096 (PMC11633456; doi:10.1093/gigascience/giae096)
Supplement: giae096_Response_to_Reviewer_Comments_Original_Submission [file giae096_response_to_reviewer_comments_original_submission.pdf]

Author's Response To Reviewer Comments

Reviewer #1
Comment 1: Please present the results and discussion objectively to reduce the occupating coefficient of some words, such as "our" and "we".
Response: Thank you for your suggestion. We have made the corrections in the corresponding sections of the manuscript.

Comment 2: The language should be improved with native English speakers.
Response: Thanks to your advice, we have revised the inaccurate statements and worked with a professional language editing service to improve the manuscript's wording.

Comment 3: There were lots of abbreviation in the manuscript, please provide a section of abbreviation list at the end of manuscript.
Response: We apologize for not clearly labeling the abbreviations and have added an "Abbreviations" section in line 522, page 25.

Comment 4: Line 53,identify the exact representation of "its".
Response: Thank you. The revised wording now reads as (line 57, page 4):
"These areas are often challenging to handle, and complete assemblies may offer a deeper understanding of the structure and function of these unassembled regions."

Comment 5: Line 138, provide the details of paraments for LAI calculation in EDTA, which can be found in the process documents. This result is quite different with the results from LTR_retriever with the recommendation paraments from https://github.com/oushujun/LTR_retriever.
Response: We apologize for the confusion caused by the lack of detail in the methods section. We used the default parameters of LTR_retriever to generate the "*.LAI" file, based on intermediate results from the EDTA pipeline. The command used was: LTR_retriever -genome sal.fa.mod -inharvest sal.fa.mod.
EDTA.raw/LTR/sal.fa.mod.rawLTR.scn -threads 24. We have revised the relevant section of the Materials and Methods section, now reading as follows (line 403, page 20):
"The completeness of the assembled Sal_t2t genome sequences was analyzed using BUSCO (v5.3.2) (RRID:SCR_015008) [46] with the embryophyta_odb10 databases (issued 2020-08-05, including 1614 proteins), and the LAI statistic in LTR_retriever (v2.9.0) (RRID:SCR_017623) [47] with default parameters based on intermediate results from the EDTA pipeline (v1.9.4) (RRID:SCR_022063) [48]."

Comment 6: Line 152-153, the Busco of genome with the parament of "-m geno" should be provided, which was higher than other two assembly in Table 1.
Response: Thank you for pointing this out. We have updated the relevant section to reflect the BUSCO score using the "-m geno" parameter, which is indeed higher than the other two assemblies in Table 1. The revised text can be found in the corresponding section (line 152, page 8):
"Moreover, the completeness of the annotations was assessed using BUSCO, revealing that approximately 98.3% and 96.0% of the core genes in the assembly and gene set were complete, which was much higher than those of the V1 (98.2% and 93.5 %) and V2 (98.0% and 89.6 %) versions (Fig. 1G and Table 1)."

Comment 7: Line 219, the region of centromeric sequences contained high density of TE and some mis-assembly might be obtained in these regions, especially for the previous assembly without T2T technology. So, which methods were adopted in centromeric region identification to reduce the mis-assembly in M. oleifera and T. chinensis.
Response: Thank you for raising this point. To ensure reliable identification of centromeric regions in M. oleifera and T. chinensis, we used three stringent criteria: 1) inclusion of TEs enriched in certain regions while excluding scattered TEs, following the approach for CRs identification in the T2T genome; 2) identification of high-order repeat (HORs), which are key indicators of centromere presence; 3) validation through overlap between HORs and LTR-enriched regions. These regions containing both HORs (TR-enriched) and LTR-enriched were further considered as candidate centromeric regions for M. oleifera and T. chinensis. We focused on unit sequence analysis for centromeric CRs and HORs to minimize assembly

errors and believe this multimetric approach provides robust results despite non-T2T assemblies.

Comment 8: The gypsy content should be added in Table 1.
Response: Thank you for the suggestion. We have updated Table 1 to include the Gypsy contents, as requested (line 547, page 27).

Comment 9: It will be interesting by enrich the increased or disappeared genes in this T2T assembly by comparing with other two assembly.
Response: Thank you for your suggestion. We conducted a comparative analysis of gene annotations between V1 and Sal_t2t assemblies, as V2 was excluded due to poor annotation quality. The Sal_t2t assembly revealed 164 new genes, primarily enriched in metabolic and biosynthetic processes. Of these, 29 genes were located in previously unfilled regions of V1, suggesting possible duplication. Duplication was confirmed for 152 genes, with 56 showing expression, suggesting the assembly captured biologically relevant gene duplicates. The detailed analysis is added to the "Identification of new genes" section (line 160, page 9).
"Identification of new genes
To test whether different assembly methods selectively enrich certain types of genes, we analysed the differences between the annotation results of the two versions, Sal_t2t (Sal_ano) and V1 (V1_ano). Using Liftoff software with Sal_ano and V1_ano as references, 21,583 and 24,007 genes were annotated to the genomes of V1 and Sal_t2t, respectively. Sal_ano contained 1,282 previously unannotated genes, while V1 contained 2,583 genes, which may be due to differences in annotation software and parameters. In addition, the de novo Sal_t2t genome assembled 164 genes, whereas V1 had 92 genes, which were identified as new genes based on Liftoff software (Supplementary Table S5).
In addition, the 164 new genes identified in Sal_t2t were mainly enriched in the GO terms oxidoreductase activity, biosynthetic processes, and metabolic processes (Supplementary Fig. S2). Whereas 29 of these 164 new genes were located in regions located in the filled gaps of V1, and 28 of these new genes were located in the filled regions enriched with rRNAs and TRs in Sal_t2t. These genes showed high similarity and were supported by ultra-long ONT reads, suggesting that they may be duplicated. Classification of all new genes by DupGen_finder software revealed 152 (92.68%) new genes were categorized (dispersed/proximal/tandem/transposed/wgd: 26/60/36/12/18), and the proportion of duplicated genes in the genome-wide background was 70.84%. Moreover, 56 duplicate genes (dispersed/proximal/tandem/transposed/wgd: 4/18/18/5/11) were expressed (Supplementary Table S6)."

Comment 10: In Figure 1A, some blank was exhibited in HiC cluster, why?
Response: Thank you for your question. Similar to what is shown in Figure 3A, these blank regions often correspond to complex areas with a high density of TEs, TRs, or regions enriched with tandem repeats of rRNAs (Supplementary Fig. S11). When mapped to such highly repetitive regions, Hi-C reads with multiple comparisons would be filtered, leading to missing signals in the Hi-C heatmap.

Comment 11: In Figure 1B, why the density of TE and genes were not complementary? Total TE density was suggested to present after Chromosome in Figure 1B.
Response: As shown in Supplementary Fig. S11 above, the second row represents the TE distribution (excluding Chr1), which is complementary to the distribution of genes. Furthermore, we have placed the total TE density after the chromosomes in Figure 1B, as suggested.

Reviewer #2
Comment 1: This study entitled 'The telomere-to-telomere (T2T) genome provides insights into the evolution of specialized centromere sequences in sandalwood' has provided a T2T genome of sandalwood. This genome assembly is pretty well, and it contains all 20 telomeres and 10 centromeres. I think this data needs to be shown in the abstract.
Response: Thank you for your comments. The abstract already includes the number of annotated telomeres and centromeres (lines 33, page 2). For improved clarity, we have replaced the descriptive text with Arabic numerals.

Comment 2: On the other hand, this paper introduced a T2T genome and focused on the repeat sequences and their impacts on genome and expression. But for this sandalwood genome, I still did not find any analysis for the biology points for this species. Why the author sequenced this species? Only want to show

a T2T genome?
Response: Thank you for your valuable feedback. We understand the importance of addressing the biological significance of the sandalwood genome beyond just showcasing the T2T assembly. Our study goes beyond presenting the T2T sandalwood genome assembly; it aims to investigate the relationships between genomic features and the parasitic forms of the plant. The possibility that parasitic plant centromere length may lead to an increase in genome size has been explored in published articles, and was refuted by recent study confirming that there is no correlation between the form of parasitism and genome size [1, 2]. Building on these findings, our study focused on the centromere sequence composition and its potential relationship with parasitic forms and genome size. In the course of assembling the T2T sandalwood genome, we identified species-specific centromere sequences and explored their evolutionary dynamics in comparison to different parasitic forms. Our results demonstrate that the centromere compositions vary among the three parasitic species studied. Specifically, in sandalwood, the Copia-type LTR transposon predominates in the centromere, impacting gene expression and correlating positively with both centromere size and overall genome size (line 285, page 14).
Moreover, our study aligns with the goals of the T2T special issue in GigaScience, which is why we chose to submit our findings there. We hope this explanation underscores the biological insights our research contributes and addresses the concerns regarding the study's scope.
Reference：
1. Neumann P, Oliveira L, Cizkova J, et al. Impact of parasitic lifestyle and different types of centromere organization on chromosome and genome evolution in the plant genus Cuscuta. New Phytol 2021, 229(4):2365-2377. https://doi.org/10.1111/nph.17003.
2. Plackova K, Bures P, Zedek F. Centromere size scales with genome size across Eukaryotes. Sci Rep 2021, 11(1):19811. https://doi.org/10.1038/s41598-021-99386-7.

Comment 3: For the figure5, it's better to perform this analysis among more species.
Response: Thank you for your constructive suggestions. We have addressed your concern by including additional species with T2T genomes in our analysis. Specifically, we added lettuce, rice, and grape (Supplementary Fig. S8, Supplementary Tables S16-18, line 288, page 14) to broaden the scope of our comparison. Our findings reveal that the correlations between chromosome length, number and length of centromere repeats (CRs), and centromere length in lettuce and rice are similar to those observed in sandalwood. However, we found that the coverage of CRs is negatively correlated with centromere length in these species, which contrasts with our observations in sandalwood.
Additionally, we noted that no prominent CRs were detected in grape centromeres, which made it difficult for visualization. Consequently, grape were excluded from Supplementary Fig. S8.
These results further highlight the species-specificity of sandalwood centromere composition. We have revised the results section to reflect these updates and provide a clearer understanding of the comparative analysis. The revised section now reads as follows:
"Statistics and analysis of centromere sequences in lettuce and rice showed that the correlations among chromosome length, number and length of CRs, and centromere length were similar to those in sandalwood. However, in contrast to sandalwood, the coverage of CRs was negatively correlated with centromere length, indicating that the centromere composition of sandalwood was species-specific and differed from that of the other species (Supplementary Fig. S8)."

Other comments and questions
For annotation methods, the detailed parameters should be introduced.
Response: The parameters for repeats and gene annotation were added (line 417, page 20; line 419, page 20; line 425, page 21).

Line 60, The typeface of kiwifruit was different from all other words.
Response: We corrected (line 63, page 4).

Line 97, Add blank between furniture and [21].
Response: We corrected (line 99, page 6).

Line 101, sequence should be sequences.
Response: We corrected (line 103, page 6).

Line 102, assembly should be assemblies.
Response: We corrected (line 104, page 6).

Line 136, to should be onto.
Response: We corrected (line 139, page 8).

Line 166, sequence should be sequences.
Response: We corrected (line 190, page 10).

Line 309, between should be among.
Response: We corrected (line 335, page 16).

Line 320, CDS should be defined. In first time.
Response: Thank you. CDS has been defined in line 298, page 15.
