# Supplementary material for: The telomere-to-telomere (T2T) genome provides insights into the evolution of specialized centromere sequences in sandalwood
Source: Gigascience. 2024 Dec 11;13:giae096. doi: 10.1093/gigascience/giae096 (PMC11633456; doi:10.1093/gigascience/giae096)
Supplement: giae096_Reviewer_2_Report_Original_Submission — Chao Bian, ph.D -- 7/24/2024 Reviewed [file giae096_reviewer_2_report_original_submission.pdf]

Reviewer Report

Title: The telomere-to-telomere (T2T) genome provides insights into the evolution of specialized centromere sequences in sandalwood

Version: Original Submission **Date:** 7/24/2024

Reviewer name: Chao Bian, ph.D

Reviewer Comments to Author:

This study entitled 'The telomere-to-telomere (T2T) genome provides insights into the evolution of specialized centromere sequences in sandalwood' has provided a T2T genome of sandalwood. This genome assembly is pretty well, and it contains all 20 telomeres and 10 centromeres. I think this data needs to be shown in the abstract.
On the other hand, this paper introduced a T2T genome and focused on the repeat sequences and their impacts on genome and expression.
But for this sandalwood genome, I still did not find any analysis for the biology points for this species. Why the author sequenced this species? Only want to show a T2T genome?
For the figure5, it's better to perform this analysis among more species.
For annotation methods, the detailed parameters should be introduced.
Line 60, The typeface of kiwifruit was different from all other words.
Line 97, Add blank between furniture and [21].
Line 101, sequence should be sequences.
Line 102, assembly should be assemblies.
Line 136, to should be onto.
Line 166, sequence should be sequences.
Line 309, between should be among.
Line 320, CDS should be defined. In first time.

Methods

Are the methods appropriate to the aims of the study, are they well described, and are necessary controls included? Choose an item.

Conclusions

Are the conclusions adequately supported by the data shown? Choose an item.

Reporting Standards

Does the manuscript adhere to the journal's guidelines on minimum standards of reporting? Choose an item.

Choose an item.

Statistics

Are you able to assess all statistics in the manuscript, including the appropriateness of statistical tests used? Choose an item.

Quality of Written English

Please indicate the quality of language in the manuscript: Choose an item.

Declaration of Competing Interests

Please complete a declaration of competing interests, considering the following questions:

- Have you in the past five years received reimbursements, fees, funding, or salary from an organisation that may in any way gain or lose financially from the publication of this manuscript, either now or in the future?
- Do you hold any stocks or shares in an organisation that may in any way gain or lose financially from the publication of this manuscript, either now or in the future?
- Do you hold or are you currently applying for any patents relating to the content of the manuscript?
- Have you received reimbursements, fees, funding, or salary from an organization that holds or has applied for patents relating to the content of the manuscript?
- Do you have any other financial competing interests?
- Do you have any non-financial competing interests in relation to this paper?

If you can answer no to all of the above, write 'I declare that I have no competing interests' below. If your reply is yes to any, please give details below.

no

I agree to the open peer review policy of the journal. I understand that my name will be included on my report to the authors and, if the manuscript is accepted for publication, my named report including any attachments I upload will be posted on the website along with the authors' responses. I agree for my report to be made available under an Open Access Creative Commons CC-BY license (http://creativecommons.org/licenses/by/4.0/). I understand that any comments which I do not wish to be included in my named report can be included as confidential comments to the editors, which will not be published.

Choose an item.

To further support our reviewers, we have joined with Publons, where you can gain additional credit to further highlight your hard work (see: https://publons.com/journal/530/gigascience). On publication of this paper, your review will be automatically added to Publons, you can then choose whether or not to claim your Publons credit. I understand this statement.

Yes Choose an item.
